# Schisandrins as novel efflux pumps inhibitors and non-antibiotic compounds against multi- and extensively-drug resistant clinical strains of *Salmonella typhi*: An *in-vitro* study

**DOI:** 10.1371/journal.pone.0347214

**Published:** 2026-07-31

**Authors:** Iqra Shafique, Nosheen Fatima Rana, Naseer Ahmad, Tahreem Tanweer, Sabah Javaid, Fawzyah Obeedallah Albaldi, Serag Eldin I. Elbehairi, Ali A. Shati, Huda M. Sheikh, Farid Menaa

**Affiliations:** 1 Department of Biomedical Engineering and Sciences, School of Mechanical Manufacturing Engineering, National University of Science Technology, Islamabad, Pakistan; 2 Excel Labs - Blue Area -, Islamabad, Pakistan; 3 Department of Biology, Faculty of Science, Al-Baha University, Alaqiq, Saudi Arabia; 4 Faculty of Science, Biology Department, King Khalid University, Abha, Saudi Arabia; 5 Department of Biological Sciences, College of Science, University of Jeddah, Jeddah, Saudi Arabia; 6 Department of Biomedical and Bioenvironmental Engineering (BEE), California Innovations Corporation (CIC), San Diego, California, United States of America; De Montfort University Faculty of Health and Life Sciences, UNITED KINGDOM OF GREAT BRITAIN AND NORTHERN IRELAND

## Abstract

**Purpose:**

Typhoid is a significant global health challenge due to its high pathogenicity and antimicrobial resistance. *Salmonella typhi* (*S.typhi*) can switch its lifestyles between biofilm and planktonic phase which allows it to evade host defenses and develop resistance to antibiotics. *Salmonella* sp. harbors multiple genes encoding efflux-pumps systems whose up-regulation contributes to multi-drug resistance (MDR) and extensive drug-resistance (XDR). To overcome the battle against resistant *S. typhi* strains, novel non-antibiotics inhibitors are required for inhibitory application. This study assesses the inhibitory effect of lignans against drug resistance of *S. typhi*.

**Methods:**

Clinical resistant and sensitive strains of *S. typhi* were obtained and characterized. The inhibitory effect of lignans, specifically Schisandrin A and B, purified from the plant *Schisandra chinensis*, are found to be effective non-antibiotic inhibitors were evaluated through standard microbiological techniques like growth curve and time-kill assays. Impact on bacterial morphology was analyzed using scanning electron microscopy (SEM). Our study explores two approaches, such as efflux pumps (EPs) inhibition and antibiofilm assays.

**Results:**

Using colony-forming unit (CFU) assays, growth curve analysis, and SEM imaging, we observed significant bacteriostatic effects, with Schisandrin B causing notable membrane disruption. Schisandrin B also showed remarkable biofilm inhibition (90.33%) and strong efflux pumps inhibition.

**Conclusion:**

This study offers a strong basis for future research on addressing antibiotic resistance in clinically relevant pathogens.

## 1. Introduction

Typhoid is an infectious disease predominantly caused by *Salmonella enterica* serovar *typhi* [1]. It infects humans and is acquired through contaminated food or water consumption. Common clinical manifestations involve elevated body temperature, nausea, abdominal pain, and diarrhea [2,3]. *S. typhi* is accountable for approximately 76.3% of typhoid cases. Globally, typhoid is estimated to range involving 11–27 million cases annually resulting in 128,000–161,000 deaths. In 2017, Global Health Data Exchange (GHDx) reported around 116,800 typhoid related deaths, with approximately 79,000 of these occurring in South Asia, including 6,700 in Pakistan [4,5].

Despite major advances in medicine, a considerable global population remains susceptible to typhoid fever [6]. In developing nations, antibiotic regimens are commonly deployed for typhoid management. However, its widespread use introduces the risk of adverse effects which promotes the emergence of antimicrobial resistant strains [7]. Antimicrobial resistance (AMR) poses a serious and escalating threat to global health, as it compromises the effectiveness of antibiotics which are essential for infectious disease treatments. Recently World Health Organization (WHO) (2024) suggest that AMR contributes to approximately 1.27 million deaths annually, with low- and middle-income countries facing the highest *S. typhi* identifies as H58 MDR lineage, that has emerged and spread all over Asia and Africa from the last 30 years Since 2016, Pakistan has faced the outbreak of typhoid, driven by the emergence of XDR strain, [6,8,9]. In the recent years, studies revealed high *S. typhi* infection rate in the country, with approximately 493.5 cases per 100,000 population and thousands of cases of XDR typhoid cases reported in Sindh, a southeastern region of Pakistan, and in Lahore, a northeastern region of Pakistan [10–13]. The global rise of multidrug-resistant (MDR) microorganisms causing infections is accelerating, with the problem being particularly severe in developing countries [14,15].

MDR strain are known to resist lethal doses of antibiotics drugs through a mechanism involving multidrug efflux pumps (EPs) [16]. It is worth noting that XDR strain even more problematic, since they are resistant to almost all the approved antimicrobial agents, leaving very few therapeutic options [17–19]. This may be explained by the fact that, in addition to increased efflux of drug by EPs, pathogenic bacteria can employ diverse other mechanisms of resistance including alteration of targets and inactivation of drugs.

In Pakistan, the rising occurrence of typhoid is attributed to MDR and XDR *S. typhi* raises concerned about the efficacy of antibiotic interventions [20]. EPs, predominantly recognized in bacteria for conferring MDR [21], play a vital role in antimicrobial resistance by expelling antimicrobial drugs from bacterial cell, by lowering the intracellular accumulation of toxic antibiotic levels, and conferring non-susceptibility in gram-negative pathogens [22]. Beyond drug extrusion, bacterial EPs are also involved in biofilm formation and maintaining cellular physiological activities, EPs also play important role in bacterial pathogenicity, virulence, and cell-to-cell communication [23,24]. Over expressed EPs influence the formation of biofilm by promoting the initial microbial adherence, extracellular matrix (ECM) production, transport of metabolites, quorum sensing (QS), and biofilm-associated genes indirectly [22]. Prokaryotic organisms exhibit five superfamilies of MDR EPs, contributing to the onset of MDR [25,26]. Notably, the ATP binding cassette (ABC) transporters are crucial plasma membrane proteins. They are prevalent across all living cells and intermittently in Gram-negative bacteria [27]. Thus, efflux pump inhibitors (EPIs) serve as a promising solution to overcome MDR, as well as bacterial virulence and biofilm formation [28].The increasing failure of conventional antibiotics against *S. typhi* highlights the urgent need for alternative, non-antibiotic therapeutic strategies, including novel antimicrobial compounds derived from natural or synthetic sources. These alternatives are particularly crucial in the face of rising resistance to third-generation antibiotics, which are the standard treatment options for typhoid fever in many endemic regions. Considering this, there is an urgent need to explore non-antibiotic novel compounds that could serve as alternative or adjunct therapies to combat drug-resistant *S. typhi* infection.

Phytocompound-based EPIs play an important role in inhibiting drug efflux and increase lethality at lower dosages [29]. On the other hand, they are able to disrupt biofilm formation by blocking the transport of QS-signal molecules [30]. Some research groups have reported the efficacy of natural compounds, such as lignans, flavonoids, alkaloids, to inhibit outer membrane protein in MDR cells lines. Naturally occurring lignans, such as Schisandrin compounds, originating from the family of *Schisandra chinensis*, significantly inhibit the P-glycoprotein (P-gp), an ABC transporter family member also encountered in various drug-resistant cancer cell lines [31]. Reported lignans such as Schisandrin (SA) and Schisandrin (SB) were shown to elicit anti-inflammatory, anticancer, antioxidant, as well as antibacterial activities [31–34]. Although their anti-inflammatory, antioxidant and anticancer activities have been explored, there is still a gap in understanding their antibiofilm, inhibitory activities and inhibitory mechanisms against typhoid specifically *S. typhi*. In our previous study, SA and SB were predicted as effective inhibitors against EPs, i.e., ABC-TPA, in *S. typhi* through *in silico* studies [32]. Keeping these results in view, and for further validation of our previous results, SA and SB were explored for their role as EPIs against clinical isolates of *S. typhi.* Based on previous results [35], we hypothesized that non antibiotic compound SA and SB can effectively inhibit EPs in MDR and XDR *S. typhi* strains. A non-antibiotic novel compound exhibits significant antimicrobial activity against drug-resistant strains of *S. typhi* and can serve as a promising alternative therapeutic agent in combating resistant typhoid fever.

The present study was conducted to validate the capacity of SA and SB as novel non-antibiotic compounds to enhance the treatment efficacy against *S. typhi*. For this purpose, our main objectives were two folds: (i) isolating and identifying MDR and XDR strains, and (ii) utilizing the SA and SB against these strains to evaluate the inhibitory effects against *S. typhi* EPs.

## 2. Methodology

All the chemicals were purchased from Sigma Aldrich (Hamburg, Germany). Prior to the commencement of the study, ethical approval was secured from the institutional review board (IRB #: 24-IRB-A-27/27 on May 29^th^, 2024) of the National University of Sciences and technology (NUST), Islamabad, Pakistan.

### 2.1. Sample collection

Blood samples were collected from patients suspected of having typhoid fever over a 10-day period, from July 1 to July 10, 2024 and identified as *Salmonella typhi* with a specific emphasis on assessing drug resistance profiles using Kirby-Bauer method. Ethylenediamine tetra-acetic acid (EDTA) tubes were utilized to collect blood samples and tubes were stored at 4°C.

### 2.2. Isolation and culturing

The blood samples were collected in blood culture bottles containing growth-supportive media. The inoculated bottles were incubated in a Vitek 2 Compact system, which monitored bacterial growth according to the manufacturer’s guidelines. Upon detecting positive cultures, a subculture was performed by transferring drops of the culture onto MacConkey agar plates and left for overnight incubation at 37°C. Non-lactose fermented colonies were isolated, and the oxidase test was then performed and those that tested negative were confirmed to be motile, Gram -negative rods [33].

### 2.3. Biochemical testing

The isolates were further identified through the Analytical Profile Index (API 10E) system, which provided the biochemical profile of identified *S. typhi*. Colonies were characterized according to their morphological characteristics subsequently undergoing a comprehensive analysis of biochemical evaluations utilizing the API 10E system (bioMerieux, France) for detailed identification. The biochemical evaluations were performed to determine the specific metabolic and enzymatic features including the oxidase test, arabinose fermentation, ornithine decarboxylase (ODC) test, indole formation (IND), citrate utilization, glucose fermentation (GLU) testing, tryptophan deaminase (TDA) assessment, urease (URE) function lysine decarboxylase (LDC) activity, hydrogen sulfide (H_2_S) production and the nitrophenyl-b-D-galactopyranoside (ONPG) reaction. The colonies were incubated for a duration of 18–24 hours at 37°C to facilitate observable biochemical reaction thereby enhancing the precise identification and characterization of the bacterial isolates [11].

### 2.4. Kirby Bauer method

Antibiotic susceptibility and resistance were identified by utilizing the Kirby Bauer disk diffusion method. Isolated colonies were cultivated on tryptic soy agar for 24 hours of incubation at 37°C. Diluted suspensions of *S. typhi* isolates matching with McFarland 0.5 turbidity standards. The bacterial suspensions were applied uniformly on TSA and allowed to adhere for a period of 10 minutes. Subsequently antibiotic discs from Oxoid (Thermo Fisher Scientific) were then positioned on the TSA plates placed at incubator at 37°C for 24 hours [7,34,35].

The antibiotics tested included ampicillin (10 μg), chloramphenicol (30 μg), ciprofloxacin (5 μg), sulfamethoxazole/trimethoprim (SXT, 25 μg), imipenem (10 μg per disk), meropenem (10 μg), azithromycin (15 μg), and ceftriaxone (30 μ g) per disk. Each disc was placed on the surface of TSA separately and incubated overnight at 37°C. Zone of inhibition were measured [36].

### 2.5. Ethidium bromide (EtBr)-agar cartwheel (EtBrCW) assay

The EtBrCW assay was utilized to evaluate the efflux pump activity based on accumulation of EtBr (fluorescent probe) within the bacterial cells. The clinical bacterial isolates including MDR, XDR and control (sensitive isolate) were cultured at 37°C with agitation for 24 hours. Tryptic soy agar (TSA) medium with 2.5 μg/mL EtBr was prepared and bacterial isolates were swabbed onto agar plates before their incubation for 16–18 hours at 37°C. The plates were visualized using UV transilluminator (Biorad, Dubai, UAE) at the wavelength (λ)= 302 nm. Then images of plates were captured through gel documentation system (Bio-Rad Gel Doc EZ Imager, Dubai, UAE) [37–39].

### 2.6. Biofilm formation of MDR and XDR *S. typhi* isolates

#### 2.6.1. *Air-liquid interface assay.*

This qualitative assay [40,41] was utilized to evaluate the biofilm-forming potential of the MDR and XDR *S. typhi* isolates. In this assay, cultures of isolates were inoculated in TSB in each well of the 12-well plates. Coverslips were placed at 90° angle in the incubator for 48 hours at 37°C. For this, during a period of this incubation, after the biofilm adhered to the coverslip surface, the coverslips were lifted carefully in a vertical orientation utilizing sterilized forceps, while minimizing any disturbance to the biofilm layer that has been formed. Subsequently, the coverslips underwent a gentle washing process with distilled water by employing a method of gradual dipping and lifting for each coverslip. The coverslips were then placed within another 12-well plate for staining with 0.1% crystal violet solution. This procedure was conducted for 15 minutes at room temperature (RT), permitting sufficient time for the crystal violet to effectively bind to the biofilm without any form of agitation. The distilled water was used to clean the coverslips. High-power light microscope (Zeiss, Oberkochen, Germany) was used to visualize the coverslips [40,41].

#### 2.6.2. *Spectrometric analysis of biofilm formation.*

In this quantitative assay [40,41], bacterial cultures were adjusted to attain the 0.5 McFarland index which corresponds to approximately 1.5 × 10⁸ CFU/mL. The bacterial cultures were inoculated in TSB in 96-well plate and incubated overnight for 24 hours. A well with media served as negative control. After incubation, the planktonic (free-floating) cells were gently removed and washed thrice with phosphate-buffered saline (PBS) before adding 0.1% crystal violet solution to each well and incubated for 10−15 minutes at RT to stain the biofilm. After incubation, the wells were carefully washed thrice with PBS to remove excess stain, leaving only the stained biofilm attached to the well surfaces. Then, 95% ethanol was added to each well to solubilize the crystal violet bound to the biofilm, and the optical density (OD) at λ = 630 nm was measured. The biofilm formation was scored according to the OD value and following interpretation: (0.0–0.5) meant weak biofilm, (0.5) signified moderate biofilm, and (> 0.5) indicated strong biofilm [40,41].

### 2.7. Evaluation of intrinsic antimicrobial activity of Schisandrins A (SA) and B (SB)

#### 2.7.1. *Efflux pump inhibition (EPI) assay.*

Bacterial inoculum was dispensed into wells of a sterile black 96-well microtiter plate. SA and SB (20) were loaded into columns 1–4, while 100 μM EtBr was added to every well. Dimethylsulfoxide (DMSO) (control) was added in column 5. The fluorescence of accumulated EtBr was then examined using a fluorescence spectroscopy (Fl6500, Perkin USA)at λ = 530 nm and λ = 600 nm [42].

#### 2.7.2. *Determination of minimum inhibitory concentration.*

The MICs of SA and SB against MDR and XDR strains were determined using broth dilution method. A stock solution (1 mg/mL) of SA and SB was serially diluted in test tubes to achieve concentrations of 0.5, 1, 3, 5, 10, 20, and 40 µg/mL. Each solution was inoculated with 50 µl of bacteria innoculum. TSB used as control was also inoculated with bacterial isolates. The plate was placed in the incubator for 6 hours at 37°C and after that visually examining the turbidity under good lighting with visible cloudiness indicates the bacterial growth proliferation. After 6 hours of incubation the lowest concentration at which maximum growth inhibition occurred was considered as the MIC [45,43,44].

#### 2.7.3. *Growth curve analysis.*

The growth patterns of *S. typhi* strains were examined after exposure to SA and SB to quantify their intrinsic antibacterial efficacy. After the 4 hours of culturing overnight MDR and XDR *S. typhi* cultures at 37°C cultures were then suspended and SA and SB were added at concentrations of (0.5, 1, 3, 5, 10, 20, 40 µg/mL). Subsequent incubation at 37°C in an orbital shaker incubator at 160 rpm. Control cultures of MDR and XDR without additives were included and [12] at 600 nm was measured at hourly intervals for up to 8 hours. Graphical representations were performed using GraphPad Prism 9 [46,47].

#### 2.7.4. *Colony forming unit (CFU) analysis by time kill assay.*

Time-kill analysis was conducted using a previously reported method [43,48,49]. First, *S. typhi* strains were sub-cultured and diluted. Then, different concentrations of 3, 5, 10, and 20 µg/mL of SA and SB was added in to sterile TSB, along with bacterial inoculum. The samples were placed in the incubator for 6 hours at 37°C. After that the collected aliquots were inoculated onto TSA and incubated at 37°C for overnight. CFU was then calculated. Finally, a log cfu/mL versus time graph was plotted using graph pad prims to analyze bacterial growth dynamics with data derived from the growth curve assay to assess microbial proliferation over time [43,48,49].

#### 2.7.5. *Effect of Schisandrins on morphology by scanning electron microscopy* (SEM).

The morphology of untreated and treated MDR and XDR and sensitive *S. typhi* isolates were subjected to SEM imaging (41, 44). Isolates were inoculated into TSB and incubated for 12 hours (overnight). SA and SB were then introduced at a final concentration of (2 × MIC) for 6 hours. Control samples were left untreated. After centrifugation of the samples, 3% glutaraldehyde (GA) was used to fix the sediment cells, which were dehydrated with a series of ethanol concentrations and then dried to a critical point. The dried cells were coated with gold and bacterial cell morphologies were observed using a VEGA3 LMU SEM apparatus (Tescan, Brno, Czech Republic) [41,45,50].

#### 2.7.6. *Antibiofilm activity of the Schisandrin compounds.*

The anti-biofilm properties of SA and SB were evaluated through a microtiter plate spectroscopic assay [41,43,44]. Fresh cultures of isolates were cultured in TSB overnight followed by dilution in fresh TSB. The control well contained DMSO solvent at a concentration equivalent to that of treated with the compounds. The microplate was then incubated for 24 hours at 37°C. The liquid (medium or DMSO) was removed carefully without disturbing the biofilm attached to the well surface. Wells were gently rinsed followed by staining with crystal violet solution (0.1%) for 20 minutes. Following staining, the wells underwent another round of washing and were air dried. Afterward, 95% ethanol was added to the wells and and solution was incubated for 15 minutes at room temperature. After that, (150 µl) of solution was gently transferred to a separate 96-well plate. The OD was then calculated at 630 nm using a microplate reader. The biofilm inhibition was measured using the following formula (equation 1):

$\frac{(\text{ODc}-\text{ODb})\;-\;(\text{ODt}-\text{ODb})\;}{\;(\text{ODc}-\text{ODb})}\times \text{100}\%$

Where, ODc represents the absorbance of non-treated isolates, ODb stands for absorbance of TSB (culture medium), and ODt is the absorbance of treated isolates.

## 3. Results

### 3.1. Isolation and identification of *S. typhi* isolates

Blood samples were obtained from patients suspected of having typhoid at Excel Lab Islamabad Pakistan. The samples were cultured on MacConkey agar placed in incubator at 37°C for 24 hours. Samples showed growth in the MacConkey agar media indicating the presence of bacteria-lactose fermenting colonies consistent with *Salmonella spp.* were observed.

### 3.2. Biochemical identification of the *S. typhi* isolates

**Fig 1:**
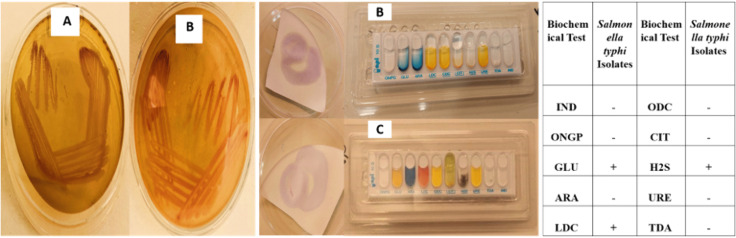
Isolation and biochemical identification of *S. typhi* isolates through API 10S (A) Selective plating on MacConkey agar, (B) Oxidase Test before the Incubation and API 10S Before the incubation, (C) Oxidase test become negative and API 10S after the incubation.

The biochemical identification of *S. typhi* isolates as identified through the API 10E system (bioMerieux, France), provides a clear distinction from other enteric bacteria and is crucial for accurate pathogen identification. The negative reactions for oxidase, ARA, ODC, TDA, IND, URE, and citrate utilization indicate that these isolates lack certain enzymes and metabolic pathways, differentiating them from organisms like *Proteus* (urease-positive), *Pseudomonas* (oxidase-positive), and *Klebsiella* (citrate-positive). In contrast, the positive results for glucose (GLU) fermentation, lysine decarboxylase (LDC) activity, and hydrogen sulfide (H_2_S) production are indicative markers of *S. typhi*, reflecting its ability to produce acid from glucose metabolism to decarboxylate lysine into cadaverine, and to produce hydrogen sulfide, which creates a black precipitate in culture media. Additionally, the isolates’ inability to hydrolyze ONPG, shown by a negative ONPG reaction, confirms the absence of β-galactosidase consistent with *S. typhi*’s known trait as a non-lactose fermenter. These results indicate a specific metabolic profile that not only verifies the identity of *S. typhi* but also aids in distinguishing it from similar organisms (Fig 1).

### 3.3. Kirby-Bauer method

**Fig 2:**
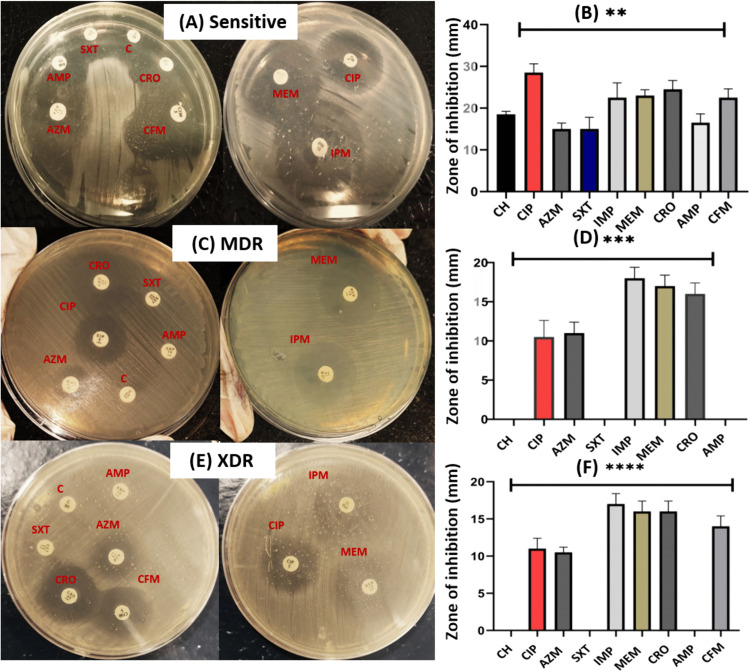
Identification of drug resistance through disk diffusion assay (DDA) and bar graph analysis of *Salmonella typhi* isolates (A) and (B) Sensitive strain, (C) and (D) MDR strain, and (E) and (F) XDR strain.

Eight antibiotics from different generations were used to assess antibiotic resistance levels in *S. typhi* isolates. Out of the four tested isolates, one isolate was identified as MDR, displaying resistance to chloramphenicol, sulfamethoxazole/trimethoprim, ampicillin, and ciprofloxacin. Another isolate was classified as XDR due to its resistance to the aforementioned antibiotics, including Ceftriazone. Two isolates of *S. typhi* were found to be sensitive, exhibiting susceptibility to all tested antibiotics. The resistance profiles of *S. typhi* isolates to the nine different antibiotics are summarized in (Fig 2).

### 3.4. Detection of efflux pump through ETBr-agar cartwheel (EtBrCW) assay

**Fig 3:**
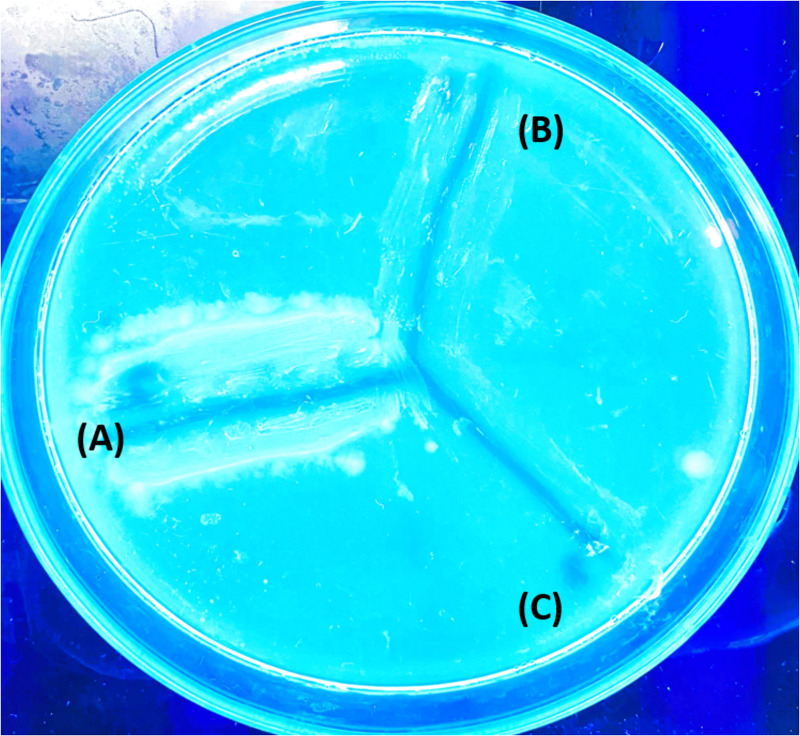
EtBr-agar cartwheel (EtBrCW) assay of isolated *S. typhi* strains (A) in sensitive strain (control). (B) in MDR, and (C) in XDR strain, and (C) in sensitive strain (control).

The EtBrCW assay was utilized to detect the efflux pump activity in *S. typhi* strains by directly visualizing the fluorescence based on EtBr accumulation (Fig 1). The intensity of fluorescence was weak in MDR (Fig 3B) and was absent in XDR (Fig 3C) strains, whereas it was high in sensitive strain of *S. typhi* used as control (Fig 3A).

These data indicate actively expressed efflux pumps in the test isolates. Also, the fluorescence observed in control indicated that the strain is unable to efficiently expel EtBr, leading to its accumulation within the bacterial cells. Indeed, accumulated EtBr binds to DNA which caused an increase in fluorescence intensity because EtBr is an intercalating agent that inserts itself between DNA base pairs causing it to fluorescence more brightly upon binding.

### 3.5. EtBr efflux pump inhibition (EPI) assay

**Fig 4:**
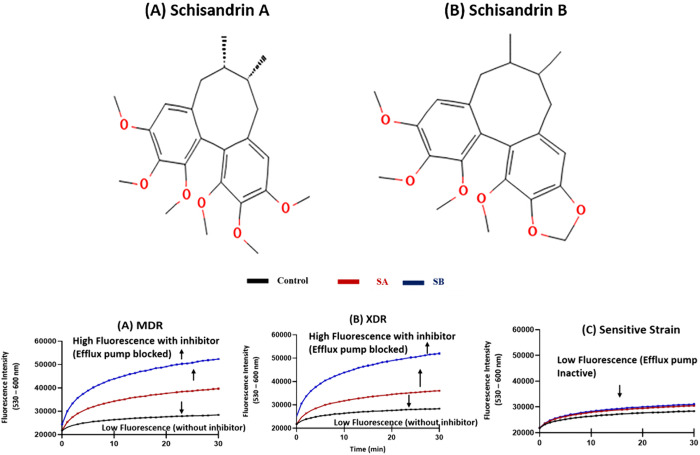
SA and SB structure and efflux pump inhibition (EPI) effects of Schisandrin A (SA) and Schisandrin B (SB) against MDR, XDR and sensitive *S. typhi* isolates. (A) in MDR strain, (B) in XDR strain, and (C) in sensitive strain (control). SA and SB were used at the same concentration (100 μM).

We have adapted an existing fluorescence assay using EtBr (100 μM) to monitor overtime accumulation (efflux inhibition) activities of *S. typhi* strains connected to lignans SA/SB resistance/tolerance (Fig 4). EtBr acts as a substrate of efflux pumps and it is expelled from the cells when EPs are actively overexpressed. Conversely, when the EPs are inhibited then EtBr is accumulated within the cells. The efflux of EtBr is presented in terms of fluorescence intensity, which is obtained for the MDR (Fig 4A), XDR (Fig 4B), and the sensitive strains (Fig 4C), in the presence or absence (control) of lignans (SA or SB) (31). Fluorescence spectroscopy (PerkinElmer FL6500) (USA) equipped with a 150 W Xenon arc lamp was used to measure the fluorescence intensity at the (600 nm) emission and (530 nm) excitation nm at a scan speed of 1200 nm/min provided a reliable measure of the potential efflux of EtBr from the bacterial cells.

Before exposition to lignans, the weakly increased fluorescence intensity overtime observed in the test strains is similar to that of the fluorescence intensity observed in the sensitive strain, indicating that the EtBr is slightly accumulated within the bacterial cells within 30 min.

After exposition to SA or SB, significant increases (P < 0.05) in fluorescence intensity are observed overtime in MDR and XDR strains compared to that of the sensitive strain. SA exerted a better effect (P < 0.05) in MDR compared to that of XDR, while SB elicited a similar effect in both resistant strains. The effect of SB was higher (P < 0.05) compared to that of SA, when evaluated in both MDR and XDR strains. SA or SB did not display any significant effect (P > 0.05) in the sensitive strain, which contributes to understand mechanistically drug-sensitiveness of this strain.

Indeed, in 3 minutes, both SA and SB started with similar EtBr fluorescence intensities around 25,000, independently of the strain. However, by 10 minutes, the EtBr fluorescence intensity reached around 34298.6 for MDR and 31791.9 for XDR strains treated with SA (P < 0.05), whereas the EtBr fluorescence intensity has risen to approximately 43918.7 for MDR and 43883.9 for XDR strains treated with SB (P > 0.05). By 30 minutes, SA showed a more moderate increase (about 3800 for MDR, and 3200 for XDR) compared to SB (about 50000 for MDR and XDR) (P < 0.05).

Taken together, since SA or SB had no effect in the sensitive *S. typhi* strain contrarily to resistant *S. typhi* strains, we could confirm that *S. typhi* MDR and XDR strains use EPs as a mean to resist to chemical treatments; importantly, the lignans SA, and particularly SB, are potential EPIs to overcome that resistance. The lower fluorescence intensity and EtBr accumulation in the sensitive strain suggest that EPs are present, but they are less active than in the MDR and XDR strains. Statistical analysis was conducted using GraphPad Prism (Version 9, San Diego, CA, USA). All the experiments were statistically analysed using one-way ANOVA with significance set at *p* < 0.05. Replicate numbers (n = 3) and 95% confidence intervals.

### 3.6. Biofilm-formation potential of *S. typhi* isolates

**Fig 5:**
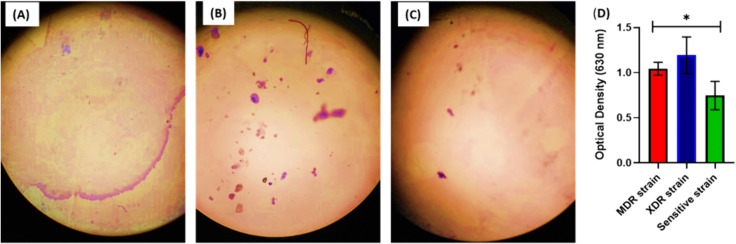
Qualitative and quantitative biofilm formation potential of *S. typhi* isolates. Cover slip assay of (A) in MDR strain, (B) in XDR strain, and (C) in a sensitive strain (control), and (D) spectrophotometric analysis.

The biofilm formation results indicated that the *S. typhi* isolates, either resistant and sensitive, have a strong potential for biofilm formation as it was evidenced by coverslip assay, from which staining and visible aggregation of bacterial colonies are observed (Figs 5A-5C). The quantitative assay showed significant biofilm formation with OD 630 nm values above 0.5 (Fig 5D), classifying these isolates as strong biofilm forming potential according to criteria described in methods.

Actually, the MDR and XDR isolates showed a significantly higher biofilm-forming potential with a mean value of 1.0 and 1.2, respectively, while the sensitive isolate used as control showed an OD value of 0.8, all falling within the strong biofilm category when compared to that of the sensitive isolate used as control showed the OD values of 0.8, falling with in the moderate biofilm category (p = 0.003). There was also a significant difference (P < 0.05) in biofilm formation when test isolates are compared to each other.

These results suggested that the enhanced biofilm formation in the XDR isolate may contribute to its increased resistance. Resistant isolate often demonstrates altered cell surface properties such as enhanced production of EPs, which are known to be important components for biofilm formation and stability.

### 3.7. Minimum inhibitory concentration analysis of Schisandrin A and B against *S. typhi* isolates

The growth inhibition effect of SA and SB against MDR and XDR *S. typhi* was analyzed to evaluate the effect of SA and SB against the MDR and XDR *S. typhi* through different microbiological assays. The MIC analysis showed the growth at a range of 5–10 µg/mL demonstrated the lowest concentration at which no visible growth was observed.

### 3.8. Time kill assay

**Fig 6:**
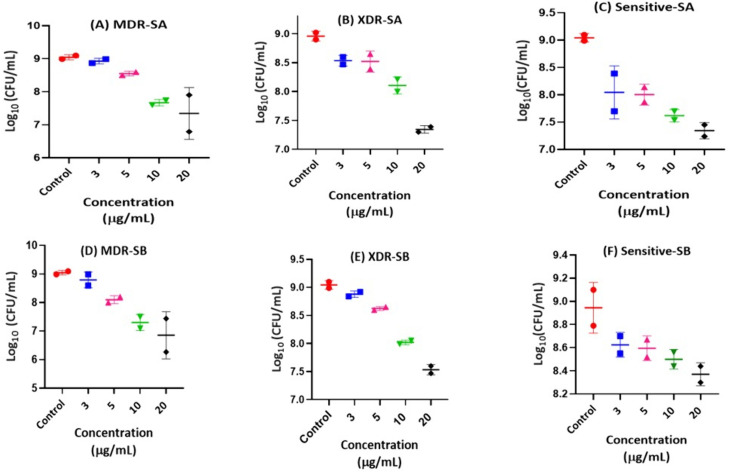
CFU analysis of MDR and XDR *S. typhi* by Time kill assay after 6 hours. (A) CFU analysis of MDR *S. typhi* with SA (B) CFU analysis of MDR *S. typhi* with SB, (C) CFU analysis of XDR *S. typhi* with (D) CFU analysis of XDR *S. typhi* with SB.

The time kill analysis was utilized to ensure the efficacy of SA and SB against bacterial CFU counts. It showed a decrease in CFU of the bacteria with an increase in concentration of SA and SB for MDR isolate (Fig 6A and 6D), XDR *S. typhi* isolate (Fig 6B and 6E) and sensitive isolate (Fig 6C and 6F). Drug SA exhibits effectiveness against both MDR, XDR and sensitive isolates. For MDR bacteria, the mean CFU/ mL values decrease from 9.045 (SD = 0.07750) to 3 µg/mLlto 8.540 (SD = 0.000) at 20 µg/mL, with (p-value = 0.0027). For XDR bacteria, the mean values decrease from 8.961 (SD = 0.08556) at 5 µg/mL to 8.045 (SD = 0.06364) at 20 µg/ml, showing a similar pattern (p-value = 0.0135). For Sensitive isolate the mean values decrease from 8.03 (SD = 0.4879) to 3 µg/mL to 7.34 (SD = 0.1485) at 20 µg/mL, with (p-value = 0.0072). These results suggested that increasing the concentration of drug SA beyond a certain point does not significantly enhance its antibacterial effect as indicated by the statistically significant p-values. Drug SB shows a gradual decline against MDR, XDR and sensitive isolates as the concentration increases. For MDR bacteria, the mean CFU/ml values decrease from 9.045 (SD = 0.07750) at 3 µg/mL to 8.085 (SD = 0.1202) at 20 µg/mL (p-value = 0.0088). A similar trend was observed against XDR bacteria with a statistically significant p-value of 0.0032. The mean CFU/mL values drop from 9.045 (SD = 0.07750) to 3 µg/mL to 8.535 (SD = 0.09192) at 20 µg/mL. For sensitive isolate the mean value decreases from 8.625 (SD = 0.1061) at 3 µg/mL to 8.370 (SD = 0.098 with p-value = 0.0497).

### 3.9. Growth curve analysis

**Fig 7:**
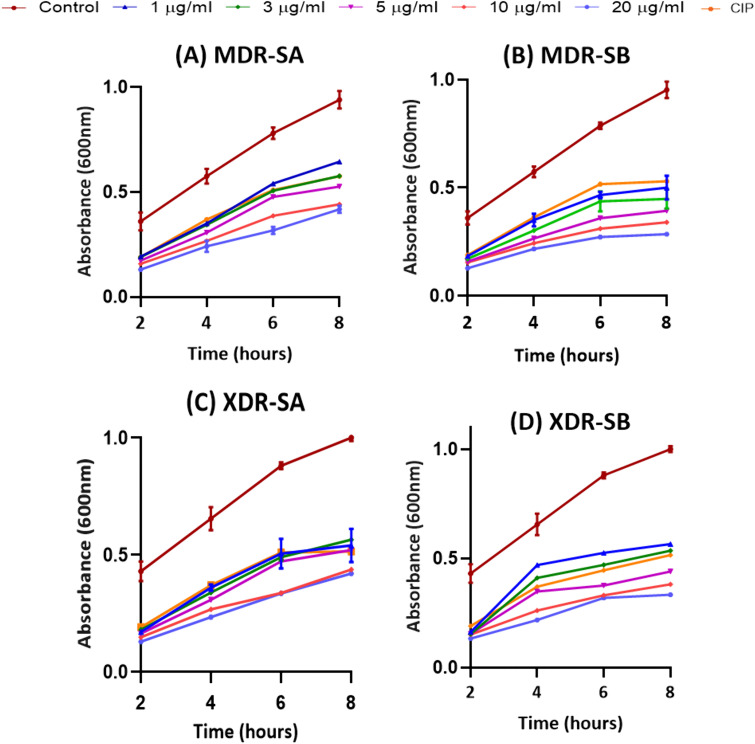
Growth effect of SA and SB against *S. typhi* strains (A) Growth effect of MDR and XDR *S. typhi* with SA (B) Growth curve of MDR and XDR *S. typhi* with SB.

The growth curve analysis showed that the increase in concentration of SA and SB the bacterial growth curve was found to decrease over time, as shown in (Fig 7). The growth curve was found to be equivalent of control at lowest concentration of 1 µg/mL. These results indicated that as the concentration of SA and SB increased from 5 µg/mL to 20 µg/mL, there was a noticeable and consistent inhibition of bacterial growth. This suggests that SA and SB have a bacteriostatic effect, meaning they can inhibit the growth and reproduction of bacteria without necessarily killing them. These findings highlight the SA and SB as potential non antibiotic inhibitors, particularly in controlling bacterial growth. The observed decline in bacterial growth after 6 hours suggests that the compounds exerted their bacteriostatic effect over time.

### 3.10. Impact of Schisandrin compounds on the morphology of *S. typhi* cells

**Fig 8:**
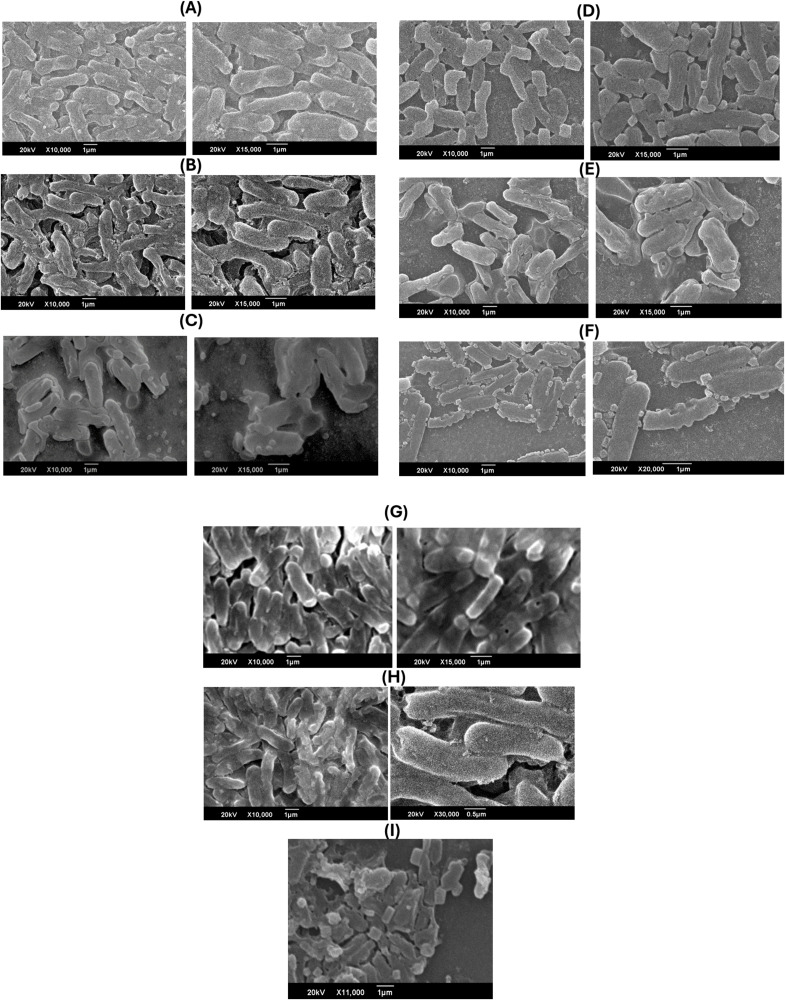
Scanning electron microscopy (SEM) images of *S. typhi* isolates. (A-C) in MDR strain, (D-F) in XDR strain, and (G-I) in sensitive strain, with: (A, D, G) untreated (control), (B, E, H) treated with SA, (C, F, I) treated with SB. SA and SB were used at 2 X MIC for 6 hours. Magnifications and scale bars are indicated.

SEM analyses were performed on MDR, XRD and sensitive strains of *S. typhi* to visualize whether SA or SB has an impact on their cell morphology (Fig 8). Importantly, both SA and SB had notable effects on their morphology, but their impact differed significantly.

*S. typhi* cells that were treated with SA at 2 × MIC for 6 hours caused visible damage to the bacterial cells. This suggests that SA has a moderate effect on cell morphology (Fig 8B, 8E and 8H). In contrast, SB treatment under the same conditions resulted in severe damage to the bacterial cells. The cells exhibited more pronounced changes, including wrinkled and withered surfaces, along with membrane distortion and the disappearance of the outermost layer (Fig 8C, 8F and 8I) In the absence of SA and SB *S. typhi* cells were intact with regular surfaces and outer layers were intact (Fig 8A, 8D and 8G).

These findings imply that SB is more potent than SA in damaging the external structures of *S. typhi* cells.

### 3.11. Antibiofilm activity of SA and SB against MDR and XDR *S. typh*i isolates

**Fig 9:**
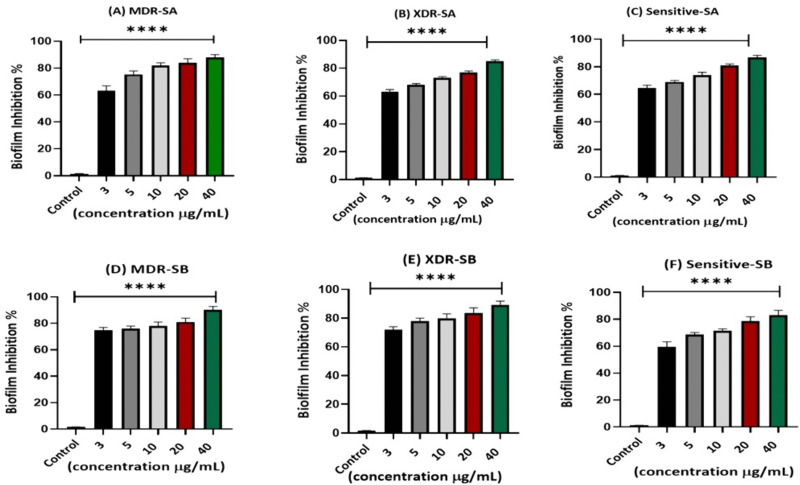
Antibiofilm activity (%) of Schisandrin A (SA) and Schisandrin B (SB) toward MDR and XDR *S. typhi* isolates. (A) SA against MDR *S. typhi,* (B) SB against MDR *S. typhi*, (B) SA against XDR *S. typhi*, (D) SB toward XDR *S. typhi.*

The quantitative biofilm assay was used to evaluate the antibiofilm potential of SA and SB, at various concentrations (i.e., 0, 3, 5, 10, 20, 40 µg/mL) against MDR and XDR *S. typhi* (Fig 9). Both drugs demonstrated significant inhibition of biofilm formation. SA showed mean inhibition percentages of 88.00% for MDR (Fig 9A), 85.00% for XDR (Fig 9B) and (Fig 9C) at 20–40 µg/mL, while SB exhibited slightly higher inhibition with 90.33% for MDR (Fig 9D) and 89.33% for XDR (Fig 9E) and 87% for sensitive isolate at the same concentration. The P-values of < 0.0001 for both drugs, compared to the control (untreated) suggest the strong efficacy of SA and SB with SB showing a marginally higher effectiveness (P > 0.005) in biofilm inhibition compared to that of SA.

## 4. Discussion

Despite the effectiveness of several antimicrobials against typhoid caused by *S. typhi*, the development of drug-resistant *S. typhi* strains complicates treatment [7].

In this study, we report the isolation and identification of clinical resistant *S. typhi* strains, using selective plating and biochemical analysis with a specific emphasis on EPs mechanisms. Such approach is necessary for advancing our understanding and overcoming the antimicrobial resistance [6,46].

Briefly, we employed the Kirby-Bauer disc diffusion method (DDM) and eight antibiotics of different generations to assess the AMR of the *S. typhi* isolates. The results revealed that high levels of resistance within the *S. typhi* isolates. Specifically, one isolate demonstrated MDR against chloramphenicol, sulfamethoxazole/trimethoprim, ampicillin, and ciprofloxacin. Another isolate exhibited XDR, showing resistance not only to the aforementioned antibiotics but also to Ceftriazone, thus severely limiting treatment options. Similar results for antibiotic resistance against *S. typhi* have been reported in previous studies [33,47–49,51,52]. Our study identified MDR and XDR *S. typhi* isolates, including resistance to Ceftriazone which severely restricts treatment options in typhoid-endemic areas. In comparison, the European Centre for Disease Prevention and Control (ECDC, 2024) reported declining MRSA bloodstream infections but a 58% increase in carbapenem-resistant *Klebsiella pneumoniae* and persistently high rates of cephalosporin-resistant *E. oli*. Similarly, the U.S. Centers for Disease Control and Prevention (CDC, 2023) noted a 20% rise in hospital-onset resistant infections during the pandemic. These contrasts show that while South Asia faces an enteric-fever resistance crisis, Europe and the U.S. struggle mainly with hospital-acquired Gram-negative pathogens, yet all regions share the urgent need for stronger stewardship, diagnostics, and infection-prevention measures [53,54].

The successful isolation and identification of resistant strains provided a foundation for EP detection through EtBr-agar cartwheel assay. This assay is based on EtBr accumulation by directly visualizing fluorescence to assess EPs activity [55,56]. Our findings revealed that the sensitive strain (control) showed the highest fluorescence due to (EtBr) accumulation resulting from low EPs activity. In contrast, the MDR and XDR strains exhibited the lowest fluorescence levels indicating high EP sactivity and overexpression. Similar findings using the same assay have been reported in previous studies [39,57].

Our subsequent investigations focused on assessing the EPIactivity of lignans SA and SB, based on our previous *in-silico* analysis of their activity (32) We comprehensively tested these lignans (Schisandrins) for their ability to inhibit EP using the EtBr accumulation assay. Our results clearly demonstrated EPI activities of both SA and SB on EtBr in MDR, XDR, and sensitive strains of *S. typhi*. SB was the most effective EPI inhibitor against *S. typhi* isolates as indicated by enhanced fluorescence intensity and EtBr accumulation in resistant isolates compared to the sensitive isolate. Indeed, SB more effectively led to enhanced EtBr accumulation inside bacterial cells compared to SA. The reduced fluorescence intensity and EtBr accumulation in the sensitive isolate suggested that EPIs are present, but they are less active as compared to MDR and XDR isolates. Our results were supported by a previously reported assay.

Moreover, we examined the biofilm forming capacity of *S. typhi* isolates which are frequently correlated with their AMR and virulence. Several studies have reported that the biofilm formation is associated with altered surface properties of cells in resistant isolates involving increased production of Eps [39,58–60]. An antibiofilm effect was clearly observed when increasing SA and SB concentrations which was associated with a decrease in CFU. We utilized the concentration that showed approximately 50% biofilm inhibition by following the approach of Kaur et al. (2025). SB exhibited stronger antibiofilm activity compared to that of SA. To understand whether this antibiofilm activity was due to bacteriostatic effect (reversible bacterial growth inhibition) or by bactericidal effect (irreversible bacterial growth inhibition/killing) effect, SEM analyses were performed to eye-naked visualize potential damages of the cell membrane of bacteria treated with SA and SB. The bacterial cell structures of the control (untreated) were intact when compared to that of lignans-treated strains, in which membrane damages and shrinkages were noticed. Further, the time-kill assay indicated a decline in bacterial growth after 6 hours, confirming the lignans-mediated bactericidal effect. SB showed a stronger antibacterial effect. Our findings emphasize the importance of SA and SB to overcome the MDR and XDR S. *typhi* infections through irreversible inhibition of bacterial growth.

Taken together, these *in-vitro* data demonstrated a dual function of the lignans SA and SB in the resistance and control of biofilm formation of (MDR and XDR) S. *typhi* strains. Indeed, the EPI effect of the lignans (SA and SB) was revealed through a mechanism involving drug retention into bacterial cytoplasm and DNA, and their ability of acting as antibiotics-like compounds was depicted through a mechanism involving the disruption of the bacterial cell membrane integrity. Similar findings were reported [61,62], including by the use of *S. chinensis* extract [63], from where SA and SB are originated. Although we previously showed that SA and SB could be effective inhibitors against EPs, i.e., ABC-TPA, in *S. typhi*, the present study confirms our hypothesis, and report for the first time the importance of these lignans to naturally overcome *S. typhi* resistance.

Recent studies have expanded the understanding of the antibacterial properties of *S. chinensis*, which have been reviewed recently [64]. Thereby, *S. chinensis* extracts were efficient in both inhibiting the growth of methicillin-resistant *Staphylococcus aureus* (MRSA) but also in disrupting its biofilm formation, a critical factor in MRSA’s resistance and persistence [64]. Moreover, the antibacterial activity of *S. chinensis* extracts against *Helicobacter pylori*, a bacterium associated with gastric ulcers and cancer, was prominent in inhibiting the growth of *H. pylori* and reducing the expression of virulence factors. In addition to whole extracts, recent notable studies are focusing on isolating specific compounds responsible for the antibacterial activity of *S. chinensis*. Purification of some of these compounds identified tartaric acid as a significant active compound against *Streptococcus mutans*, a primary bacterium responsible for dental caries [65]. In accordance with our present study, Liu et al., 2022, identified gomisin A and schisandrin B as potent antibacterial agents against various Gram-positive and Gram-negative bacteria, through a mechanism implicating the disruption of bacterial cell membranes, leading to cell lysis and death. Studies have also investigated the potential synergistic effects of *S. chinensis* extracts with conventional antibiotics. A study by Park et al., 2023, examined the combination of *S. chinensis* extract with ampicillin and found a synergistic effect against multidrug-resistant *Escherichia coli*. This combination therapy significantly reduced bacterial load compared to the antibiotic alone, suggesting that *S. chinensis* could enhance the efficacy of existing antibiotics and help combat antibiotic resistance [64]. Clinical trials are being conducted to assess the effectiveness of *S. chinensis* extracts in treating infections in humans. Preliminary results from a trial by Lee et al. 2023, indicated that the topical application of *S. chinensis* extract could effectively reduce symptoms of bacterial skin infections, highlighting its potential as a natural antimicrobial agent for dermatological use [64].

Although lignans, a sub-group of non-flavonoid polyphenols [66], are known to exert pleiotropic biological activities including potent antibacterial effects [67], there is still a paucity of reports in this regard. Also, to the best of our knowledge, no studies have deeply characterized SA or SB compounds, and no one of them have been explored to fight resistant strains of *S. typhi*. These overall promising findings against resistant clinical isolates of *S. typhi* underscore the importance of lignans SA and SB and *S. chinensis* extracts which should led to further explorations of its potential clinical applications.

## 5. Limitations of the study

Further explorations shall include (i) the potential additive or synergistic effects of SA and/or SB with conventional antibiotics, at pre-determined concentrations and time; (ii) the imaging/monitoring of *S. typhi* strains pre-immobilized in coverslips to study their behavior under different conditions or look at specific protein expression patterns during dynamic processes; (iii) the testing of SA and SB against a wide number of microbial and viral infections; (iv) pre-clinical studies using animal models of the typhoid fever; (v) We will explore molecular mechanisms, including gene identification (e.g., *blaCTX-M*, *blaNDM*) and plasmid typing, to provide a more comprehensive understanding of resistance transmission. EPI activity of the SA and SB were conducted in vitro and limited to specific bacterial strains, which may not fully represent the complexity of efflux systems in clinical settings. Molecular targets and inhibition mechanisms were not characterized, and in vivo testing was beyond the scope of this work. Therefore, while the results evaluate potential EPI activity, of SA and SB against different mechanistic pathways, broader strain panels, and animal models are required.

## 6. Conclusion

Herein, our study reveals the EPI and antibacterial activity of SA and SB. These lignans isolated and purified from *S. chinensis*, a plant widely available in Asia, have been explored for the first time against resistant (MDR and XDR) strains of *S. typhi*, responsible for the life-threatening typhoid fever which caused a recent outbreak in Pakistan. Despite the fact that Pakistan became the first country in the world to introduce the WHO-recommended typhoid conjugate vaccine (TCV) into its routine immunization program, it is worth mentioning that this effective solution is a short-term strategy due to its accessibility in low-middle countries (e.g., high cost, cultural and religious barriers toward vaccines) and inadequate practices in some regions (e.g., poor sanitation, contaminated water, overuse of antibiotics). To overcome the limited treatment of the population, and because the AMR leads to the emergence of newer, more resistant strains, it is essential to search for alternative therapeutic solutions. One of them is the constant search of naturally active compounds or phytoextracts exerting effective activity against *S. typhi* resistant strains. This work represents an important step toward the scalable production of SA and SB, which strongly demonstrates potency as EPI and natural antibiotics to overcome *in vitro* biofilm formation (and so, persistence and virulence) of resistant strains of *S. typhi* (both MDR and XDR). These findings highlight lignans, specifically SB, as an effective agent for biofilm disruption and their potential biomedical application as non-antibiotic therapeutic options to combat antibiotic resistant *S. typhi.* All assays were limited to in vitro analysis and a limited number of bacterial strains, and molecular mechanisms of inhibition remain to be elucidated. Future exploration will include molecular-level exploration of efflux mechanisms to better understand drug–pump interactions, alongside the screening and development of novel EPI candidates. Moreover, in vivo validation studies will be essential to confirm the clinical relevance and therapeutic applicability of such inhibitors.

## Supporting information

S1 File Supporting information. (DOCX)

S1 Fig Graphical abstract. (TIF)
