# Supplementary material for: Schisandrins as novel efflux pumps inhibitors and non-antibiotic compounds against multi- and extensively-drug resistant clinical strains of Salmonella typhi: An in-vitro study
Source: PLoS One. 2026 Jul 31;21(7):e0347214. doi: 10.1371/journal.pone.0347214 (PMC13426931; doi:10.1371/journal.pone.0347214)
Supplement: S1 File — (DOCX) [file pone.0347214.s001.docx]

10.6084/m9.figshare.31920006

<https://doi.org/10.6084/m9.figshare.31920006>
