# Supplementary material for: Schisandrins as novel efflux pumps inhibitors and non-antibiotic compounds against multi- and extensively-drug resistant clinical strains of Salmonella typhi: An in-vitro study
Source: PLoS One. 2026 Jul 31;21(7):e0347214. doi: 10.1371/journal.pone.0347214 (PMC13426931; doi:10.1371/journal.pone.0347214)
Supplement: Attachment — Submitted filename: Reviewer comments- PONE-D-25-32847_reviewer.pdf [file pone.0347214.s003.pdf]

PLOS ONE

Schisandrins as Novel Efflux Pumps Inhibitors and Non-Antibiotic Compounds Against Multi- and Extensively-Drug Resistant Clinical strains of Salmonella typhi: An In-Vitro Study

--Manuscript Draft--

**Manuscript Number:**	PONE-D-25-32847
**Article Type:**	Research Article
**Full Title:**	Schisandrins as Novel Efflux Pumps Inhibitors and Non-Antibiotic Compounds Against Multi- and Extensively-Drug Resistant Clinical strains of Salmonella typhi: An In-Vitro Study
**Short Title:**	Schisandra chinensis, an untapped reservoir of lignans against typhoid fever resistance
**Corresponding Author:**	Nosheen Fatima Rana<br>National University of Sciences and Technology School of Mechanical and Manufacturing Engineering<br>Islamabad, PAKISTAN
**Keywords:**	multidrug-resistant bacteria.; efflux pump inhibitors; efflux activity assays.; biofilm.; Salmonella typhi
**Abstract:**	Purpose<br><br>Salmonella typhi is a significant global health challenge due to its high pathogenicity and antimicrobial resistance. It can switch its lifestyles between biofilm and planktonic phase which allows it to evade host defenses and develop resistance to antibiotics. Salmonella sp. harbors numerous efflux pump-encoding genes involved in efflux pumps overexpression that led to multi-drug resistance (MDR) and extensive drug-resistance (XDR). To overcome the battle against resistant S. typhi strains, novel non-antibiotics inhibitors are required for inhibitory application. This study assesses the inhibitory effect of lignans against drug resistance of S. typhi.<br><br>Methods<br><br>Clinical resistant and sensitive strains of S. typhi were isolated and characterized. The inhibitory effect of lignans, specifically Schisandrin A and B, purified from the plant Schisandra chinensis, as effective non-antibiotic inhibitors were evaluated through standard microbiological techniques like growth curve and time-kill assays. Bacterial morphology studies were conducted using scanning electron microscopy (SEM). Our study explores two approaches, such as efflux pumps (EPs) inhibition and biofilm inhibition assays.<br><br>Results<br><br>Using colony-forming unit (CFU) assays, growth curve analysis, and SEM imaging, we observed significant bacteriostatic effects, with Schisandrin B causing notable membrane disruption. Schisandrin B also showed remarkable biofilm inhibition (90.33%) and strong efflux pumps inhibition.<br><br>Conclusion<br><br>This study offers a strong basis for future research on addressing antibiotic resistance in clinically relevant pathogens.
**Order of Authors:**	Iqra Shafique
	Nosheen Fatima Rana
	Naseer Ahmad
	Tahreem Tanweer

Powered by Editorial Manager® and ProduXion Manager® from Aries Systems Corporation

	Sabah Javaid
	Fawzyah Obeedallah Albaldi
	Serag Eldin I Elbehairi
	Mohammad Y. Alfaifi Elbehairi
	Ali A. Shati
	Farid Menaa
**Additional Information:**	
**Question**	**Response**
**Financial Disclosure**<br><br>Enter a financial disclosure statement that describes the sources of funding for the work included in this submission. Review the submission guidelines for detailed requirements. View published research articles from *PLOS ONE* for specific examples.<br><br>This statement is required for submission and **will appear in the published article** if the submission is accepted. Please make sure it is accurate.<br><br>**Funded studies**<br>Enter a statement with the following details:<br>• Initials of the authors who received each award<br>• Grant numbers awarded to each author<br>• The full name of each funder<br>• URL of each funder website<br>• Did the sponsors or funders play any role in the study design, data collection and analysis, decision to publish, or preparation of the manuscript?<br><br>Did you receive funding for this work?	Yes
Please add funding details.<br>as follow-up to "**Financial Disclosure**<br><br>Enter a financial disclosure statement that describes the sources of funding for the work included in this submission. Review the submission guidelines for detailed requirements. View published research articles from *PLOS ONE* for specific examples.	This research was funded through large Groups (Project under grant number R.G.P. 2/344/44), Deanship of Scientific Research and Graduate Studies at King Khalid University, Saudi Arabia.

This statement is required for submission and **will appear in the published article** if the submission is accepted. Please make sure it is accurate.<br><br>**Funded studies**<br>Enter a statement with the following details:<br>• Initials of the authors who received each award<br>• Grant numbers awarded to each author<br>• The full name of each funder<br>• URL of each funder website<br>• Did the sponsors or funders play any role in the study design, data collection and analysis, decision to publish, or preparation of the manuscript?<br><br>Did you receive funding for this work?"	
Please select the country of your main research funder (please select carefully as in some cases this is used in fee calculation).<br>as follow-up to "**Financial Disclosure**<br><br>Enter a financial disclosure statement that describes the sources of funding for the work included in this submission. Review the submission guidelines for detailed requirements. View published research articles from *PLOS ONE* for specific examples.<br><br>This statement is required for submission and **will appear in the published article** if the submission is accepted. Please make sure it is accurate.	SAUDI ARABIA - SA

**Funded studies**<br>Enter a statement with the following details:<br>• Initials of the authors who received each award<br>• Grant numbers awarded to each author<br>• The full name of each funder<br>• URL of each funder website<br>• Did the sponsors or funders play any role in the study design, data collection and analysis, decision to publish, or preparation of the manuscript?<br><br>Did you receive funding for this work?"	
**Competing Interests**<br><br>Use the instructions below to enter a competing interest statement for this submission. On behalf of all authors, disclose any competing interests that could be perceived to bias this work—acknowledging all financial support and any other relevant financial or non-financial competing interests.<br><br>This statement is required for submission and **will appear in the published article** if the submission is accepted. Please make sure it is accurate and that any funding sources listed in your Funding Information later in the submission form are also declared in your Financial Disclosure statement.<br><br>View published research articles from *PLOS ONE* for specific examples.	The authors declare that they have no known competing financial interests or personal relationships that could have appeared to influence the work reported in this paper.

Powered by Editorial Manager® and ProduXion Manager® from Aries Systems Corporation

**NO authors have competing interests**<br><br>Enter: *The authors have declared that no competing interests exist.*<br><br>**Authors with competing interests**<br><br>Enter competing interest details beginning with this statement:<br><br>*I have read the journal's policy and the authors of this manuscript have the following competing interests: [insert competing interests here]*<br><br>* typeset	
**Ethics Statement**<br><br>Enter an ethics statement for this submission. This statement is required if the study involved:<br><br>• Human participants<br>• Human specimens or tissue<br>• Vertebrate animals or cephalopods<br>• Vertebrate embryos or tissues<br>• Field research<br><br>Write "N/A" if the submission does not require an ethics statement.<br><br>General guidance is provided below. Consult the submission guidelines for detailed instructions. **Make sure that all information entered here is included in the Methods section of the manuscript.**	Written Informed consent was obtained from all participants prior to the collection of blood samples for the detection of Salmonella Typhi." NUST Institutional Review Ethics Committee approved the study. (IRB #: 24-IRB-A-27/27 on May 29th, 2024)

Powered by Editorial Manager® and ProduXion Manager® from Aries Systems Corporation

**Format for specific study types**<br><br>**Human Subject Research (involving human participants and/or tissue)**<br>• Give the name of the institutional review board or ethics committee that approved the study<br>• Include the approval number and/or a statement indicating approval of this research<br>• Indicate the form of consent obtained (written/oral) or the reason that consent was not obtained (e.g. the data were analyzed anonymously)<br><br>**Animal Research (involving vertebrate animals, embryos or tissues)**<br>• Provide the name of the Institutional Animal Care and Use Committee (IACUC) or other relevant ethics board that reviewed the study protocol, and indicate whether they approved this research or granted a formal waiver of ethical approval<br>• Include an approval number if one was obtained<br>• If the study involved *non-human primates*, add *additional details* about animal welfare and steps taken to ameliorate suffering<br>• If anesthesia, euthanasia, or any kind of animal sacrifice is part of the study, include briefly which substances and/or methods were applied<br><br>**Field Research**<br><br>Include the following details if this study involves the collection of plant, animal, or other materials from a natural setting:<br>• Field permit number<br>• Name of the institution or relevant body that granted permission	
**Data Availability**<br><br>Authors are required to make all data underlying the findings described fully available, without restriction, and from the time of publication. PLOS allows rare exceptions to address legal and ethical concerns. See the PLOS Data Policy and FAQ for detailed information.	Yes - all data are fully available without restriction

A Data Availability Statement describing where the data can be found is required at submission. Your answers to this question constitute the Data Availability Statement and **will be published in the article**, if accepted.<br><br>**Important:** Stating 'data available on request from the author' is not sufficient. If your data are only available upon request, select 'No' for the first question and explain your exceptional situation in the text box.<br><br>Do the authors confirm that all data underlying the findings described in their manuscript are fully available without restriction?	
**Describe where the data may be found in full sentences. If you are copying our sample text, replace any instances of XXX with the appropriate details.**<br><br>• If the data are **held or will be held in a public repository**, include URLs, accession numbers or DOIs. If this information will only be available after acceptance, indicate this by ticking the box below. For example: *All XXX files are available from the XXX database (accession number(s) XXX, XXX.).*<br>• If the data are all contained **within the manuscript and/or Supporting Information files**, enter the following: *All relevant data are within the manuscript and its Supporting Information files.*<br>• If neither of these applies but you are able to provide **details of access elsewhere**, with or without limitations, please do so. For example:<br><br>*Data cannot be shared publicly because of [XXX]. Data are available from the XXX Institutional Data Access / Ethics Committee (contact via XXX) for researchers who meet the criteria for access to confidential data.*<br><br>*The data underlying the results presented in the study are available from (include the name of the third party*	All relevant data are within the manuscript and its Supporting Information files.

and contact information or URL).<br>• This text is appropriate if the data are owned by a third party and authors do not have permission to share the data.<br><br>* typeset	
Additional data availability information:	Tick here if the URLs/accession numbers/DOIs will be available only after acceptance of the manuscript for publication so that we can ensure their inclusion before publication.

Powered by Editorial Manager® and ProduXion Manager® from Aries Systems Corporation

Manuscript
Click here to access/download;Manuscript;Original Manuscript updated 17 june.doc

.

Original article

Schisandrins as Novel Efflux Pumps Inhibitors and Non-Antibiotic Compounds Against Multi- and Extensively-Drug Resistant Clinical strains of *Salmonella typhi*: An *In-Vitro* Study

Running title: *Schisandra chinensis*, an untapped reservoir of lignans against typhoid fever resistance

Iqra Shafique[1], Nosheen Fatima Rana[1*], Naseer Ahmad[2], Tahreem Tanweer[1], Sabah Javaid[1], Fawzyah Obeedallah Albaldi[3], Serag Eldin I[4]. Elbehairi , Mohammad Y. Alfaifi [4], Ali A. Shati [4], and Farid Menaa[5]

[1] Department of Biomedical Engineering and Sciences, School of Mechanical Manufacturing Engineering, National University of Science Technology, Islamabad 44000, Pakistan. ishafique.phd21smme@student.nust.edu.pk (I.S.); nosheen.fatima@smme.nust.edu.pk (N.F.R.); ttanweer.phd19smme@student.nust.edu.pk (T.T.); sabah.javaid@smme.nust.edu.pk (S.J.)

[2] Excel Labs - Blue Area -, Islamabad 44010, Pakistan. naseer@excel-labs.com (N.A.)

[3] Department of Biology, Faculty of Science, Al-Baha University, Alaqiq 65779-7738, Saudi Arabia. falbeladi@bu.edu.sa (F.O.A.)

[4] King Khalid University, Faculty of Science, Biology Department, Abha 9004, Saudi Arabia; serag@kku.edu.sa (S.E.I.E.), alfaifi@kku.edu.sa (M.Y.A.), aaalshati@kku.edu.sa (A.A.S.)

[5] Department of Biomedical and Bioenvironmental Engineering (BEE), California Innovations Corporation (CIC), San Diego 92037, CA, USA; menaateam@gmail.com (F.M.); https://orcid.org/0000-0002-0258-7322

[*] To whom the correspondence shall be addressed.

Graphical Abstract

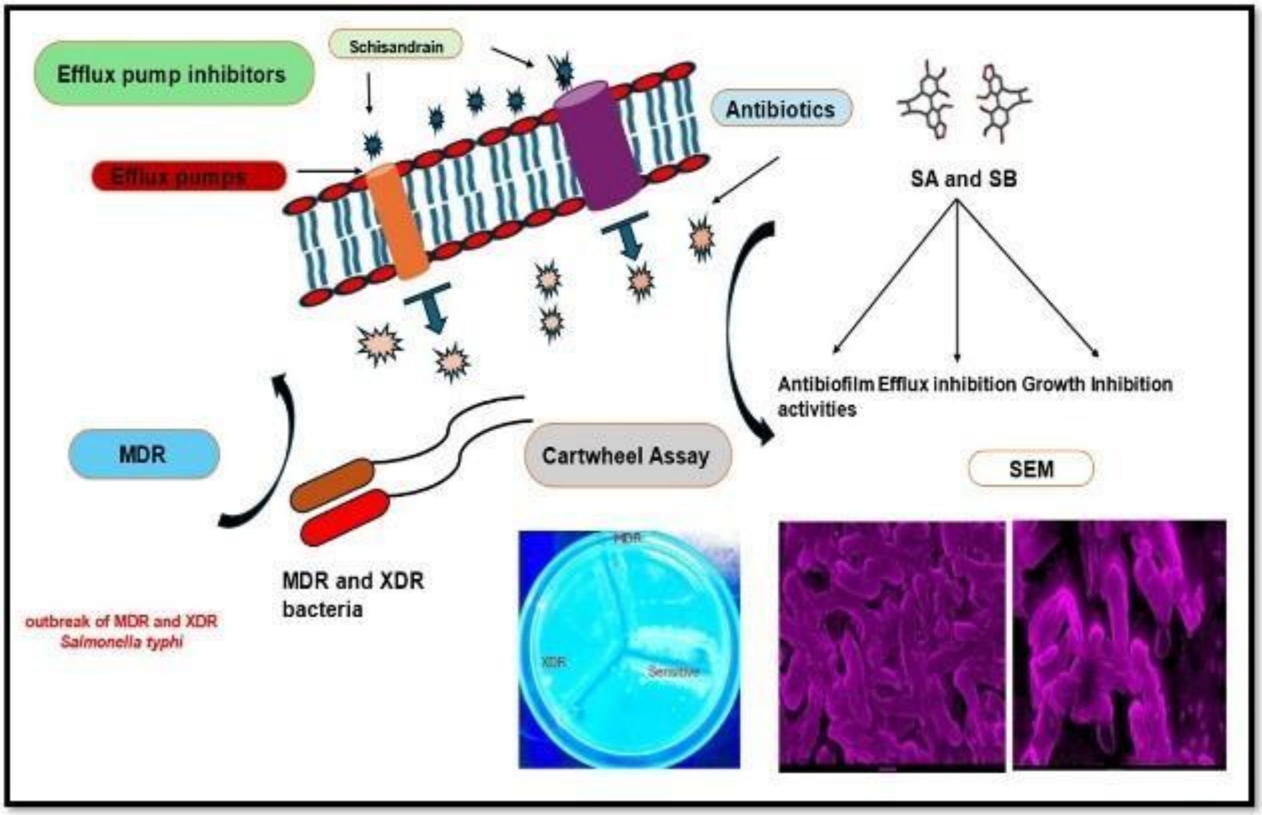

Abstract

Purpose: *Salmonella typhi* is a significant global health challenge due to its high pathogenicity and antimicrobial resistance. It can switch its lifestyles between biofilm and planktonic phase which allows it to evade host defenses and develop resistance to antibiotics. *Salmonella* sp. harbors numerous efflux pump-encoding genes involved in efflux pumps overexpression that led to multi-drug resistance (MDR) and extensive drug-resistance (XDR). To overcome the battle against resistant *S. typhi* strains, novel non-antibiotics inhibitors are required for inhibitory application. This study assesses the inhibitory effect of lignans against drug resistance of *S. typhi*. **Methods:** Clinical resistant and sensitive strains of *S. typhi* were isolated and characterized. The inhibitory effect of lignans, specifically Schisandrin A and B, purified from the plant *Schisandra chinensis*, as effective non-antibiotic inhibitors were evaluated through standard microbiological techniques like growth curve and time-kill assays. Bacterial

morphology studies were conducted using scanning electron microscopy (SEM). Our study explores two approaches, such as efflux pumps (EPs) inhibition and biofilm inhibition assays. **Results:** Using colony-forming unit (CFU) assays, growth curve analysis, and SEM imaging, we observed significant bacteriostatic effects, with Schisandrin B causing notable membrane disruption. Schisandrin B also showed remarkable biofilm inhibition (90.33%) and strong efflux pumps inhibition. **Conclusion:** This study offers a strong basis for future research on addressing antibiotic resistance in clinically relevant pathogens.

Keywords: multidrug-resistant bacteria., efflux pump inhibitors, efflux activity assays., biofilm., *Salmonella typhi.*

1. Introduction

Typhoid is an infectious disease predominantly caused by *Salmonella enterica* serovar *typhi* (1). It infects humans and is acquired through contaminated food or water consumption. Common clinical manifestations involve elevated body temperature, nausea, abdominal pain, and [illegible]gular bowel movements (2, 3). *S. typhi* is accountable for approximately 76.3% of typhoid cases. Globally, typhoid is estimated to range involving 11 to 27 million cases annually resulting in 128,000 to 161,000 deaths. In 2017, Global Health Data Exchange (GHDx) reported around 116,800 typhoid related deaths, with approximately 79,000 of these occurring in South Asia, including 6,700 in Pakistan (4, 5).

Despite major advances in medicine, a considerable global population remains susceptible to typhoid fever (6). In developing nations, antibiotic regimens are commonly deployed for typhoid management. However, its widespread use introduces the risk of adverse effects which promotes the emergence of antimicrobial resistant strains (7). Since 2016, Pakistan has faced the outbreak of typhoid, driven [illegible] an XDR st[illegible], resulting in the emergence of a MDR H58 strain (6). In the recent years, studies revealed high *S. typhi* infection rate in the country, with approximately 493.5 cases per 100,000 population and thousands of cases of XDR typhoid reported in Sindh, a southeastern region of Pakistan, and in Lahore, a northeastern region of Pakistan (8-11[illegible]

MDR bacterial pathogens are known to resist lethal doses of antibiotics drugs through a mechanism involving multidrug EPs (12). It is worth noting that XDR bacterial pathogens even more problematic, since they are resistant to almost all the approved antimicrobial agents,

leaving very few therapeutic options (13-15). This may be explained by the fact that, in addition to increased efflux of drug by EPs, pathogenic bacteria can employ diverse other mechanisms of resistance including alteration of targets and inactivation of drugs.

In Pakistan, the rising occurrence of typhoid is attributed to MDR and XDR *S. typhi* is concerned about the efficacy of antibiotic interventions (16). Eps, predominantly recognized in bacteria for conferring MDR (17), play a vital role in antimicrobial resistance by expelling antimicrobial drugs from bacteria cell, by lowering the intracellular accumulation of toxic antibiotic levels, and conferring non-susceptibility in gram-negative pathogens (18). Beyond drug extrusion, bacterial EPs are also involved in maintaining cellular physiological activities, including pathogenicity, virulence, cell-to-cell communication and biofilm formation (19). Indeed, the over expressive EPs influence the formation of biofilm to promote the initial microbial adherence, extracellular matrix (ECM) production, transport of metabolites, quorum sensing (QS), and biofilm-associated genes indirectly (22). Prokaryotic organisms exhibit five superfamilies of multi-drug EPs, contributing to the onset of MDR (20, 21). Notably, the ATP binding cassette (ABC) transporters emerge as crucial proteins within plasma membrane organelles. They are prevalent across all living cells and intermittently in Gram-negative bacteria (22). Thus, efflux pump inhibitors (EPIs) serve as a promising solution to overcome MDR, as well as bacterial virulence and biofilm formation (23).

Phytocompound-based EPIs play an important role in inhibiting drug efflux and increase lethality at lower dosages (24). On the other hand, they are able to disrupt biofilm formation by blocking the transport of QS-signal molecules (25). Some research groups have reported the efficacy of natural compounds, such as lignans, flavonoids, alkaloids, to inhibit outer membrane protein in MDR cells lines. Naturally occurring lignans, such as Schisandrin compounds, originating from the family of *Schisandra chinensis*, significantly inhibit the P-glycoprotein (P-gp), an ABC transporter family member also encountered in various drug-resistant cancer cell lines (26). Reported lignans such as Schisandrin (SA) and Schisandrin (SB) were shown to elicit anti-inflammatory, anticancer, antioxidant, as well as antibacterial activities (31-34). Although their anti-inflammatory, antioxidant and anticancer activities have been explored, there is still a gap in understanding their antibiofilm, inhibitory activities and inhibitory mechanisms against typhoid specifically *S. typhi*. In our previous study, SA and SB were predicted as effective inhibitors against EPs, i.e. ABC-TPA, in *S. typhi* through *in silico* studies (27). Keeping these

results in view, and for further validation of our previous results, SA and SB were explored for their role as EPIs against clinical isolates of *S. typhi*. Based on previous results [35], we hypothesized that SA and SB can effectively inhibit EPs in MDR and XDR *S. typhi* strains.

The present study was conducted to validate the capacity of SA and SB as novel non-antibiotic compounds to enhance the treatment efficacy against *S. typhi*. For this purpose, our main objectives were two folds: (i) isolating and identifying MDR and XDR strains, and (ii) utilizing the SA and SB against these strains to evaluate the inhibitory effects against *S. typhi* EPs.

2. Methodology

All the chemicals were purchased from Sigma Aldrich (Hamburg, Germany). Prior to the commencement of the study, ethical approval was secured from the institutional review board (IRB #: 24-IRB-A-27/27 on May 29th, 2024) of the National University of Sciences and technology (NUST), Islamabad, Pakistan.

2.1 Sample collection

Blood samples were collected from patients suspected of having typhoid fever over a 10-day period, from July 1 to July 10, 2024 and identified as *Salmonella typhi* with a specific emphasis on assessing drug resistance profiles using Kirby-Bauer method. Ethylenediaminetetra-acetic acid (EDTA) tubes were utilized to collect blood samples and tubes were stored at 4°C.

2.2. Isolation and culturing

The blood samples were collected in blood culture bottles containing growth-supportive media. The inoculated bottles were incubated in a Vitek 2 Compact system, which monitored bacterial growth according to the manufacturer's guidelines. Upon detecting positive cultures, a subculture was performed by transferring drops of the culture onto MacConkey agar plates and left for overnight incubation at 37°C. Non-lactose fermented colonies were isolated, and the oxidase test was then performed and those that tested negative were confirmed to be motile, gram-negative rods (28).

2.3. Biochemical testing

The isolates were further identified through the Analytical Profile Index (API 10E) system, which provided the biochemical profile of identified *S. typhi*. Colonies were characterized

according to their morphological characteristics subsequently undergoing a comprehensive analysis of biochemical evaluations utilizing the API 10E system (bioMerieux, France) for detailed identification. The biochemical evaluations were performed to determine the specific metabolic and enzymatic features including the oxidase test, arabinose fermentation, ornithine decarboxylase (ODC) test, indole formation (IND), citrate utilization, glucose fermentation (GLU) testing, tryptophan deaminase (TDA) assessment, urease (URE) function lysine decarboxylase (LDC) activity, hydrogen sulfide (H_2S) production and the nitrophenyl-b-D-galactopyranoside (ONPG) reaction. The colonies were incubated for a duration of 18 to 24 hours at 37°C to facilitate observable biochemical reaction thereby enhancing the precise identification and characterization of the bacterial isolates (9).

2.4. Kirby Bauer method

Antibiotic susceptibility and resistance were identified by utilizing the Kirby Bauer disk diffusion method. Isolated colonies were cultivated on tryptic soy agar for 24 hours of incubation at 37°C. Diluted suspensions of *S. typhi* isolates matching with McFarland 0.5 turbidity standards. The bacterial suspensions were applied uniformly on TSA and allowed to adhere for a period of 10 minutes. Subsequently antibiotic discs from Oxoid (Thermo Fisher Scientific) were then positioned on the TSA plates placed at incubator at 37°C for 24 hours. (7, 29).

The antibiotics tested included ampicillin (10 µg), chloramphenicol (30 µg), ciprofloxacin (5 µg), sulfamethoxazole/trimethoprim (SXT, 25 µg), imipenem (10 µg per disk), meropenem (10 µg), azithromycin (15 µg), and ceftriaxone (30 µ g) per disk. Each disc was placed on the surface of TSA separately and incubated overnight at 37°C. Zone of inhibition were measured (30)

2.5. Ethidium bromide (EtBr)-agar cartwheel (EtBrCW) assay

The EtBrCW assay was utilized to evaluate the efflux pump activity based on accumulation of EtBr (fluorescent probe) within the bacterial cells. The clinical bacterial isolates including MDR, XDR and control (sensitive isolate) were cultured at 37°C with agitation for 24 hours). Tryptic soy agar (TSA) medium with 2.5 µg/mL EtBr was prepared and bacterial isolates were swabbed onto agar plates before their incubation for 16-18 hours at 37°C. The plates were visualized using UV transilluminator (Biorad, Dubai, UAE) at the wavelength (λ) = 302 nm. Then images of

plates were captured through gel documentation system (Bio-Rad Gel Doc EZ Imager, Dubai, UAE) (31-33).

2.6. Biofilm formation of MDR and XDR *S. typhi* isolates

2.6.1. Air-liquid interface assay

This qualitative assay (34, 35) was utilized to evaluate the biofilm-forming potential of the MDR and XDR *S. typhi* isolates. In this assay, cultures of isolates were inoculated in tryptic soy broth (TSB) in each well of the 12-well plates. Coverslips were placed at 90° angle in the incubator for 48 hours at 37°C. For this, during a period of this incubation, after the biofilm adhered to the coverslip surface, the coverslips were lifted carefully in a vertical orientation utilizing sterilized forceps, while minimizing any disturbance to the biofilm layer that has been formed. Subsequently, the coverslips underwent a gentle washing process with distilled water by employing a method of gradual dipping and lifting for each coverslip. The coverslips were then placed within another 12-well plate for staining with 0.1% crystal violet solution. This procedure was conducted for 15 minutes at room temperature (RT), permitting sufficient time for the crystal violet to effectively bind to the biofilm without any form of agitation. The distilled water was used to clean the coverslips. High-power light microscope (Zeiss, Oberkochen, Germany) was used to visualize the coverslips (34, 35).

2.6.2. Spectrometric analysis of biofilm formation

In this quantitative assay (34, 35), bacterial cultures were adjusted to attain the 0.5 McFarland index which corresponds to approximately 1.5×10^8 CFU/mL. The bacterial cultures were inoculated in TSB in 96-well plate and incubated overnight for 24 hours. A well with media served as negative control. After incubation, the planktonic (free-floating) cells were gently removed and washed thrice with phosphate-buffered saline (PBS) before adding 0.1% crystal violet solution to each well and incubated for 10-15 minutes at RT to stain the biofilm. After incubation, the wells were carefully washed thrice with PBS to remove excess stain, leaving only the stained biofilm attached to the well surfaces. Then, 95% ethanol was added to each well to solubilize the crystal violet bound to the biofilm, and the optical density (OD) at λ= 630 nm was measured. The biofilm formation was scored according to the OD value and following interpretation: (0.0-0.5) meant weak biofilm, (0.5) signified moderate biofilm, and (> 0.5) indicated strong biofilm (34, 35).

2.7. Evaluation of intrinsic antimicrobial activity of Schisandrins A (SA) and B (SB)

2.7.1. Efflux pump inhibition (EPI) assay

Bacterial inoculum was dispensed into wells of a sterile black 96-well microtiter plate. SA and SB (16) were loaded into columns 1-4, while 100 µM EtBr was added to every well. Dimethylsulfoxide (DMSO) (control) was added in column 5. The fluorescence of accumulated EtBr was then examined using a fluorescence spectroscopy (Fl6500, Perkin USA)at λ= 530 nm and λ= 600 nm (36).

2.7.2. *Determination of minimum inhibitory concentration*

The MICs of SA and SB against MDR and XDR strains were determined using broth dilution method. A stock solution (1 mg/mL) of SA and SB was serially diluted in test tubes to achieve concentrations of 0.5, 1, 3, 5[illegible], 20, and 40 µg/mL. Each solution was inoculated with 50 µl of bacteria. TSB used as control was also inoculated with bacterial isolates. The plate was placed in the incubator for 6 hours at 37°C and after that visually examining the turbidity under good lighting with visible cloudiness indicates the bacterial growth proliferation. After 6 hours of incubation the lowest concentration at which maximum growth inhibition occurred was considered as the MIC (45, 46).

2.7.3. *Growth curve analysis*

The growth patterns of S. typhi strains were examined after exposure to SA and SB to quantify their intrinsic antibacterial efficacy. After the 4 hours of culturing of overnight MDR and XDR S. typhi cultures at 37°C the cultures were then suspended and SA and SB were added at concentrations of (0.5, 1, 3, 5, 10, 20, 40 µg/mL). Subsequent incubation at 37°C in an orbital shaker incubator at 160 rpm. Control cultures of MDR and XDR without additives were included and [12] at 600 nm was measured at hourly intervals for up to 8 hours. Graphical representations were performed using GraphPad Prism 9 (47, 48).

2.7.4. *Colony forming unit (CFU) analysis by time kill assay*

Time-kill analysis was conducted using a previously reported method [45, 49, 50]. First, S. typhi strains were sub-cultured and diluted. Then, different concentrations of 3, 5, 10, and 20 µg/mL of SA and SB was added in to sterile TSB, along with bacterial inoculum. The samples

were placed in the incubator for 6 hours at 37°C. After that the collected aliquots were inoculated onto TSA and incubated at 37°C for overnight. CFU was then calculated. Finally, a log cfu/mL versus time graph was plotted using graph pad prims to analyze bacterial growth dynamics with data derived from the growth curve assay to assess microbial proliferation over time (45, 49, 50).

2.7.5. Effect of Schisandrins on morphology by scanning electron microscopy (SEM)

The morphology of untreated and treated MDR or XDR *S. typhi* isolates were subjected to SEM imaging (35, 37). Isolates were inoculated into TSB and incubated for 12 hours (overnight). SA and SB were then introduced at a final concentration of (2 × MIC) for 6 hours. Control samples were left untreated. After centrifugation of the samples, 3% glutaraldehyde (GA) was used to fix the sediment cells, which were dehydrated with a series of ethanol concentrations and then dried to a critical point. The dried cells were coated with gold and bacterial cell morphologies were observed using a VEGA3 LMU SEM apparatus (Tescan, Brno, Czech Republic) (35, 37).

2.7.6. Antibiofilm activity of the Schisandrin compounds

The anti-biofilm properties of SA and SB were evaluated through a microtiter plate spectroscopic assay (35, 38, 39). Fresh cultures of isolates were cultured in TSB overnight followed by dilution in fresh TSB. The control well contained DMSO solvent at a concentration equivalent to that of treated with the compounds. The microplate was then incubated for 24 hours at 37°C. The liquid (medium or DMSO) was removed carefully without disturbing the biofilm attached to the well surface. Wells were gently rinsed followed by staining with crystal violet solution (0.1%) for 20 minutes. Following staining, the wells underwent another round of washing and were air dried. Afterward, 95% ethanol was added to the wells and and solution was incubated for 15 minutes at room temperature. After that, (150 µl) of solution was gently transferred to a separate 96-well plate. The OD was then calculated at 630 nm using a microplate reader. The biofilm inhibition was measured using the following formula (equation1):

$$\frac{(ODc-ODb)-(ODt-ODb)}{(ODc-ODb)} \times 100\%$$

Where, ODc represents the absorbance of non-treated isolates, ODb stands for absorbance of TSB (culture medium), and ODt is the absorbance of treated isolates.

3. Results

3.1. Isolation and identification of *S. typhi* isolates

Blood samples were obtained from patients suspected of having typhoid at Excel Lab Islamabad Pakistan. The samples were cultured on MacConkey agar placed in incubator at 37°C for 24 hours. Samples showed growth in the MacConkey agar media indicating the presence of bacteria.

3.2. Biochemical identification of the *S. typhi* isolates

The biochemical identification of *S. typhi* isolates as identified through the API 10E system (bioMerieux, France), provides a clear distinction from other enteric bacteria and is crucial for accurate pathogen identification. The negative reactions for oxidase, ARA, ODC, TDA, IND, URE, and citrate utilization indicate that these isolates lack certain enzymes and metabolic pathways, differentiating them from organisms like *Proteus* (urease-positive), *Pseudomonas* (oxidase-positive), and *Klebsiella* (citrate-positive). In contrast, the positive results for glucose (GLU) fermentation, lysine decarboxylase (LDC) activity, and hydrogen sulfide (H_2S) production are indicative markers of *S. typhi*, reflecting its ability to produce acid from glucose metabolism to decarboxylate lysine into cadaverine, and to produce hydrogen sulfide, which creates a black precipitate in culture media. Additionally, the isolates' inability to hydrolyze ONPG, shown by a negative ONPG reaction, confirms the absence of β-galactosidase consistent with *S. typhi*'s known trait as a non-lactose fermenter. These results indicate a specific metabolic profile that not only verifies the identity of *S. typhi* but also aids in distinguishing it from similar organisms (Fig.1).

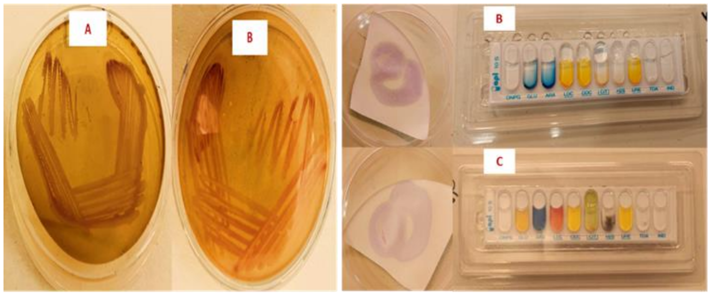

Biochemical Test	*Salmonella typhi* Isolates	Biochemical Test	*Salmonella typhi* Isolates
IND	-	ODC	-
ONGP	-	CIT	-
GLU	+	H2S	+
ARA	-	URE	-
LDC	+	TDA	-

Figure 1. Isolation and biochemical identification of *Salmonella typhi* isolates through API 10S (A) Selective plating on MacConkey agar, (B) Oxidase Test before the Incubation and API 10S Before the incubation, (C) Oxidase test become negative and API 10S after the incubation.

3.3. Kirby Bauer method

Eight antibiotics from different generations were used to assess antibiotic resistance levels in *S. typhi* isolates. Out of the four tested isolates, one isolate was identified as (MDR), displaying resistance to chloramphenicol, sulfamethoxazole/trimethoprim, ampicillin, and ciprofloxacin. Another isolate was classified as XDR due to its resistance to the aforementioned antibiotics, including Cefixime. Two isolates of *S. typhi* were found to be sensitive, exhibiting susceptibility to all tested antibiotics. The resistance profiles of *S. typhi* isolates to the nine different antibiotics are summarized in (Fig. 2).

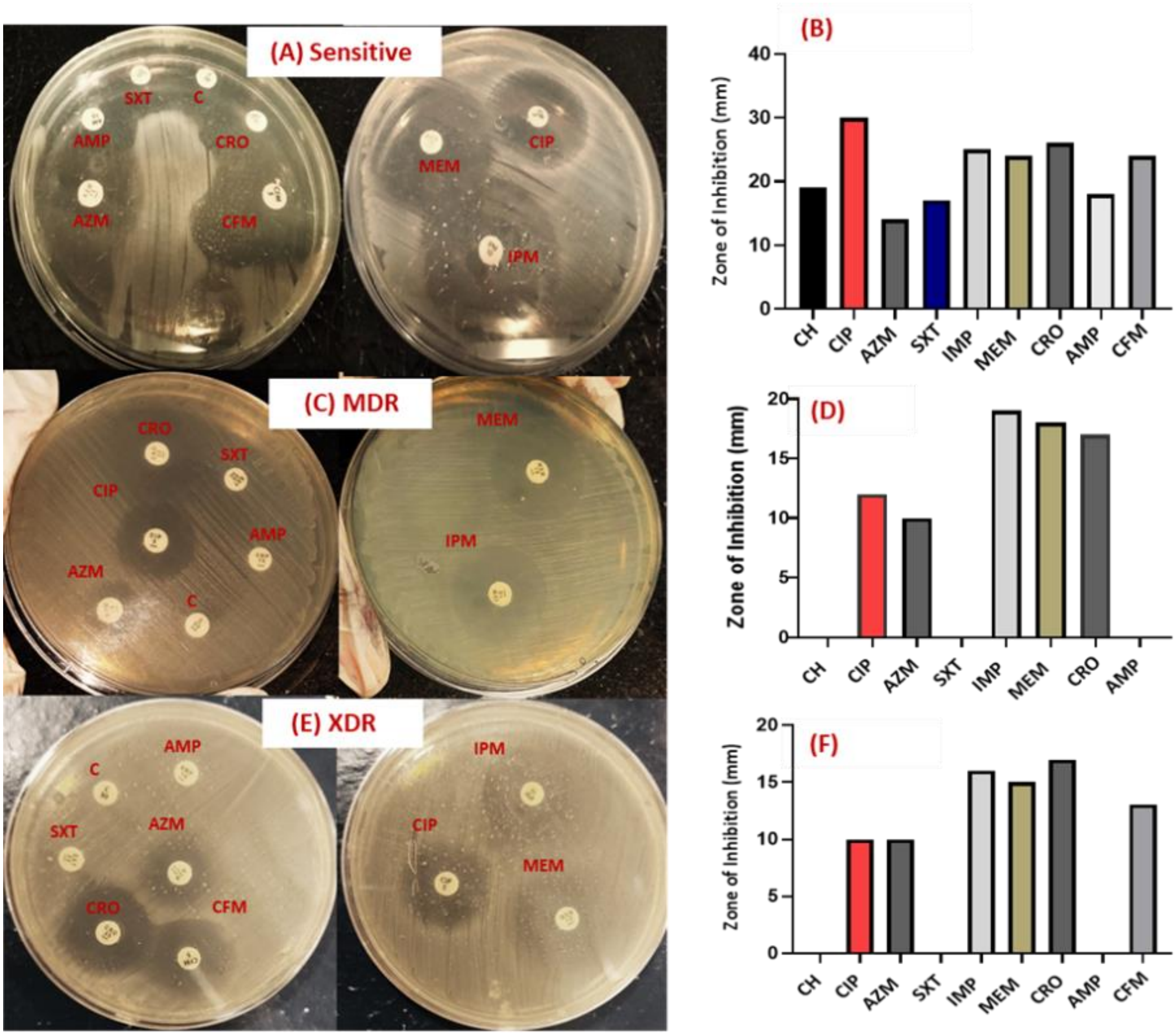

Figure 2: Identification of drug resistance through disk diffusion Assay and bar graph analysis of *S. typhi* isolates (A) and (B) Sensitive strain, (C) and (D) MDR strain, and (E) and (F) XDR strain

3.4. Detection of efflux pump through ETBr-agar cartwheel (EtBrCW) assay

The EtBrCW assay was utilized to detect the efflux pump activity in *S. typhi* strains by directly visualizing the fluorescence based on EtBr accumulation (Fig. 1). The intensity of fluorescence was lowly detectable in MDR (Fig. 3A) and undetectable in XDR (Fig. 3B) strains, whereas it was high in the sensitive strain of *S. typhi* used as control (Fig. 3C).

These data indicate actively expressed efflux pumps in the test isolates. Also, the fluorescence observed in control indicates that the strain is unable to efficiently expel EtBr, leading to its accumulation within the bacterial cells. Indeed, accumulated EtBr binds to DNA which caused an increase in fluorescence intensity because EtBr is an intercalating agent that inserts itself between DNA base pairs causing it to fluoresce more brightly upon binding.

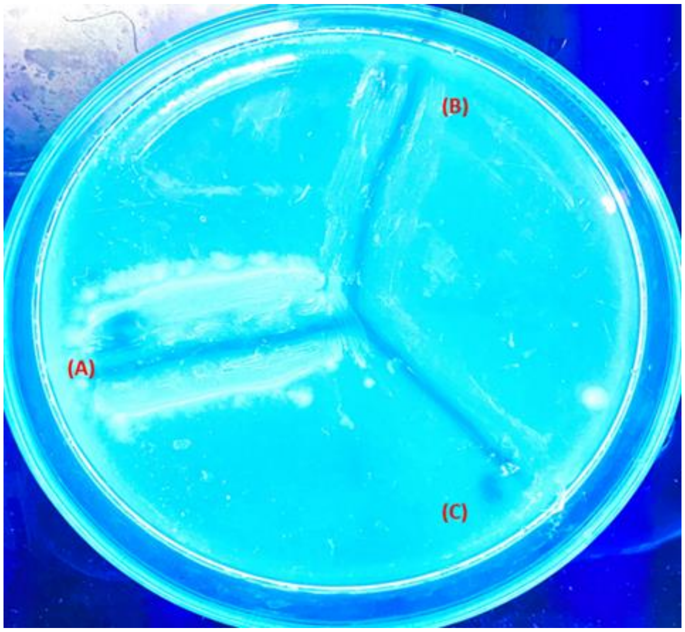

Figure 3. EtBr-agar cartwheel (EtBrCW) assay of isolated *Salmonella typhi* strains (A) in multi-drug resistant (MDR), (B) in extensive drug-resistance (XDR) strain, and (C) in sensitive strain (control).

3.5. EtBr efflux pump inhibition (EPI) assay

We have adapted an existing fluorescence assay using EtBr to monitor overtime accumulation (efflux inhibition) activities of *S. typhi* strains connected to lignans SA/SB resistance/tolerance (Fig. 4). EtBr acts as a substrate of efflux pumps and it g[illegible]xpelled from the cells when EPs are actively overexpressed. Conversely, when the EPs are inhibited then EtBr is accumulated within the cells. The efflux of EtBr is presented in terms of fluorescence intensity, which is obtained for the MDR (Fig. 4A), XDR (Fig. 4B), and the sensitive strains (Fig. 4C), in the presence (100 µM)

or absence (control) of lignans (SA or SB) (31) The fluorescence intensity at the (600 nm) emission and (530 nm) excitation provided a reliable measure of the potential efflux of EtBr from the bacterial cells.

Before exposition to lignans, the weakly increased fluorescence intensity overtime observed in the test strains is similar to that of the fluorescence intensity observed in the sensitive strain, indicating that the EtBr is slightly accumulated within the bacterial cells within 30 min.

After exposition [illegible] SA or SB, significant increases (P < 0.05) in fluorescence intensity are observed overtime in MDR and XDR strains compared to that of the sensitive strain. SA exerted a better effect (P < 0.05) in MDR compared to that of XDR, while SB elicited a similar effect in both resistant strains. The effect of SB was higher (P < 0.05) compared to that of SA, when evaluated in both MDR and XDR strains. SA or SB did not display any significant effect (P > 0.05) in the sensitive strain, which contribute to understand mechanistically drug-sensitiveness of this strain.

Indeed, at 3 minutes, both SA and SB started with similar EtBr fluorescence intensities around 25,000, independently of the strain. However, by 10 minutes, the EtBr fluorescence intensity has reached around 34298.6 for MDR and 31791.9 for XDR strains treated with SA (P < 0.05), whereas the EtBr fluorescence intensity has risen to approximately 43918.7 for MDR and 43883.9 for XDR strains treated with SB (P > 0.05). By 30 minutes, SA showed a more moderate increase (about 3800 for MDR, and 3200 for XDR) compared to SB (about 50000 for MDR and XDR) (P < 0.05).

Taken together, since SA or SB had no effect in the sensitive *S. typhi* strain contrarily to resistant *S. typhi* strains, we could confirm that *S. typhi* MDR and XDR strains use EPs as a mean to resist to chemical treatments; importantly, the lignans SA, and particularly SB, are potential EPIs to overcome that resistance. The lower fluorescence intensity and EtBr accumulation in the sensitive strain suggest that EPs are present, but they are less active than in the MDR and XDR strains.

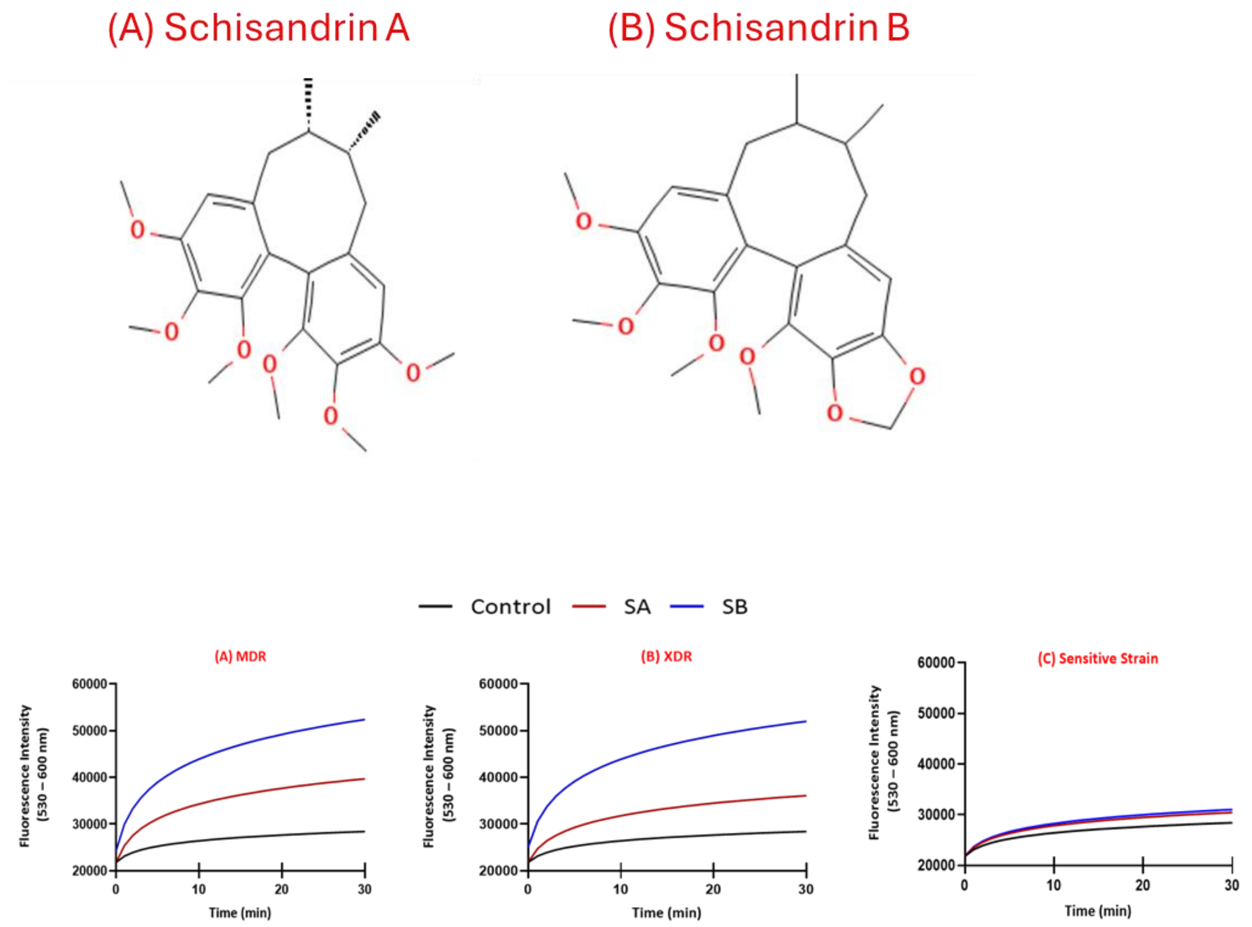

Figure 4. SA and SB structure and efflux pump inhibition (EPI) effects of Schisandrin A (SA) and Schisandrin B (SB) against MDR, XDR and sensitive *Salmonella typhi* isolates. (A) in multi-drug resistant (MDR) strain, (B) in extensive drug-resistance (XDR) strain, and (C) in sensitive strain (control). SA and SB were used at the same concentration (100 µM).

3.6. Biofilm-formation potential of *S. typhi* isolates

The biofilm formation results indicated that the *S. typhi* isolates, either resistant and sensitive, have a strong potential for biofilm formation as it was evidenced by coverslip assay, from which staining and visible aggregation of bac[illegible]ial colonies are observed (Figs. 5A-C). The quantitative assay showed significant biofilm formation with OD 630 nm values above 0.5 (Fig. 5 D).

Actually, the MDR and XDR isolates showed a significantly higher biofilm-forming potential with a mean value of 1.0 and 1.2, respectively, when compared to that of the sensitive isolate

(0.8), used as control ($p = 0.003$). There was also a significant difference ($P < 0.05$) in biofilm formation when test isolates are compared to each other.

These results suggested that the enhanced biofilm formation in the XDR isolate may contribute to its increased resistance. Resistant isolate often demonstrates altered cell surface properties such as enhanced production of EPs, which are known to be important components for biofilm formation and stability.

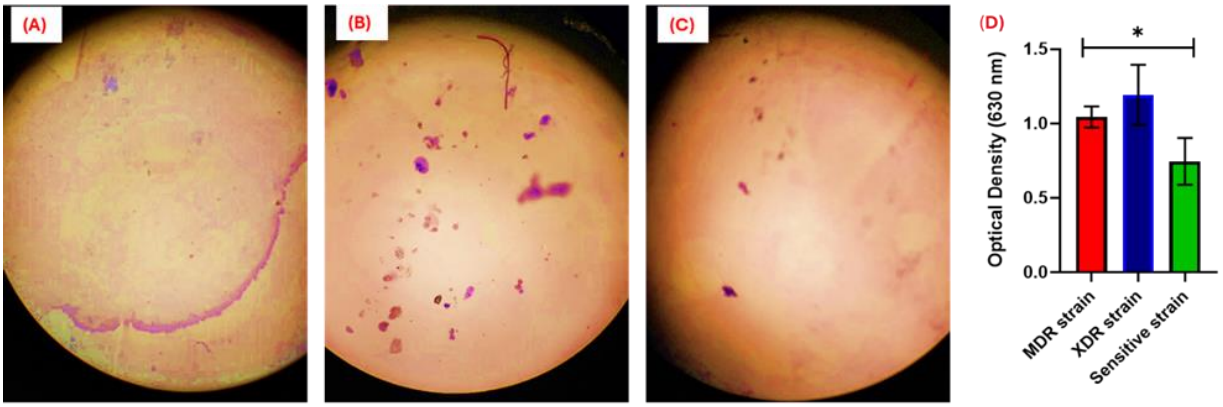

Figure 5. Qualitative and quantitative biofilm formation potential of *Salmonella typhi* isolates. Cover slip assay of (A) in multi-drug resistant (MDR) strain, (B) in extensive drug-resistance (XDR) strain, and (C) in a sensitive strain (control), and (D) spectrophotometric analysis.

3.7. Minimum inhibitory concentration analysis of Schisandrin A and B against *S. typhi* isolates

The growth inhibition effec[illegible] SA and SB against MDR and XDR *S. typhi* was analyzed to evaluate the effect of SA and SB against the MDR and XDR S. typhi through different microbiological assays. The MIC analysis showed an inhibition of 45–50 % of growth at a range of 10–20 µg/mL demonstrated the highest inhibitory effect of SA and SB against MDR and XDR *S. typhi*.

.

3.8. Time kill assay

The time kill analysis was utilized to ensure the efficacy of SA and SB against bacterial CFU counts. It showed a decrease in CFU of the bacteria with an increase in concentration of SA and SB for MDR isolate (Fig. 6A and D) , XDR S. typhi isolate (Fig. 6B and E) and sensitive isolate (Fig. 6C and F). Drug SA exhibits effectiveness against both MDR, XDR and sensitive isolates. For MDR bacteria, the mean CFU / ml values decrease from 9.045 (SD = 0.07750) to 3 µg/ml to 8.540 (SD = 0.000) at 20 µg/mL, with (p-value = 0.0027). For XDR bacteria, the mean values decrease from 8.961 (SD = 0.08556) at 5 µg/ml to 8.045 (SD = 0.06364) at 20 µg/ml, showing a similar pattern (p-value = 0.0135). For Sensitive isolate the mean values decrease from 8.03 (SD = 0.4879) to 3 µg/ml to 7.34 (SD = 0.1485) at 20 µg/ml, with (p-value = 0.0072). These results suggested that increasing the concentration of Drug SA beyond a certain point does not significantly enhance its antibacterial effect as indicated by the statistically significant p-values. Drug SB shows a gradual decline against MDR, XDR and sensitive isolates as the concentration increases. For MDR bacteria, the mean CFU/ml values decrease from 9.045 (SD = 0.07750) at 3 µg/ml to 8.085 (SD = 0.1202) at 20 µg/ml (p-value = 0.0088). A similar trend was observed against XDR bacteria with a statistically significant p-value of 0.0032. The mean CFU/ml values drop from 9.045 (SD = 0.07750) to 3 µg/ml to 8.535 (SD = 0.09192) at 20 µg/ml. For sensitive isolate the mean value decreases from 8.625 (SD =0.1061) at 3 µg/ml to 8.370 (SD = 0.098_ with (p-value = 0.0497).

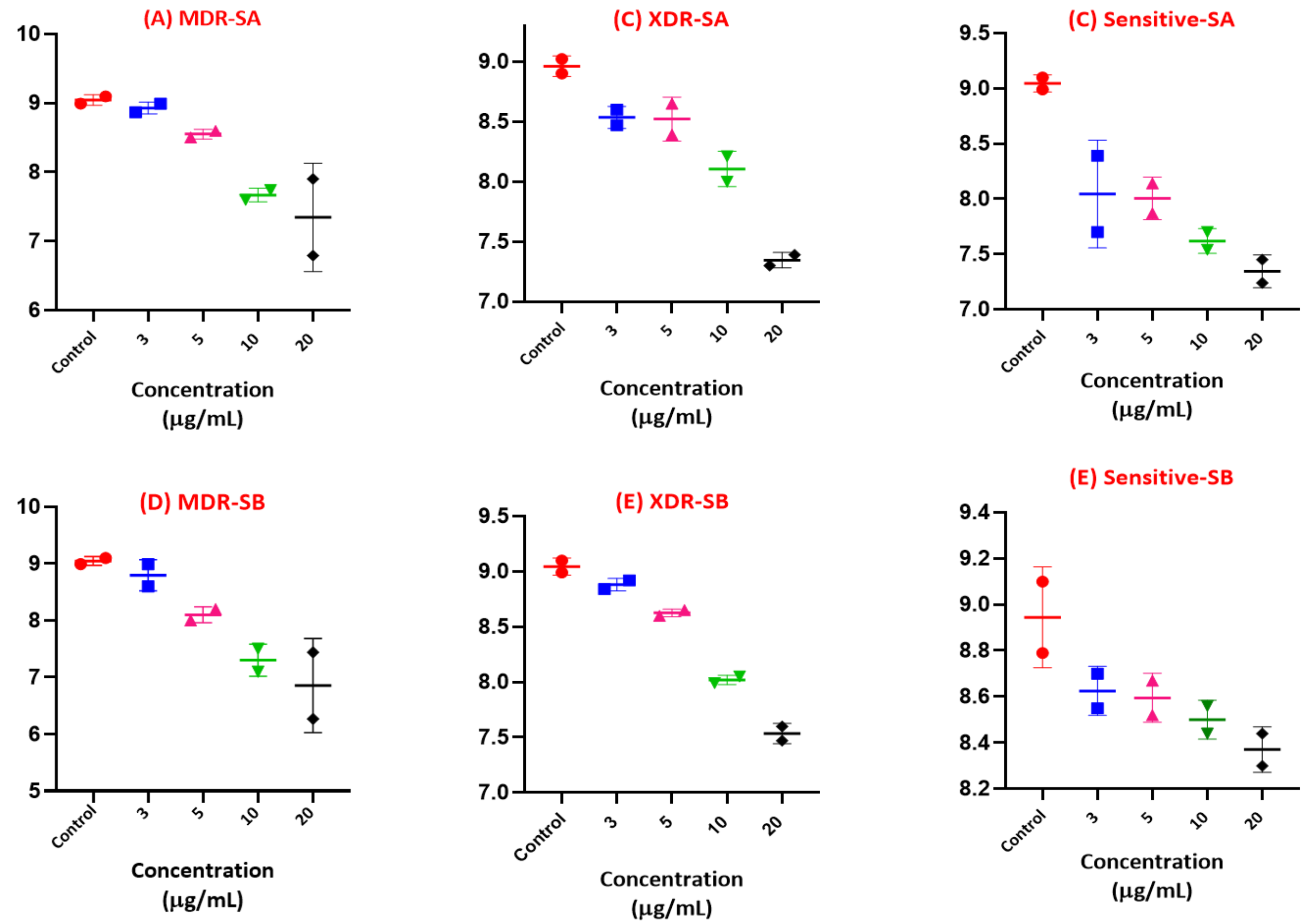

Figure 6: CFU analysis of MDR and XDR S. typhi by Time kill assay after 6 hours. (A) CFU analysis of MDR S. typhi with SA (B) CFU analysis of MDR S. typhi with SB, (C) CFU analysis of XDR S. typhi with (D) CFU analysis of XDR S. typhi with SB.

3.9. Growth curve analysis

The growth curve analysis showed that the increase in concentration of SA and SB the bacterial growth curve was found to be decreased over time, as shown in (Fig 7). The growth curve was found to be equivalent of control at lowest concentration of 1 µg/ml. These results indicate that as the concentration of SA and SB increased from 5 µg/mL to 20 µg/mL, there was a noticeable and consistent inhibition of bacterial growth. This suggests that SA and SB have a bacteriostatic effect, meaning they can inhibit the growth and reproduction of bacteria without necessarily killing them. These findings highlight the SA and SB as potential non antibiotic inhibitors,

particularly in controlling bacterial growth. The observed decline in bacterial growth after 6 hours suggests that the compounds exerted their bacteriostatic over time.

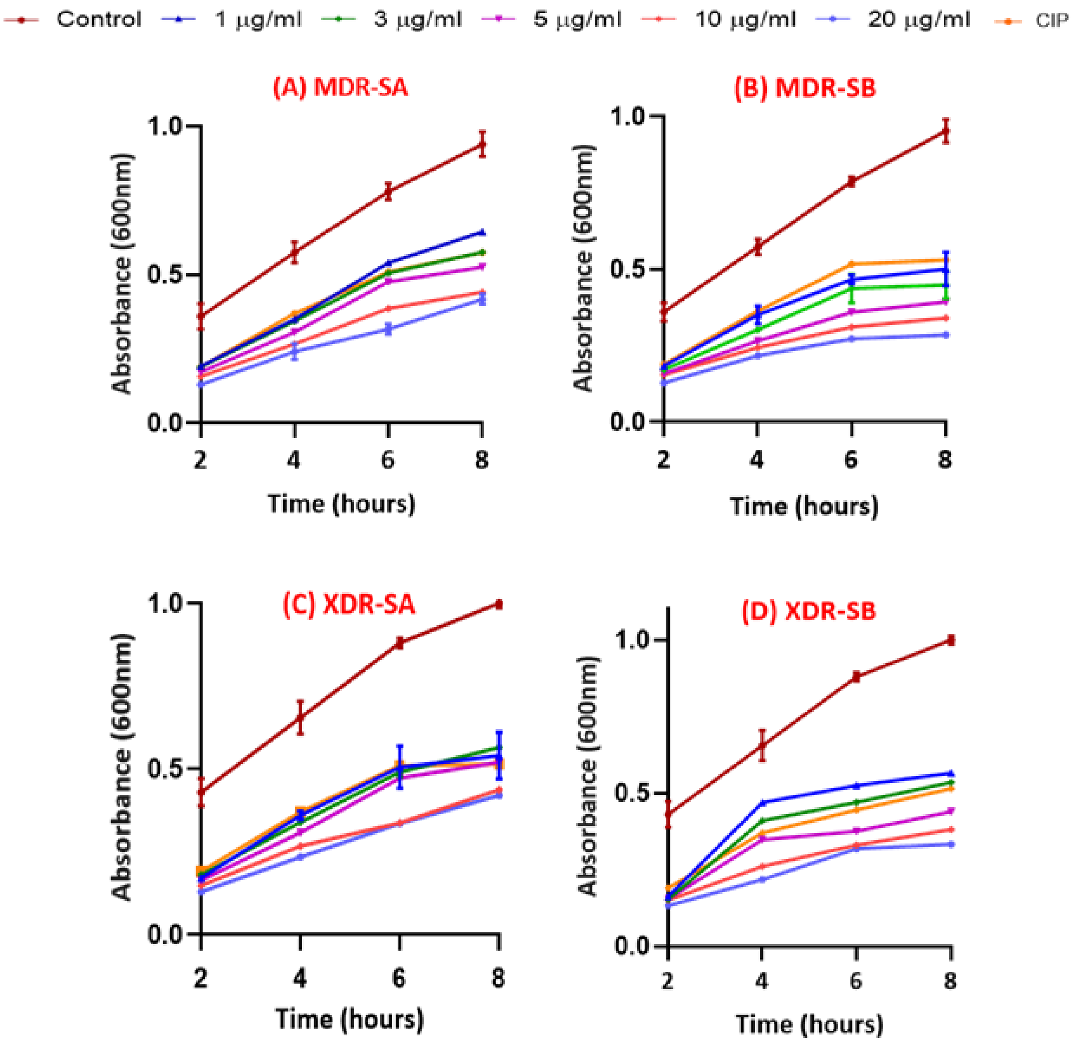

Figure 7: Growth effect of SA and SB against S. typhi strains (A) Growth effect of MDR and XDR S. typhi with SA (B) Growth curve of MDR and XDR S. typhi with SB.

3.10. Impact of Schisandrin compounds on the morphology of *S. typhi* cells

SEM analyses were performed on MDR, XRD and sensitive strains of of *S. typhi* to visualize whether SA or SB has an impact on their cell morphology (Fig. 8). Importantly, both SA and SB had notable effects on their morphology, but their impact differed significantly.

S. typhi cells that were treated with SA at 2 × MIC for 6 hours caused visible damage to the bacterial cells. This suggests that SA has a moderate effect on cell morphology (Fig. 8B , E and H). In contrast, SB treatment under the same conditions resulted in severe damage to the bacterial cells. The cells exhibited more pronounced changes, including wrinkled and withered surfaces, along with membrane distortion and the disappearance of the outermost layer (Fig. 8 C ,F and I) In the absence of SA and SB *S. typhi* cells were intact with regular surfaces and outer layers were intact (Fig. 8A, D and G).

These findings imply that SB is more potent than SA in damaging the external structures of *S. typhi* cells.

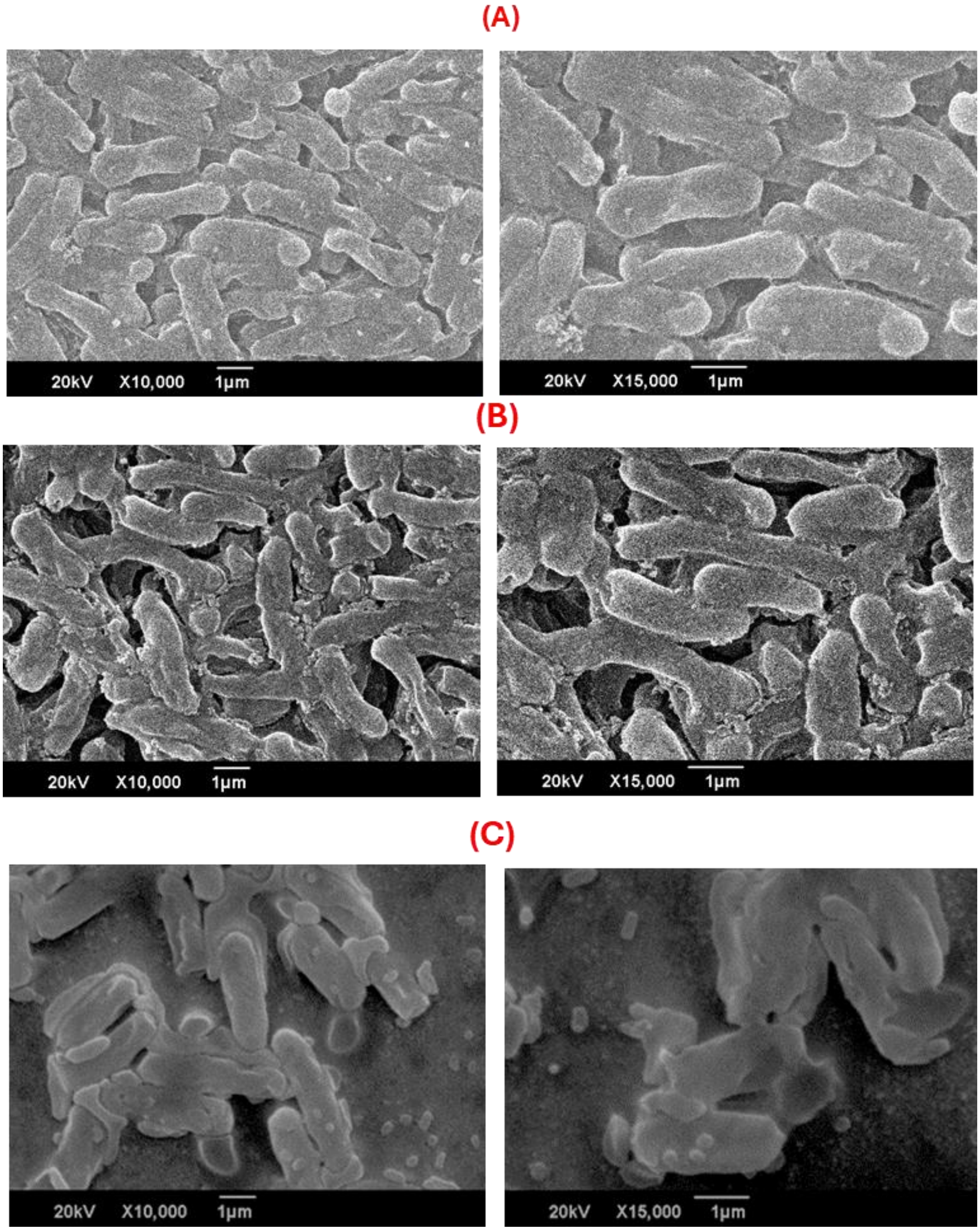
(A)
20kV X10,000 1μm
20kV X15,000 1μm
(B)
20kV X10,000 1μm
20kV X15,000 1μm
(C)
20kV X10,000 1μm
20kV X15,000 1μm

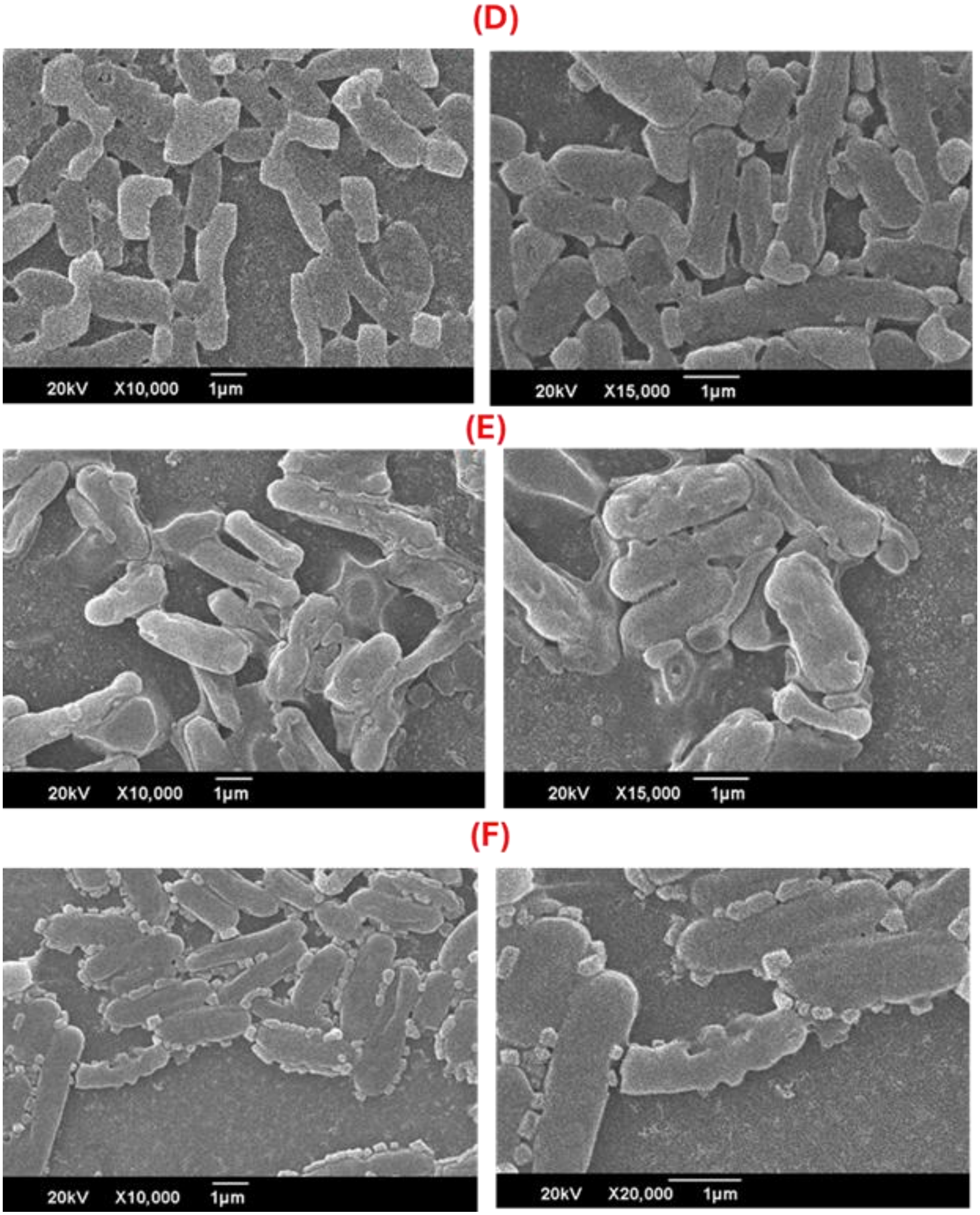
(D)
20kV X10,000 1μm
20kV X15,000 1μm
(E)
20kV X10,000 1μm
20kV X15,000 1μm
(F)
20kV X10,000 1μm
20kV X20,000 1μm

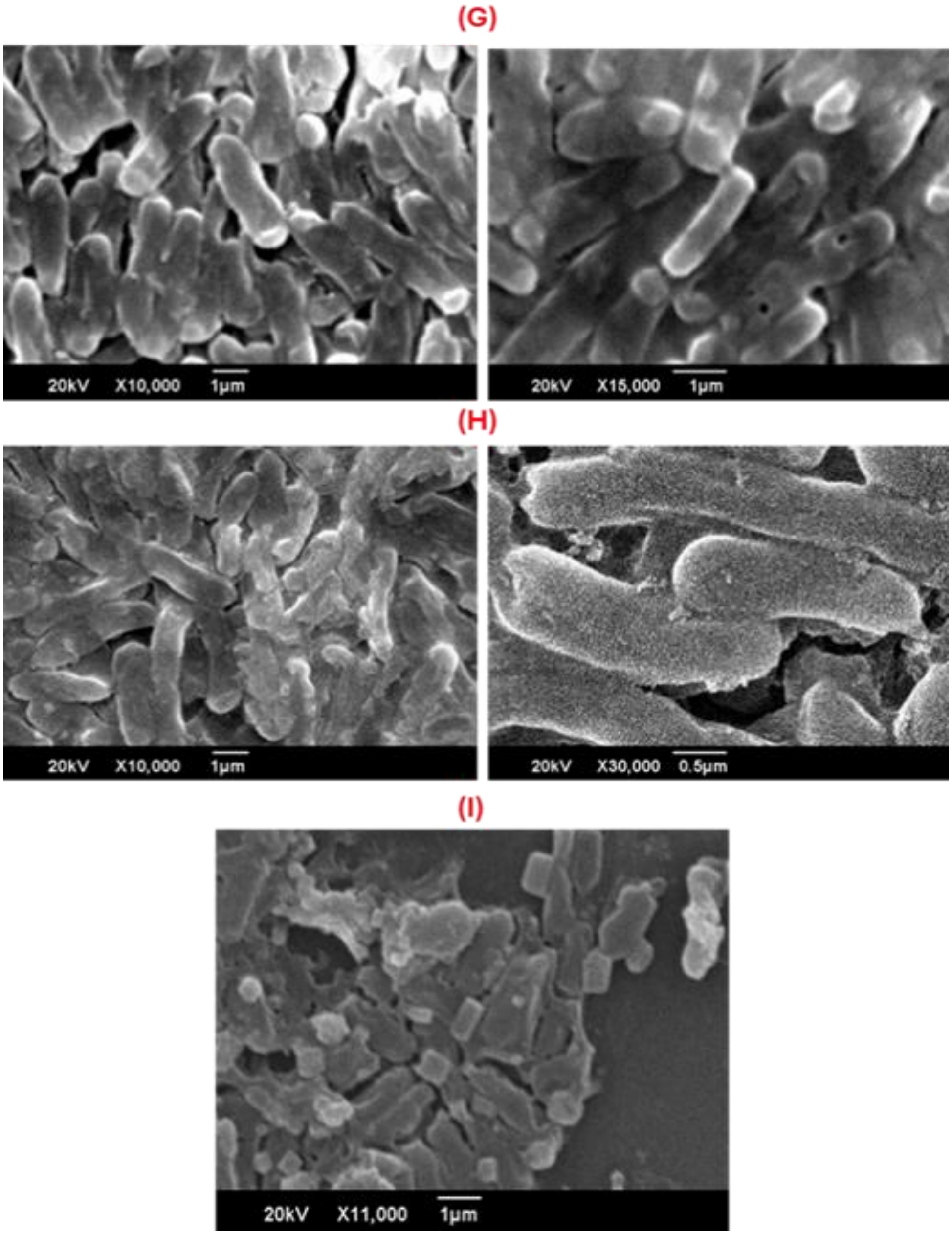

Figure 8. Scanning electron microscopy (SEM) images of *Salmonella typhi* isolates. (A-C) in multi-drug resistant (MDR) strain, (D-F) in extensive drug-resistance (XDR) strain, and (G-I) in sensitive strain, with: (A, D, G) untreated (control), (B, E, H) treated with Schisandrin A (SA), (C, F, I) treated with Schisandrin B (SB). SA and SB were used at 2 X MIC for 6 hours. Magnifications and scale bars are indicated.

3.11. Antibiofilm activity of SA and SB against MDR and XDR *S. typhi* isolates

The quantitative biofilm assay was used to evaluate the antibiofilm potential of SA and SB, at various concentrations (i.e., 0, 3, 5, 10, 20, 40 µg/mL) against MDR and XDR *S. typhi* (Fig. 9). Both drugs demonstrated significant inhibition of biofilm formation. SA showed mean inhibition percentages of 88.00% for MDR (Fig. 9A), 85.00% for XDR (Fig. 9B) and (Fig. 9C) at 20-40 µg/mL, while SB exhibited slightly higher inhibition with 90.33% for MDR (Fig. 9D) and 89.33% for XDR (Fig. 9E) and 87% for sensitive isolate at the same concentration. The P-values of < 0.0001 for both drugs, compared to the control (untreated) suggest the strong efficacy of SA and SB with SB showing a marginally higher effectiveness ($P > 0.005$) in biofilm inhibition compared to that of SA.

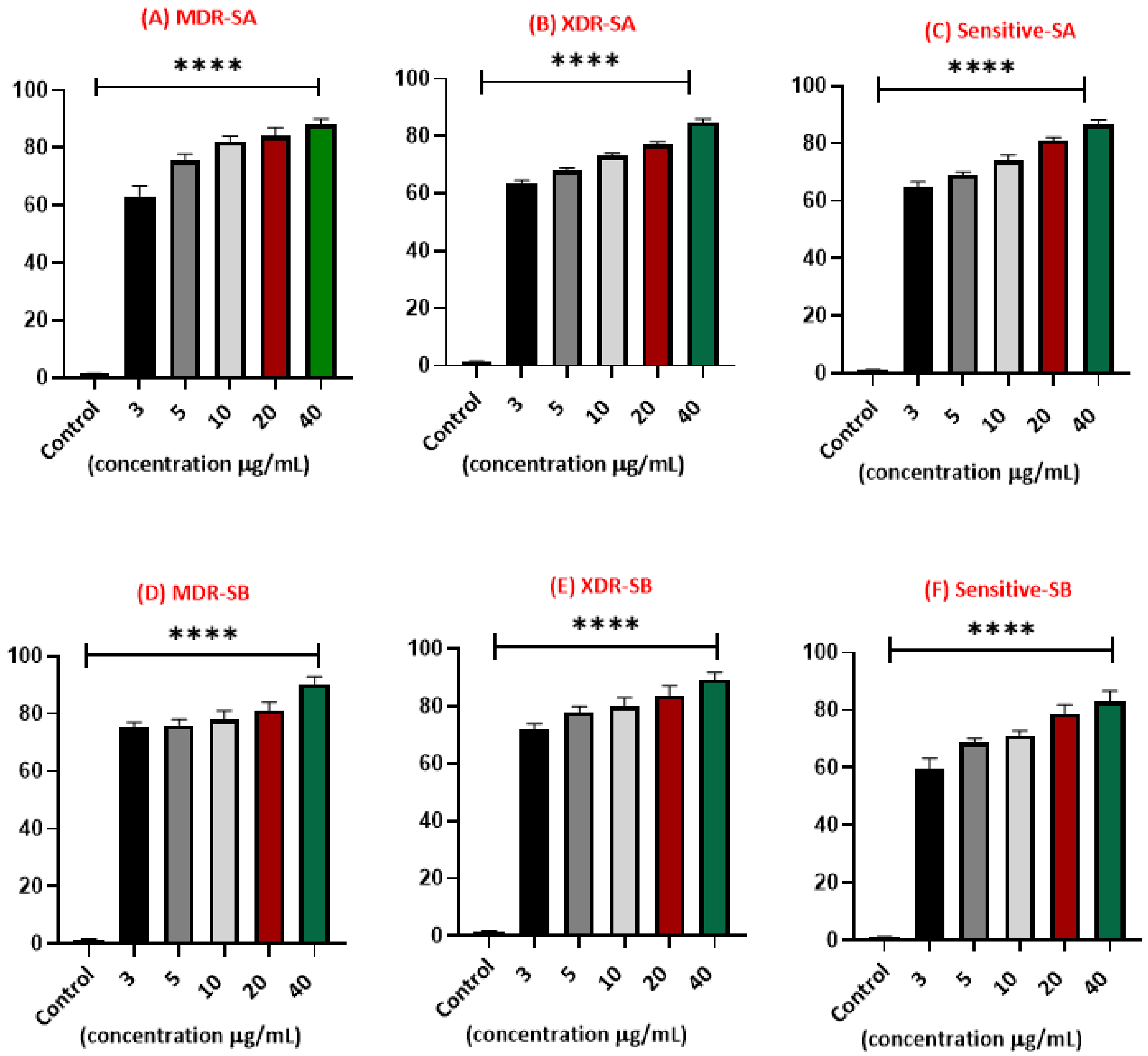

Figure 9. Antibiofilm activity (%) of Schisandrin A (SA) and Schisandrin B (SB) toward multidrug-resistance (MDR) and extensive drug-resistance (XDR) *Salmonella typhi* isolates. (A) SA against MDR *S. typhi*, (B) SB against MDR *S. typhi*, (B) SA against XDR *S. typhi*, (D) SB toward XDR *S. typhi*.

4. Discussion

Despite the effectiveness of several antimicrobials against typhoid caused by *S. typhi*, the development of drug-resistant *S. typhi* strains complicates treatment (7).

.

In this study, we report the isolation and identification of clinical resistant *S. typhi* strains, using selective plating and biochemical analysis with a specific emphasis on efflux pump (EP) mechanisms. Such approach is necessary for advancing our understanding and overcoming the antimicrobial resistance (6, 40).

Briefly, we employed the Kirby-Bauer disc diffusion method (DDM) and eight antibiotics of different generations to assess the antimicrobial resistance (AMR) of the *S. typhi* isolates. The results revealed that high levels of resistance within the *S. typhi* isolates. Specifically, one isolate demonstrated MDR against chloramphenicol, sulfamethoxazole/trimethoprim, ampicillin, and ciprofloxacin. Another isolate exhibited XDR, showing resistance not only to the aforementioned antibiotics but also to Cefixime, thus severely limiting treatment options. Similar results for antibiotic resistance against *S. typhi* have been reported in previous studies (28, 41-45).

The successful isolation and identification of resistant strains provided a foundation for EP detection through EtBr-agar cartwheel assay. This assay is based on EtBr accumulation by directly visualizing fluorescence to assess EP activity (46, 47). Our findings revealed that the sensitive strain (control) showed the highest fluorescence due to (EtBr) accumulation resulting from low EP activity. In contrast, the MDR and XDR strains exhibited the lowest fluorescence levels indicating high EP activity and overexpression. Similar findings using the same assay have been reported in previous studies (33, 48).

Our subsequent investigations focused on assessing the EP inhibition (EPI) activity of lignans SA and SB based on our previous *in-silico* analysis of their activity (27) We comprehensively tested these lignans (Schisandrins) for their ability to inhibit EP using the EtBr accumulation assay. Our results clearly demonstrated EPI activities of both SA and SB on EtBr in MDR, XDR, and sensitive strains of *S. typhi*. SB was the most effective EPI inhibitor against *S. typhi* isolates as indicated by enhanced fluorescence intensity and EtBr accumulation in resistant isolates compared to the sensitive isolate. Indeed, SB more effectively led to enhanced EtBr accumulation inside bacterial cells compared to SA. The reduced fluorescence intensity and EtBr accumulation in the sensitive isolate suggested that EPIs are present, but they are less active as compared to MDR and XDR isolates. Our results were supported by a previously reported assay.

Moreover, we examined the biofilm forming capacity of *S. typhi* isolates which are frequently correlated with their AMR and virulence. Several studies have reported that the biofilm formation is associated with altered surface properties of cells in resistant isolates involving

increased production of EPs. (33, 49, 50). An antibiofilm effect was clearly observed when increasing SA and SB concentrations which was associated with a decrease in CFU. SB exhibited stronger antibiofilm activity compared to that of SA. To understand whether this antibiofilm activity was due to bacteriostatic effect (reversible bacterial growth inhibition) or by bactericidal effect (irreversible bacterial growth inhibition/killing) effect, SEM analyses were performed to eye-naked visualize potential damages of the cell membrane of bacteria treated with SA and SB. The bacterial cell structures of the control (untreated) were intact when compared to that of lignans-treated strains, in which membrane damages and shrinkages were noticed. Further, the time-kill assay indicated a decline in bacterial growth after 6 hours, confirming the lignans-mediated bactericidal effect. SB showed a stronger antibacterial effect. Our findings emphasize the importance of SA and SB to overcome the MDR and XDR S. *typhi* infections through irreversible inhibition of bacterial growth.

Taken together, these *in-vitro* data demonstrated a dual function of the lignans SA and SB in the resistance and control of biofilm formation of (MDR and XDR) S. *typhi* strains. Indeed, the EPI effect of the lignans (SA and SB) was revealed through a mechanism involving drug retention into bacterial cytoplasm and DNA, and their ability of acting as antibiotics-like compounds was depicted through a mechanism involving the disruption of the bacterial cell membrane integrity. Similar findings were reported (51, 52), including by the use of *S. chinensis* extract [65], from where SA and SB are originated. Although we previously showed that SA and SB could be effective inhibitors against EPs, i.e. ABC-TPA, in *S. typhi* (27), the present study confirms our hypothesis, and report for the first time the importance of these lignans to naturally overcome *S. typhi* resistance.

Recent studies have expanded the understanding of the antibacterial properties of *S. chinensis*, which have been reviewed recently(53). Thereby, *S. chinensis* extracts were efficient in both inhibiting the growth of methicillin-resistant *Staphylococcus aureus* (MRSA) but also in disrupting its biofilm formation, a critical factor in MRSA's resistance and persistence(53). Moreover, the antibacterial activity of *S. chinensis* extracts against *Helicobacter pylori*, a bacterium associated with gastric ulcers and cancer, was prominent in inhibiting the growth of *H. pylori* and reducing the expression of virulence factors. In addition to whole extracts, recent notable studies are focusing on isolating specific compounds responsible for the antibacterial activity of *S. chinensis*. Purification of some of these compounds identified tartaric acid as a

significant active compound against *Streptococcus mutans*, a primary bacterium responsible for dental caries(54). In accordance with our present study, Liu et al., 2022, identified gomisin A and schisandrin B as potent antibacterial agents against various Gram-positive and Gram-negative bacteria, through a mechanism implicating the disruption of bacterial cell membranes, leading to cell lysis and death(55). Studies have also investigated the potential synergistic effects of *S. chinensis* extracts with conventional antibiotics. A study by Park et al., 2023, examined the combination of *S. chinensis* extract with ampicillin and found a synergistic effect against multidrug-resistant *Escherichia coli*. This combination therapy significantly reduced bacterial load compared to the antibiotic alone, suggesting that *S. chinensis* could enhance the efficacy of existing antibiotics and help combat antibiotic resistance(53). Clinical trials are being conducted to assess the effectiveness of *S. chinensis* extracts in treating infections in humans. Preliminary results from a trial by Lee et al. 2023, indicated that the topical application of *S. chinensis* extract could effectively reduce symptoms of bacterial skin infections, highlighting its potential as a natural antimicrobial agent for dermatological use(53).

Although lignans, a sub-group of non-flavonoid polyphenols(56), are known to exert pleiotropic biological activities including potent antibacterial effects(57), there is still a paucity of reports in this regard. Also, to the best of our knowledge, no studies have deeply characterized SA or SB compounds, and no one of them have been explored to fight resistant strains of *S. typhi*. These overall promising findings against resistant clinical isolates of *S. typhi* underscore the importance of lignans SA and SB and *S. chinensis* extracts which should led to further explorations of its potential clinical applications.

4. Limitations of the study

Further explorations shall include (i) the potential additive or synergistic effects of SA and/or SB with conventional antibiotics, at pre-determined concentrations and time; (ii) the imaging/monitoring of *S. typhoid* strains pre-immobilized in coverslips to study their behavior under different conditions or look at specific protein expression patterns during dynamic processes; (iii) the testing of SA and SB against a wide number of microbial and viral infections; (iv) pre-clinical studies using animal models of the typhoid fever.

5. Conclusion

Herein, our study reveals the EPI and antibacterial activity of Schisandrin A and Schisandrin B. These lignans isolated and purified from *S. chinensis*, a plant widely available in Asia, have been explored for the first time against resistant (MDR and XDR) strains of *S. typhi*, responsible for the life-threatening typhoid fever which caused a recent outbreak in Pakistan. Despite the fact that Pakistan became the first country in the world to introduce the World Health Organization (WHO)-recommended typhoid conjugate vaccine (TCV) into its routine immunization program, it is worth mentioning that this effective solution is a short-term strategy due to its accessibility in low-middle countries (e.g., high cost, cultural and religious barriers toward vaccines) and inadequate practices in some regions (e.g., poor sanitation, contaminated water, overuse of antibiotics). To overcome the limited treatment of the population, and because the AMR leads to the emergence of newer, more resistant strains, it is essential to search for alternative therapeutic solutions. One of them is the constant search of naturally active compounds or phytoextracts exerting effective activity against *S. typhi* resistant strains. This work represents an important step toward the scalable production of SA and SB, which strongly demonstrates potency as EPI and natural antibiotics to overcome *in vitro* biofilm formation (and so, persistence and virulence) of resistant strains of *S. typhi* (both MDR and XDR). These findings highlight lignans, specifically SB, as an effective agent for biofilm disruption and their potential biomedical application as non-antibiotic therapeutic options to combat antibiotic resistant *S. typhi*.

Statements and Declarations

Ethics approval: The ethics committee of the National University of Sciences and technology (NUST), Islamabad, Pakistan, endorsed the experimental protocol # 24-IRB-A-27/27, on May 29th 2024.

Consent for publication: all the authors agreed with the content of this article and agreed with its submission to the journal.

Availability of data and materials: The data that support the findings of this study are available from the corresponding authors upon reasonable request.

Conflicts of interest: There are no conflicts of interest related to this work.

Competing interests: The authors declare that they have no known competing financial interests or personal relationships that could have appeared to influence the work reported in this paper.

Funding: This research was funded through large Groups (Project under grant number R.G.P. 2/344/44), Deanship of Scientific Research and Graduate Studies at King Khalid University, Saudi Arabia.

Acknowledgments: The authors thank the dean of the National University of Sciences and technology (NUST), Islamabad, Pakistan for providing us access to the core lab facilities.

Authors Contributions: *Conceptualization:* **N.F.R., I.S., F.M.;** *Supervision:* **N.F.R., F.M.;** *Investigation:* **I.S., N.F.R., N.A.;** *Methodology:* **N.F.R.; I.S., F.M.;** *Software*: **N.F.R.; I.S.;** *Formal analysis:* **I.S., N.F.R.; F.M.;** *Visualization:* **I.S., N.F.R., S.J, T.T., F.O.A., S.E.I.E., M.Y.A., A.A.S., F.M.;** *Data validation:* **I.S., N.F.R., S.J, T.T., F.O.A., S.E.I.E., M.Y.A., A.A.S., F.M.;** *Data curation*: **I.S., N.F.R., S.J, T.T., F.O.A., S.E.I.E., M.Y.A., A.A.S., F.M.;** *Resources*: **N.F.R., F.M.;** *Funding*: **N.F.R., S.E.I.E., F.M.;** *Writing-original draft preparation:* **I.S., N.F.R., T.T., F.M.;** W*riting-review and editing*: **I.S., N.F.R., S.J, T.T., F.O.A., S.E.I.E., M.Y.A., A.A.S., F.M.;** *Article submission:* **N.F.R.**

All authors have read and agreed to the published version of the manuscript.

References

1. Marks F, von Kalckreuth V, Aaby P, Adu-Sarkodie Y, El Tayeb MA, Ali M, et al. Incidence of invasive salmonella disease in sub-Saharan Africa: a multicentre population-based surveillance study. The Lancet Global Health. 2017;5(3):e310-e23.
2. Shaikh OA, Asghar Z, Aftab RM, Amin S, Shaikh G, Nashwan AJ. Antimicrobial resistant strains of Salmonella typhi: The role of illicit antibiotics sales, misuse, and self-medication practices in Pakistan. Journal of Infection and Public Health. 2023;16(10):1591-7.
3. Park SE, Pham DT, Boinett C, Wong VK, Pak GD, Panzner U, et al. The phylogeography and incidence of multi-drug resistant typhoid fever in sub-Saharan Africa. Nature Communications. 2018;9(1):5094.
4. Stanaway JD, Reiner RC, Blacker BF, Goldberg EM, Khalil IA, Troeger CE, et al. The global burden of typhoid and paratyphoid fevers: a systematic analysis for the Global Burden of Disease Study 2017. The Lancet Infectious Diseases. 2019;19(4):369-81.
5. Wong VK, Baker S, Pickard DJ, Parkhill J, Page AJ, Feasey NA, et al. Phylogeographical analysis of the dominant multidrug-resistant H58 clade of Salmonella Typhi identifies inter- and intracontinental transmission events. Nature Genetics. 2015;47(6):632-9.
6. Walker J, Chaguza C, Grubaugh ND, Carey M, Baker S, Khan K, et al. Assessing the global risk of typhoid outbreaks caused by extensively drug resistant Salmonella Typhi. Nature Communications. 2023;14(1):6502.

7. Jamilah J, Hatta M, Natzir R, Umar F, Sjahril R, Agus R, et al. Analysis of existence of multidrug-resistant H58 gene in Salmonella enterica serovar Typhi isolated from typhoid fever patients in Makassar, Indonesia. New Microbes and New Infections. 2020;38:100793.
8. Nizamuddin S, Ching C, Kamal R, Zaman MH, Sultan F. Continued Outbreak of Ceftriaxone-Resistant Salmonella enterica Serotype Typhi across Pakistan and Assessment of Knowledge and Practices among Healthcare Workers. Am J Trop Med Hyg. 2021;104(4):1265-70.
9. Jabeen K, Saleem S, Jahan S, Nizamudin S, Arshad F, Huma Z-e, et al. Molecular Characterization of Extensively Drug Resistant Salmonella Enterica Serovar Typhi Clinical Isolates from Lahore, Pakistan. Infection and Drug Resistance. 2023;16(null):2987-3001.
10. Saeed M, Rasool MH, Rasheed F, Saqalein M, Nisar MA, Imran AA, et al. Extended-spectrum beta-lactamases producing extensively drug-resistant Salmonella Typhi in Punjab, Pakistan. The Journal of Infection in Developing Countries. 2020;14(02):169-76.
11. Kim C, Latif I, Neupane DP, Lee GY, Kwon RS, Batool A, et al. The molecular basis of extensively drug-resistant Salmonella Typhi isolates from pediatric septicemia patients. PLOS ONE. 2021;16(9):e0257744.
12. Sun J, Deng Z, Yan A. Bacterial multidrug efflux pumps: Mechanisms, physiology and pharmacological exploitations. Biochemical and Biophysical Research Communications. 2014;453(2):254-67.
13. Alenazy R. Antibiotic resistance in Salmonella: Targeting multidrug resistance by understanding efflux pumps, regulators and the inhibitors. Journal of King Saud University - Science. 2022;34(7):102275.
14. Rasheed F, Saeed M, Alikhan N-F, Baker D, Khurshid M, Ainsworth EV, et al. Emergence of Resistance to Fluoroquinolones and Third-Generation Cephalosporins in Salmonella Typhi in Lahore, Pakistan. Microorganisms [Internet]. 2020; 8(9).
15. Mumtaz MN, Irfan M, Siraj S, Khan A, Khan H, Imran M, et al. Whole-genome sequencing of extensively drug-resistant Salmonella enterica serovar Typhi clinical isolates from the Peshawar region of Pakistan. Journal of Infection and Public Health. 2024;17(2):271-82.
16. Butt MH, Saleem A, Javed SO, Ullah I, Rehman MU, Islam N, et al. Rising XDR-Typhoid Fever Cases in Pakistan: Are We Heading Back to the Pre-antibiotic Era? Frontiers in public health. 2021;9:794868.
17. Kumawat M, Nabi B, Daswani M, Viquar I, Pal N, Sharma P, et al. Role of bacterial efflux pump proteins in antibiotic resistance across microbial species. Microbial Pathogenesis. 2023;181:106182.
18. Belay WY, Getachew M, Tegegne BA, Teffera ZH, Dagne A, Zeleke TK, et al. Mechanism of antibacterial resistance, strategies and next-generation antimicrobials to contain antimicrobial resistance: a review. 2024;15.
19. Blanco P, Hernando-Amado S, Reales-Calderon JA, Corona F, Lira F, Alcalde-Rico M, et al. Bacterial Multidrug Efflux Pumps: Much More Than Antibiotic Resistance Determinants. Microorganisms [Internet]. 2016; 4(1).
20. Van Bambeke F, Balzi E, Tulkens PM. Antibiotic efflux pumps. Biochemical Pharmacology. 2000;60(4):457-70.
21. Garcia ÍR, de Oliveira Garcia FA, Pereira PS, Coutinho HDM, Siyadatpanah A, Norouzi R, et al. Microbial resistance: The role of efflux pump superfamilies and their respective substrates. Life Sciences. 2022;295:120391.
22. Li X-Z, Plésiat P, Nikaido H. The Challenge of Efflux-Mediated Antibiotic Resistance in Gram-Negative Bacteria. Clinical Microbiology Reviews. 2015;28(2):337-418.
23. Farhat N, Ali A, Bonomo RA, Khan AU. Efflux pumps as interventions to control infection caused by drug-resistance bacteria. Drug Discovery Today. 2020;25(12):2307-16.

24. Lubelski J, Konings Wil N, Driessen Arnold JM. Distribution and Physiology of ABC-Type Transporters Contributing to Multidrug Resistance in Bacteria. Microbiology and Molecular Biology Reviews. 2007;71(3):463-76.
25. Dawan J, Li Y, Lu F, He X, Ahn J. Role of Efflux Pump-Mediated Antibiotic Resistance in Quorum Sensing-Regulated Biofilm Formation by Salmonella Typhimurium. Pathogens [Internet]. 2022; 11(2).
26. Chen T, Xiao Z, Liu X, Wang T, Wang Y, Ye F, et al. Natural products for combating multidrug resistance in cancer. Pharmacological Research. 2024;202:107099.
27. Shafique I, Rafiq M, Rana NF, Menaa F, Almalki F, Aljuaid A, et al. Computational evaluation of efflux pump homologues and lignans as potent inhibitors against multidrug-resistant Salmonella typhi. PLOS ONE. 2024;19(6):e0303285.
28. Mina SA, Hasan MZ, Hossain AKMZ, Barua A, Mirjada MR, Chowdhury AMMA. The Prevalence of Multi-Drug Resistant Salmonella typhi Isolated From Blood Sample. Microbiology Insights. 2023;16:11786361221150760.
29. Ahmad S, Tsagkaris C, Aborode AT, Ul Haque MT, Khan SI, Khawaja UA, et al. A skeleton in the closet: The implications of COVID-19 on XDR strain of typhoid in Pakistan. Public health in practice (Oxford, England). 2021;2:100084.
30. Fatima S, Ishaq Z, Irfan M, AlAsmari AF, Achakzai JK, Zaheer T, et al. Whole-genome sequencing of multidrug resistance Salmonella Typhi clinical strains isolated from Balochistan, Pakistan. 2023;11.
31. Mehta J, Rolta R, Dev K. Role of medicinal plants from North Western Himalayas as an efflux pump inhibitor against MDR AcrAB-TolC Salmonella enterica serovar typhimurium: In vitro and In silico studies. Journal of Ethnopharmacology. 2022;282:114589.
32. Zgurskaya HI. Introduction: Transporters, Porins, and Efflux Pumps. Chemical Reviews. 2021;121(9):5095-7.
33. Mehta J, Rolta R, Dev K. Role of medicinal plants from North Western Himalayas as an efflux pump inhibitor against MDR AcrAB-TolC Salmonella enterica serovar typhimurium: In vitro and In silico studies. Journal of ethnopharmacology. 2022;282:114589.
34. Tan X, Huang Y, Rana A, Singh N, Abbey TC, Chen H, et al. Optimization of an in vitro Pseudomonas aeruginosa Biofilm Model to Examine Antibiotic Pharmacodynamics at the Air-Liquid Interface. npj Biofilms and Microbiomes. 2024;10(1):16.
35. Mastoor S, Nazim F, Rizwan-ul-Hasan S, Ahmed K, Khan S, Ali SN, et al. Analysis of the Antimicrobial and Anti-Biofilm Activity of Natural Compounds and Their Analogues against Staphylococcus aureus Isolates. Molecules [Internet]. 2022; 27(20).
36. Šimunović K, Solnier J, Alperth F, Kunert O, Smole Možina S, Bucar F. Efflux Pump Inhibition and Resistance Modulation in Mycobacterium smegmatis by Peucedanum ostruthium and Its Coumarins. Antibiotics [Internet]. 2021; 10(9).
37. Cui SM, Li T, Wang Q, He KK, Zheng YM, Liang HY, et al. Antibacterial Effects of Schisandra chinensis Extract on Escherichia coli and its Applications in Cosmetic. Current Microbiology. 2020;77(5):865-74.
38. Fan Q, He Q, Zhang T, Song W, Sheng Q, Yuan Y, et al. Antibiofilm potential of lactobionic acid against Salmonella Typhimurium. LWT. 2022;162:113461.
39. Moshiri J, Kaur D, Hambira CM, Sandala JL, Koopman JA, Fuchs JR, et al. Identification of a Small Molecule Anti-biofilm Agent Against Salmonella enterica. Frontiers in Microbiology. 2018;9.
40. Verma SK, Jha E, Panda PK, Das JK, Thirumurugan A, Suar M, et al. Molecular Aspects of core-shell Intrinsic Defect Induced Enhanced Antibacterial Activity of ZnO Nanocrystals. Nanomedicine. 2018;13(1):43-68.
41. Gangathraprabhu B, Kannan S, Santhanam G, Suryadevara N, Maruthamuthu M. A review on the origin of multidrug-resistant Salmonella and perspective of tailored phoP gene towards avirulence. Microbial Pathogenesis. 2020;147:104352.

42. Browne AJ, Chipeta MG, Fell FJ, Haines-Woodhouse G, Kashef Hamadani BH, Kumaran EAP, et al. Estimating the subnational prevalence of antimicrobial resistant Salmonella enterica serovars Typhi and Paratyphi A infections in 75 endemic countries, 1990–2019: a modelling study. The Lancet Global Health. 2024;12(3):e406-e18.
43. Clinical, Institute LS. Performance standards for antimicrobial susceptibility testing. Clinical and Laboratory Standards Institute Wayne, PA; 2020.
44. Amsalu T, Genet C, Adem Siraj Y. Salmonella Typhi and Salmonella Paratyphi prevalence, antimicrobial susceptibility profile and factors associated with enteric fever infection in Bahir Dar, Ethiopia. Scientific Reports. 2021;11(1):7359.
45. Yang C, Li H, Zhang T, Chu Y, Zuo J, Chen D. Study on antibiotic susceptibility of Salmonella typhimurium L forms to the third and forth generation cephalosporins. Scientific Reports. 2020;10(1):3042.
46. Behera DU, Gaur M, Sahoo M, Subudhi E, Subudhi BB. Development of pharmacophore models for AcrB protein and the identification of potential adjuvant candidates for overcoming efflux-mediated colistin resistance. RSC Medicinal Chemistry. 2024;15(1):127-38.
47. Martins M, McCusker MP, Viveiros M, Couto I, Fanning S, Pagès JM, et al. A Simple Method for Assessment of MDR Bacteria for Over-Expressed Efflux Pumps. Open Microbiol J. 2013;7:72-82.
48. Abdel-Karim SA-AM, El-Ganiny AMA, El-Sayed MA, Abbas HAA. Promising FDA-approved drugs with efflux pump inhibitory activities against clinical isolates of Staphylococcus aureus. PLOS ONE. 2022;17(7):e0272417.
49. Al-Sallami D, Alsultan A, Abbas KH, Clarke SRJOVJ. Evaluation of efflux pump inhibitory activity of some plant extracts and using them as adjuvants to potentiate the inhibitory activity of some antibiotics against Staphylococcus aureus. 2023;13(1):42-7.
50. Kim S-H, Jyung S, Kang D-H. Comparative study of Salmonella Typhimurium biofilms and their resistance depending on cellulose secretion and maturation temperatures. LWT. 2022;154:112700.
51. Kang S, Kong F, Shi X, Han H, Li M, Guan B, et al. Antibacterial activity and mechanism of lactobionic acid against Pseudomonas fluorescens and Methicillin-resistant Staphylococcus aureus and its application on whole milk. Food Control. 2020;108:106876.
52. Cui SM, Li T, Wang Q, He KK, Zheng YM, Liang HY, et al. Antibacterial Effects of Schisandra chinensis Extract on Escherichia coli and its Applications in Cosmetic. Current microbiology. 2020;77(5):865-74.
53. Skalski B, Kuźniak E, Kowalska I, Sikora M, Olas B. A Review of the Biological Activity and Structure–Property Relationships of the Main Compounds from Schisandra chinensis. Nutrients. 2025;17(3):436.
54. Singh VK, Singh A, Singh A. Plants Based Drug Discovery Update for Anti-Hiv Disease Management. BIOPHARMACOLOGICAL ACTIVITIES OF MEDICINAL PLANTS AND BIOACTIVE COMPOUNDS.193.
55. Gao L, Liu X, Luo X, Lou X, Li P, Li X, et al. Antiaging effects of dietary supplements and natural products. Frontiers in Pharmacology. 2023;14:1192714.
56. Menaa F, Menaa A, Tréton J. Polyphenols against skin aging. Polyphenols in human health and disease: Elsevier; 2014. p. 819-30.
57. Ekalu A, Ayo RG-O, Habila JD, Hamisu I. In vitro antimicrobial activity of lignan from the stem bark of Strombosia grandifolia Hook. f. ex Benth. Bulletin of the National Research Centre. 2019;43:1-7.
