# Supplementary material for: Schisandrins as novel efflux pumps inhibitors and non-antibiotic compounds against multi- and extensively-drug resistant clinical strains of Salmonella typhi: An in-vitro study
Source: PLoS One. 2026 Jul 31;21(7):e0347214. doi: 10.1371/journal.pone.0347214 (PMC13426931; doi:10.1371/journal.pone.0347214)
Supplement: Attachment — Submitted filename: Response to Reviewers.docx [file pone.0347214.s005.docx]

### **Dear Editor and Reviewers,**

Dear Editor and Reviewers,

We are very grateful for your helpful comments and suggestions for the manuscript. We appreciate your insightful opinions. We have made changes (Red color text) to the manuscript accordingly, to ensure accuracy and consistency. We, and a native American scientist, also went through the entire manuscript to correct any typos and grammatical mistakes. The references have been updated strictly following the journal’s guidelines. A clean version as well as a version with the track changes are provided.

We believe that this new version of the manuscript is now much improved and hope it will be given full consideration for publication to *PlosOne*.

With best regards,

The authors

### **Response to Reviewer #1**

*Thank you for your valuable feedback and suggestions regarding our manuscript.*

**Comment1:**
In the introduction section, the author provides background but does not clearly articulate the research gap. Why is this study needed in the current context? Lacks updated global and regional statistics (AMR trends, WHO data). No specific hypothesis or research question stated. It should be included in the manuscript.

**Response:**
We appreciate the reviewer’s valuable feedback. The introduction has been revised to clearly define the research gap and emphasize the importance of this study in the context of the current global rise in antimicrobial resistance (AMR). Updated statistics have been incorporated from the **World Health Organization’s Global Antimicrobial Resistance and Use Surveillance System (GLASS) Report 2024** and **regional surveillance data from South Asia**. These updates highlight the alarming increase in multidrug-resistant pathogens and justify the need for the present investigation. Additionally, a clear hypothesis and research question have now been explicitly stated.

(Page 2, Lines 45–70)

**Comment2:**
In the methodology section, it was unclear whether the sampling was random or convenience sampling; there was no justification for sample size (power calculation missing). Inclusion/Exclusion Criteria not clearly defined. CLSI guidelines mentioned no version/year cited, and no details on quality control strains.

**Response:**
We obtained all isolates that were available during the study period, and from these, we further selected only those that fulfilled the standard MDR and XDR criteria, which were relevant to our research objectives. Regarding sample size, a formal power calculation was not applicable because the study was based on all MDR/XDR isolates available in the diagnostic laboratory during the specific time frame. So, the samples were collected from patients presenting at the diagnostic center between July 1 and July 10, 2024, who were suspected of having *Salmonella typhi* infection and required antibiotic susceptibility testing, from this multidrug resistance (MDR) and extensively drug resistance (XDR) were isolated for further analysis.

We have also cited the most recent **CLSI 2023 Performance Standards for Antimicrobial Susceptibility Testing (33rd Edition)**

**Reference:**
Clinical and Laboratory Standards Institute (CLSI). Performance Standards for Antimicrobial Susceptibility Testing, 33rd Edition (M100, 2023). https://clsi.org/standards/products/microbiology/documents/m100/
(Page 4–5, Lines 110–145)

**Comment3:**
In the results section, resistance patterns were described, but no molecular characterization (genes, plasmid types), which limits depth.

**Response:**
Although this study successfully characterized the phenotypic antimicrobial resistance profiles of the Salmonella enterica serovar *Typhi* isolates, molecular characterization of resistance determinants (such as specific genes, plasmid replicon types and mobile genetic elements) was beyond the objectives of the present work. Our primary aim was to investigate the potential role of efflux-pump inhibitors in *S. typhi*, an area that, to our knowledge, has not previously been explored in this organism. Indeed, in our earlier study (Shafique I. et al., PLOS ONE, 2024) we applied computational methods to identify putative efflux-pump homologues and assess lignan compounds as efflux-pump inhibitors in S. Typhi. Therefore, while the current study delivers important baseline phenotypic data, future work should incorporate detailed molecular investigations to elucidate the underlying genetic architecture of resistance mechanisms and to complement the inhibitor-focused objectives of our research. Our study directly points towards the importance of efflux-pump systems as one of the viable therapeutic targets in combating resistant Salmonella enterica serovar typhi strains. However, we have added a note in the discussion section acknowledging this limitation.

(Page 7, Lines 210–220)

**Comment4:**
In the discussion part, there was no critical comparison with international studies – does not contextualize findings globally. Overlooks important drivers of AMR (e.g., antimicrobial stewardship, infection control). No policy implications or practical recommendations.

**Response:**
The discussion section has been extensively revised to include **comparative analysis with global studies**, particularly from the **European Centre for Disease Prevention and Control (ECDC, 2024)** and **Centers for Disease Control and Prevention (CDC, 2023)** reports. The discussion now elaborates on **drivers of AMR**, such as poor infection control, unregulated antibiotic use, and gaps in antimicrobial stewardship programs. Furthermore, **policy implications and practical recommendations** have been incorporated, emphasizing the need for **strengthened national AMR surveillance, stewardship training, and public awareness**.
(Page 9–10, Lines 270–310)

### **Response to Reviewer #2**

**GeneralComment:**
We sincerely thank the reviewer for their encouraging remarks and for recognizing our effort. All suggested corrections and clarifications have been addressed in the revised manuscript, with corresponding track changes visible.

**Comment (Line 70–71):** Replace “irregular bowel movements” with “diarrhoea or constipation,” which are the clinically accepted descriptions. Global and regional burden statistics are important but feel fragmented.

**Response:**
The phrase has been replaced with “diarrhea or constipation” as suggested. Additionally, the global and regional burden data have been reorganized into separate subsections to improve flow and clarity, now presented as: (1) Global burden (cases and deaths), (2) Regional burden (South Asia, Pakistan), and (3) Recent outbreaks and XDR reports.
(Page 2, Lines 70–75)

**Comment (Line 79–80):** The phrase “driven by an XDR strain, resulting in the emergence of an MDR H58 strain” is confusing. Clarify the chronology: did XDR arise from the H58 lineage? Line 80, page 3: Terms like “XDR strain,” “MDR H58 strain,” and “XDR bacterial pathogens” are used interchangeably, which may confuse readers. Please define MDR, XDR, and the H58 lineage clearly, and stick to consistent terminology.

**Response:**
We have revised this section to clarify that the XDR Salmonella Typhi strain **arose from the MDR H58 lineage**. Consistent terminology is now used throughout, with clear definitions of MDR (multidrug-resistant), XDR (extensively drug-resistant), and H58 lineage provided in the introduction. The XDR variant (resistance to first-line drugs plus fluoroquinolones and third-generation cephalosporins) did not spontaneously emerge independently outside H58, but rather within the H58 lineage. Several genomic studies indicate that a pre-existing MDR H58 clone acquired an additional plasmid carrying extended-spectrum β-lactamase (ESBL) genes (e.g., bla CTX-M-15) plus quinolone resistance genes (qnrS1), turning it into XDR.

Reference:

*Carey, M. E., Thi Nguyen, T. N., Tran, D. H. N., Dyson, Z. A., Keane, J. A., Pham Thanh, D., ... & Baker, S. (2024). The origins of haplotype 58 (H58) Salmonella enterica serovar Typhi. Communications Biology, 7(1), 775*

This sentence was added to the manuscript in the context of highlighting the relevance to efflux pump inhibitors: Because it already carries multiple resistance determinants, it represents a “high‐risk” background for further resistance evolution. Apart from classical MDR genes, H58 strains have acquired further resistance (fluoroquinolones, cephalosporins) and plasmids in XDR contexts. That means efflux‐pumps and other resistance mechanisms likely co‐operate in this lineage
(Page 3, Lines 78–82)

**Comment (Line 83):** Add sentence: “The global rise of multidrug-resistant (MDR) microorganisms causing infections is accelerating, with the problem being particularly severe in developing countries.” Use updated references.

**Response:**
The suggested sentence has been added to strengthen the introduction. The following updated references have also been included:

- Hussein RA, AL-Kubaisy SH, Al-Ouqaili MTS. The influence of efflux pump, outer membrane permeability and β-lactamase production on the resistance profile of multi, extensively and pandrug-resistant Klebsiella pneumoniae. J Infect Public Health. 2024;17(11):102544. <https://doi.org/10.1016/j.jiph.2024.102544>
- Owaid HA, Al-Ouqaili MTS. Molecular and bacteriological investigations for the co-existence of CRISPR/Cas system and β-lactamases of types extended-spectrum and carbapenemases in MDR, XDR and PDR Klebsiella pneumoniae. Saudi J Biol Sci. 2024;31(7):104022. https://doi.org/10.1016/j.sjbs.2024.104022
  (Page 3, Lines 83–89)

**Comment (Line 90):** Line 90: Typo/grammar issue: “is concerned about” → should be “raises concerns about.” The role of EPs is well described, but the section (lines 91–103) could be condensed and made more focused. Currently, it mixes resistance, virulence, biofilm, and transporter families without transitions. Suggest splitting: Efflux pumps in resistance (mechanisms, prevalence) Efflux pumps in biofilm and virulence Importance of EPIs Line 92–93: “bacteria cell” → should be “bacterial cell.”

**Response:**
The grammatical correction has been made. The efflux pump section was reorganized into three concise subsections:

1. Efflux pumps in antimicrobial resistance (mechanisms and prevalence)
2. Efflux pumps in biofilm formation and virulence
3. Importance of efflux pump inhibitors (EPIs)
   Transitions between these topics have been improved for clarity.
   (Page 3–4, Lines 90–103)

**Comment (Line 96, Page 4):** Add reference: Al-Ouqaili MTS et al., 2025, PLoS One.

**Response:**
The suggested reference has been incorporated to strengthen the introduction and highlight recent evidence of carbapenemase-encoding genes in hospital pathogens.

- Al-Ouqaili MTS, Hussein RA, Kanaan BA, Al-Neda MTS. Investigation of carbapenemase-encoding genes in Burkholderia cepacia and Aeromonas sobria isolates from nosocomial infections in Iraqi patients. PLoS One. 2025;20(8):e0315490. <https://doi.org/10.1371/journal.pone.0315490>
  (Page 4, Lines 96–99)

**Comment (Line 101–102):** “Plasma membrane organelles” is inaccurate ABC transporters are membrane proteins, not organelles

**Response:**
Corrected to “membrane-bound transport proteins” to accurately describe ABC transporters.
(Page 4, Line 101)

**Comment (Line 133, Page 5):** The section states blood samples were collected but does not mention patient numbers, inclusion/exclusion criteria, or demographics.

**Response:**
We obtained all isolates that were available during the study period, and from these, we further selected only those that fulfilled the standard MDR and XDR criteria, which were relevant to our research objectives. Regarding sample size, a formal power calculation was not applicable because the study was based on all MDR/XDR isolates available in the diagnostic laboratory during the specific time frame. So the samples were collected from patients presenting at the diagnostic center between July 1 and July 10, 2024, who were suspected of having Salmonella typhi infection and required antibiotic susceptibility testing, from this multidrug resistance (MDR) and extensively drug resistance (XDR) were isolated for further analysis.

**Comment (Line 158, Page 6):** CLSI or EUCAST reference required.

**Response:**
The **CLSI 2023 guidelines** have been cited for antimicrobial susceptibility testing.
Reference:
Clinical and Laboratory Standards Institute (CLSI). Performance Standards for Antimicrobial Susceptibility Testing, 33rd Edition (M100, 2023). https://clsi.org/standards/products/microbiology/documents/m100/
(Page 6, Lines 155–160)

**Comment (Line 217–218, 243–244):** While methods are comprehensive, it’s unclear why specific concentrations were chosen (e.g., 0.5–40 μg/mL for MIC; 2× MIC for SEM). A justification (based on literature or preliminary screening) should be provided.

**Response:**
Concentration ranges (0.5–40 μg/mL for MIC and 2× MIC for SEM) were selected based on **previous studies reporting effective inhibitory ranges** for efflux pump inhibitors. Relevant literature citations have been added.
(Page 8–9, Lines 217–244)

**Comment (Line 270–271):** Specify bacterial type instead of “presence of bacteria.”

**Response:**
The statement has been revised to “non-lactose fermenting colonies consistent with Salmonella spp. were observed.”
(Page 10, Line 270)

**Comment (Lines 294–299):** Only four isolates were tested (1 MDR, 1 XDR, 2 sensitive). This is too small a sample size to draw robust conclusions about resistance mechanisms, biofilm formation, or EPI activity. Please justify the limited number of isolates and clearly state this as a limitation.

**Response:**
We acknowledge the limited number of isolates tested. A justification has been added, noting the **focus on representative isolates** to perform detailed phenotypic and microscopic analysis within available biosafety and funding constraints. This limitation is explicitly mentioned in the discussion.
(Page 11, Lines 294–299; Page 26, Lines 500–505)

**Comment (Line 309–311):** Awkward phrasing: “The intensity of fluorescence was lowly detectable in MDR and undetectable in XDR…” → Suggest “Fluorescence intensity was weak in MDR and absent in XDR isolates

**Response:**
Revised to: “Fluorescence intensity was weak in MDR and absent in XDR isolates.”
(Page 12, Lines 309–311)

**Comment (Lines 321–331):** The figure is not clear enough to convincingly demonstrate fluorescence differences among MDR, XDR, and sensitive strains. Please consider.

**Response:**
Figure 3 has been enhanced with **higher resolution**,.
(Figure 3 and Legend, Page 13)

**Comment (Lines 337–349; 400–413; 465–471):** P-values are present, but statistical test details are missing (e.g., t-test, ANOVA, repeated measures?). Must specify the test used, replicates (n), and confidence intervals where possible. Increasing image resolution and brightness/contrast to make fluorescence intensity more distinguishable. Adding a clear scale bar and indicating the exposure time/UV source used for visualisation. Including quantification of fluorescence intensity (e.g., densitometry or ImageJ analysis) to support the visual observations. Providing side-by-side comparison panels with labelled arrows or annotations highlighting key differences between strains. Using a negative control plate without EtBr to eliminate background signals.

**Response:**
**We thank the reviewer for these valuable comments regarding the statistical analysis and image presentation.** The statistical tests used are now specified: **one-way ANOVA**, with significance set at p < 0.05. Replicate numbers (n = 3) and 95% confidence intervals are included. We have also improved the image clarity by adjusting resolution, brightness, and contrast, and we have added scale bars and detailed imaging parameters (UV source and exposure conditions) to strengthen the reproducibility of our qualitative observations.
 **We would like to respectfully clarify that the present study was designed and conducted as a qualitative, exploratory investigation rather than a quantitative or confirmatory analysis. Our primary objective was to visualize and describe fluorescence patterns between the tested isolates, not to perform statistical comparisons of intensity levels. For this reason, we did not include negative control plates without EtBr nor perform densitometry/ImageJ quantification, as our study was not intended to provide a quantitative fluorescence assessment. We fully acknowledge the importance of rigorous quantitative analysis and appropriate controls in future confirmatory studies. In this initial work, however, our focus was limited to establishing proof-of-concept qualitative differences, which we have now emphasized more clearly in the revised manuscript to avoid misinterpretation.** (Page 15–17, Lines 337–471)

**Comment (Line 368–373):** Biofilm OD classification should be consistent with the cutoff criteria given in Methods (weak, moderate, strong). Currently, the interpretation looks inconsistent

**Response:**
Biofilm strength classifications (weak, moderate, strong) have been rechecked and standardized according to the criteria defined in the Methods section.
(Page 14, Lines 368–373)

**Comment (Lines 389–406):** MIC results are vague (“45–50% inhibition at 10–20 μg/mL”). By definition, MIC should be the lowest concentration that prevents visible growth. The current description looks more like IC₅₀ values. Please clarify methodology and correct terminology.

**Response:**
The MIC values have been clarified as the **lowest concentration preventing visible growth**, distinguishing them from IC₅₀ values. Descriptions have been revised accordingly.
(Page 16, Lines 389–406)

**Comment (Line 490–494):** Resistance results are mentioned but not linked strongly to clinical relevance. The discussion would benefit from contextualization — e.g., how the resistance profile in your isolates compares quantitatively with national or WHO surveillance data

**Response:**
We have added discussion linking our resistance data to **national and WHO AMR surveillance reports**, highlighting the clinical significance of observed resistance levels in the regional context.
(Page 24, Lines 490–494)

**Comment (Line 503–511):** Add limitations to avoid overstatement of EPI activity.

**Response:**
We have revised the statement to acknowledge study limitations — specifically that findings are **based on in vitro assays** and **lack in vivo validation**. Future research directions are added to confirm EPI effects in clinical settings.
(Page 25, Lines 503–511)

**Comment (Line 515, Page 27):** Add reference: Al-Ouqaili et al., 2018, Asian Journal of Pharmaceutics.

**Response:**
The reference has been added to strengthen discussion on biofilm susceptibility and EPI mechanisms.

- Al-Ouqaili MTS, Al-Kubaisy SHM, Al-Ani NFI. Biofilm antimicrobial susceptibility pattern for selected antimicrobial agents against planktonic and sessile cells of clinical isolates of staphylococci. Asian J Pharm. 2018;12(4):S1375–S1383. <https://doi.org/10.22377/ajp.v12i04.2938>
  (Page 27, Line 515)

**Comment (Line 576):** Conclusion should include future perspective.

**Response:**
The conclusion has been expanded to include **future perspectives**, emphasizing molecular exploration of efflux mechanisms, testing of novel EPIs, and in vivo validation studies to confirm clinical applicability.
(Page 28, Lines 576–580)
