# Supplementary material for: Schisandrins as novel efflux pumps inhibitors and non-antibiotic compounds against multi- and extensively-drug resistant clinical strains of Salmonella typhi: An in-vitro study
Source: PLoS One. 2026 Jul 31;21(7):e0347214. doi: 10.1371/journal.pone.0347214 (PMC13426931; doi:10.1371/journal.pone.0347214)
Supplement: Attachment — Submitted filename: Response_to_Reviewers_auresp_2.docx [file pone.0347214.s006.docx]

Dear Editor and Reviewers,

We are very grateful for your helpful comments and suggestions for the manuscript. We appreciate your insightful opinions. We have made changes (Red color text) to the manuscript accordingly, to ensure accuracy and consistency. We also went through the entire manuscript to correct any typos. A clean version as well as a version with the track changes are provided.

We believe that this new version of the manuscript is now much improved and will be ready for publication to *PlosOne*.

With best regards,

The authors

### **Response to Reviewer #3**

*Thank you for your valuable feedback and suggestions regarding our manuscript.*

**Comment1:**
most important one: please add units and proper labels to the Y-axes of Figures 6 and 9.

**Response:**

The Y-axes of Figures 6 and 9 have now been revised to include appropriate labels and units to improve clarity. The updated figures have been incorporated into the revised manuscript.

**Comment2:**
In Figure 7, why does growth recording begin at 2 h rather than at the initial time (t = 0)? It is important to demonstrate that all cultures start with approximately the same absorbance (OD) value.

**Response:**

**In our experimental design, all bacterial cultures were standardized prior to incubation by adjusting the inoculum to the same initial optical density (OD₆₀₀ ≈ 0.05), ensuring comparable starting cell densities across all experimental groups. The first recorded time point presented in Figure 7 corresponds to 2 h because measurements at t = 0 primarily reflect the inoculum preparation step rather than active bacterial growth. We began growth recording at 2 h because the bacterial culture requires approximately 2 hours to reach the exponential phase. After this point, the compound was added to ensure its effect was evaluated during active bacterial growth rather than during the lag phase. Therefore, measurements were recorded from 2 h onward.**

**Comment3:**
In Figure 9, the antibiofilm activity appears very potent already at the lowest concentration tested (3 µg/mL) and shows little to no further increase at higher concentrations. It would be valuable to test 1–2 lower concentrations (e.g., 0.5–1 µg/mL) to better characterize the dose-response relationship

**Response:**
We also evaluated concentrations below 3 µg/mL; however, the inhibition at these levels was not sufficiently significant. The concentration that produced approximately 50% inhibition was above 3 µg/mL. Therefore, we plotted the graph starting from 3 µg/mL which is which is why we focused our dose–response analysis starting from 3 µg/mL. We followed this approach based on a published study, which has been added in our manuscript.

*Reference*

*Kaur, V., Kaushal, S., Kalia, A., Jangra, R., & Sharma, P. (2025). Antimicrobial, synergistic and antibiofilm activities of Myristica fragrans Houtt. Bioactive compounds and their derivatives. Scientific Reports, 15(1), 33041.*

*(Page 28 , Line 528-530 )*

Comment 4:

An evaluation of whether the tested compounds are bactericidal or bacteriostatic could have been performed using bacterial live/dead staining.

**Response:**

Regarding the reviewer’s suggestion to distinguish between bactericidal and bacteriostatic activity using bacterial live/dead staining, we agree that such analysis could provide additional mechanistic insight. However, the primary objective of this work was to evaluate the antibiofilm potential of the compounds. Future studies incorporating live/dead staining or similar viability assays will be undertaken to further elucidate the mechanism of antibiofilm action.

Comment 5:

Gram” should be capitalized (e.g., Gram-positive, Gram-negative).

Response:

The term “Gram” has now been correctly capitalized throughout the manuscript (e.g., Gram-positive and Gram-negative).

Comment 6:

There are many missing spaces between words, especially after references (e.g., line 538: “recently(53)” should be “recently (53)”). Please also correct any remaining double spaces throughout the manuscript

Response:

Thank you for pointing this out. We have carefully reviewed the entire manuscript and corrected the missing spaces between words, particularly after references (e.g., “recently (53)”). In addition, all remaining double spaces throughout the manuscript have been removed to ensure consistency and proper formatting

### **Response to Reviewer #4**

**Generalcomments:**
We sincerely thank the reviewers for their encouraging remarks and for recognizing our effort. Thank you for your positive and encouraging feedback. We sincerely appreciate the reviewer’s thoughtful evaluation of our manuscript and the recognition of the revisions made. We are grateful for the acknowledgment of the study’s contribution in exploring Schisandrins as potential efflux pump inhibitors against MDR and XDR *Salmonella typhi*. Your valuable comments and suggestions have helped us further strengthen the quality and clarity of our work.

**Comment 1:**

**There is, however, one remaining issue regarding journal policy that must be resolved prior to final acceptance:**

**1. Data Availability Requirement: In the current manuscript, the data availability statement indicates that data is available "upon reasonable request." Please note that the PLOS Data policy strictly requires all data underlying the findings to be fully available without restriction. Stating that data is available upon request is not sufficient. You must upload the minimal raw dataset (e.g., the underlying spreadsheet data behind your graphs, raw CFU counts, and uncropped images) as part of the manuscript's Supporting Information files or deposit it into a recognized public repository (providing the DOI/accession number).**

**Response:**
We have now revised the Data Availability Statement to ensure full compliance with the PLOS data policy. The minimal raw datasets underlying the findings of this study, including the spreadsheet data used to generate the graphs have been uploaded as Supporting Information files with the manuscript. This ensures that all underlying data is fully accessible without restriction.
