# Supplementary material for: Schisandrins as novel efflux pumps inhibitors and non-antibiotic compounds against multi- and extensively-drug resistant clinical strains of Salmonella typhi: An in-vitro study
Source: PLoS One. 2026 Jul 31;21(7):e0347214. doi: 10.1371/journal.pone.0347214 (PMC13426931; doi:10.1371/journal.pone.0347214)
Supplement: Peer review history

# Peer review: Decision Letter 0

**DOI:** 10.1371/journal.pone.0347214.r001

**Authors:** Furqan Kabir

17 Sep 2025

PONE-D-25-32847

Schisandrins as Novel Efflux Pumps Inhibitors and Non-Antibiotic Compounds Against Multi- and Extensively-Drug Resistant Clinical strains of Salmonella typhi:  An In-Vitro Study

PLOS ONE

Dear Dr. Rana,

Thank you for submitting your manuscript to PLOS ONE. After careful consideration, we feel that it has merit but does not fully meet PLOS ONE’s publication criteria as it currently stands. Therefore, we invite you to submit a revised version of the manuscript that addresses the points raised during the review process.

Please review the comments carefully and draft your responses along with the manuscript amendment accordingly.

Please submit your revised manuscript by Oct 31 2025 11:59PM. If you will need more time than this to complete your revisions, please reply to this message or contact the journal office at plosone@plos.org. When you're ready to submit your revision, log on to https://www.editorialmanager.com/pone/ and select the 'Submissions Needing Revision' folder to locate your manuscript file.

Please include the following items when submitting your revised manuscript:

- A rebuttal letter that responds to each point raised by the academic editor and reviewer(s). You should upload this letter as a separate file labeled 'Response to Reviewers'.
- A marked-up copy of your manuscript that highlights changes made to the original version. You should upload this as a separate file labeled 'Revised Manuscript with Track Changes'.
- An unmarked version of your revised paper without tracked changes. You should upload this as a separate file labeled 'Manuscript'.

If you would like to make changes to your financial disclosure, please include your updated statement in your cover letter. Guidelines for resubmitting your figure files are available below the reviewer comments at the end of this letter.

If applicable, we recommend that you deposit your laboratory protocols in protocols.io to enhance the reproducibility of your results. Protocols.io assigns your protocol its own identifier (DOI) so that it can be cited independently in the future. For instructions see: https://journals.plos.org/plosone/s/submission-guidelines#loc-laboratory-protocols. Additionally, PLOS ONE offers an option for publishing peer-reviewed Lab Protocol articles, which describe protocols hosted on protocols.io. Read more information on sharing protocols at https://plos.org/protocols?utm_medium=editorial-email&utm_source=authorletters&utm_campaign=protocols.

We look forward to receiving your revised manuscript.

Kind regards,

Furqan Kabir

Academic Editor

PLOS ONE

Journal Requirements:

When submitting your revision, we need you to address these additional requirements.

1. Please ensure that your manuscript meets PLOS ONE's style requirements, including those for file naming. The PLOS ONE style templates can be found at

https://journals.plos.org/plosone/s/file?id=wjVg/PLOSOne_formatting_sample_main_body.pdf  and

https://journals.plos.org/plosone/s/file?id=ba62/PLOSOne_formatting_sample_title_authors_affiliations.pdf

2. In your Methods section, could you please include any relevant details about the SA and SB products used, including their purity and any chemical authentications you performed.

3. Thank you for stating the following financial disclosure:

“This research was funded through large Groups (Project under grant number R.G.P. 2/344/44), Deanship of Scientific Research and Graduate Studies at King Khalid University, Saudi Arabia.”

Please state what role the funders took in the study.  If the funders had no role, please state: "The funders had no role in study design, data collection and analysis, decision to publish, or preparation of the manuscript."

If this statement is not correct you must amend it as needed.

Please include this amended Role of Funder statement in your cover letter; we will change the online submission form on your behalf.

4. Your ethics statement should only appear in the Methods section of your manuscript. If your ethics statement is written in any section besides the Methods, please delete it from any other section.

5. When completing the data availability statement of the submission form, you indicated that you will make your data available on acceptance. We strongly recommend all authors decide on a data sharing plan before acceptance, as the process can be lengthy and hold up publication timelines. Please note that, though access restrictions are acceptable now, your entire data will need to be made freely accessible if your manuscript is accepted for publication. This policy applies to all data except where public deposition would breach compliance with the protocol approved by your research ethics board. If you are unable to adhere to our open data policy, please kindly revise your statement to explain your reasoning and we will seek the editor's input on an exemption. Please be assured that, once you have provided your new statement, the assessment of your exemption will not hold up the peer review process.

6. If the reviewer comments include a recommendation to cite specific previously published works, please review and evaluate these publications to determine whether they are relevant and should be cited. There is no requirement to cite these works unless the editor has indicated otherwise.

[Note: HTML markup is below. Please do not edit.]

Reviewers' comments:

Reviewer's Responses to Questions

**Comments to the Author**

1. Is the manuscript technically sound, and do the data support the conclusions?

The manuscript must describe a technically sound piece of scientific research with data that supports the conclusions. Experiments must have been conducted rigorously, with appropriate controls, replication, and sample sizes. The conclusions must be drawn appropriately based on the data presented.

Reviewer #1: Yes

Reviewer #2: Yes

**********

2. Has the statistical analysis been performed appropriately and rigorously?

Reviewer #1: I Don't Know

Reviewer #2: Yes

**********

3. Have the authors made all data underlying the findings in their manuscript fully available?

The PLOS Data policy requires authors to make all data underlying the findings described in their manuscript fully available without restriction, with rare exception (please refer to the Data Availability Statement in the manuscript PDF file). The data should be provided as part of the manuscript or its supporting information, or deposited to a public repository. For example, in addition to summary statistics, the data points behind means, medians and variance measures should be available. If there are restrictions on publicly sharing data—e.g. participant privacy or use of data from a third party—those must be specified.

Reviewer #1: Yes

Reviewer #2: Yes

**********

4. Is the manuscript presented in an intelligible fashion and written in standard English?

PLOS ONE does not copyedit accepted manuscripts, so the language in submitted articles must be clear, correct, and unambiguous. Any typographical or grammatical errors should be corrected at revision, so please note any specific errors here.

Reviewer #1: Yes

Reviewer #2: Yes

**********

5. Review Comments to the Author

Please use the space provided to explain your answers to the questions above. You may also include additional comments for the author, including concerns about dual publication, research ethics, or publication ethics. (Please upload your review as an attachment if it exceeds 20,000 characters)

Reviewer #1: The manuscript is written well however few suggestions should be considered,

1. In the introduction section the author provide background but does not clearly articulate the research gap, Why is this study needed in the current context? Lacks updated global and regional statistics (AMR trends, WHO data). No specific hypothesis or research question stated. it should be included in the manuscript.

2. In the methodology section, it was unclear the sampling were random or convenience sampling, there was no justification for sample size (power calculation missing), Inclusion/Exclusion Criteria not clearly defined, CLSI guidelines mentioned but no version/year cited, and no details on quality control strains.

3. In the result section, resistance patterns described, but no molecular characterization (genes, plasmid types), which limits depth.

4. In discussion part, there was no critical comparison with international studies – does not contextualize findings globally. Overlooks important drivers of AMR (e.g., antimicrobial stewardship, infection control). No policy implications or practical recommendations.

Reviewer #2: Dear Authors,

You are doing excellent work, and this manuscript reflects a great deal of effort and thoughtful research. The study is promising and contributes meaningfully to the field. However, there are a few important points that require further clarification and revision to enhance the overall quality and impact of the manuscript.

Line 70–71: Replace “irregular bowel movements” with “diarrhoea or constipation,” which are the clinically accepted descriptions.

Global and regional burden statistics are important, but feel fragmented. For example, lines 72–75 mix global and regional numbers. Consider reorganizing into:

Global burdens (cases & deaths)

Regional burden (South Asia, Pakistan)

Recent outbreaks and XDR reports

Line 79–80: The phrase “driven by an XDR strain, resulting in the emergence of an MDR H58 strain” is confusing. Clarify the chronology: did XDR arise from the H58 lineage?

Line 80, page 3: Terms like “XDR strain,” “MDR H58 strain,” and “XDR bacterial pathogens” are used interchangeably, which may confuse readers. Please define MDR, XDR, and the H58 lineage clearly, and stick to consistent terminology.

Line 83, page 3: I recommend that the authors add the following sentences in their introduction using the mentioned studies:

“The global rise of multidrug-resistant (MDR) microorganisms causing infections is accelerating, with the problem being particularly severe in developing countries.”

Line 83, page 3: It is important to use the update and so related reference according to the above comment:

Rawaa A. Hussein, Shaymaa H. AL-Kubaisy, Mushtak T.S. Al-Ouqaili. The influence of efflux pump, outer membrane permeability and β-lactamase production on the resistance profile of multi, extensively and pandrug-resistant Klebsiella pneumoniae. Journal of Infection and Public Health, Volume 17, Issue 11, 2024. https://doi.org/10.1016/j.jiph.2024.102544. Owaid HA, Al-Ouqaili MTS. Molecular and bacteriological investigations for the co-existence of CRISPR/Cas system and β-lactamases of types extended-spectrum and carbapenemases in Multidrug, extensive drug and Pandrug-Resistant Klebsiella pneumoniae. Saudi J Biol Sci. 2024 Jul;31(7):104022. doi: 10.1016/j.sjbs.2024.104022. Epub 2024 May 19. PMID: 38817398; PMCID: PMC11137337.

Line 90: Typo/grammar issue: “is concerned about” → should be “raises concerns about.”

The role of EPs is well described, but the section (lines 91–103) could be condensed and made more focused. Currently, it mixes resistance, virulence, biofilm, and transporter families without transitions. Suggest splitting:

Efflux pumps in resistance (mechanisms, prevalence)

Efflux pumps in biofilm and virulence

Importance of EPIs

Line 92–93: “bacteria cell” → should be “bacterial cell.”

Line 96, page 4: To enhance the introduction section, add the following reference: Al-Ouqaili MTS, Hussein RA, Kanaan BA, Al-Neda MTS (2025). Investigation of carbapenemase-encoding genes in Burkholderia cepacia and Aeromonas sobria isolates from nosocomial infections in Iraqi patients. PLoS One 20(8): e0315490. https://doi.org/10.1371/journal.pone.0315490

Line 101–102: “Plasma membrane organelles” is inaccurate. ABC transporters are membrane proteins, not organelles.

Line 133, page 5: The section states blood samples were collected, but does not mention the total number of patients, inclusion/exclusion criteria, or demographic details. For clinical studies, this information is critical for reproducibility and ethical transparency.

Line 158, page 6: For susceptibility testing, reference to CLSI guidelines (or EUCAST) is essential for validity currently, yet only Kirby-Bauer and McFarland are mentioned.

Line 217–218, 243–244: While methods are comprehensive, it’s unclear why specific concentrations were chosen (e.g., 0.5–40 μg/mL for MIC; 2× MIC for SEM). A justification (based on literature or preliminary screening) should be provided.

Line 270–271: “Samples showed growth… indicating the presence of bacteria.” → too general; specify “non-lactose fermenting colonies consistent with Salmonella spp.”

Lines 294–299, page 11: Only four isolates were tested (1 MDR, 1 XDR, 2 sensitive). This is too small a sample size to draw robust conclusions about resistance mechanisms, biofilm formation, or EPI activity. Please justify the limited number of isolates, and clearly state this as a limitation.

Line 309–311: Awkward phrasing: “The intensity of fluorescence was lowly detectable in MDR and undetectable in XDR…” → Suggest “Fluorescence intensity was weak in MDR and absent in XDR isolates.”

Line 321–322 (Figure 3): The figure is not clear enough to convincingly demonstrate fluorescence differences among MDR, XDR, and sensitive strains. Please consider:

Line 325–331: Grammar: “it gets expelled” → “it is expelled.”

Lines 337–349; 400–413; 465–471: P-values are present, but statistical test details are missing (e.g., t-test, ANOVA, repeated measures?). Must specify the test used, replicates (n), and confidence intervals where possible.

Increasing image resolution and brightness/contrast to make fluorescence intensity more distinguishable.

Adding a clear scale bar and indicating the exposure time/UV source used for visualisation.

Including quantification of fluorescence intensity (e.g., densitometry or ImageJ analysis) to support the visual observations.

Providing side-by-side comparison panels with labelled arrows or annotations highlighting key differences between strains.

Using a negative control plate without EtBr to eliminate background signals.

Line 368–373: Biofilm OD classification should be consistent with the cutoff criteria given in Methods (weak, moderate, strong). Currently, the interpretation looks inconsistent.

Lines 389–406, page 16: MIC results are vague (“45–50% inhibition at 10–20 μg/mL”). By definition, MIC should be the lowest concentration that prevents visible growth. The current description looks more like IC₅₀ values. Please clarify methodology and correct terminology.

Line 490–494: Resistance results are mentioned, but not linked strongly to clinical relevance. The discussion would benefit from contextualization — e.g., how the resistance profile in your isolates compares quantitatively with national or WHO surveillance data.

Line 503–511: While SA and SB effects are well described, the statement “Our results clearly demonstrated EPI activities” could be strengthened by discussing limitations (e.g., in vitro only, lack of in vivo validation). Otherwise, the claims may sound overstated.

Line 515, page 27: To enhance the discussion section, add the following reference: Al-Ouqaili, M.T.S., Al-Kubaisy, S.H.M., Al-Ani, N.F.I. Biofilm antimicrobial susceptibility pattern for selected antimicrobial agents against planktonic and sessile cells of clinical isolates of staphylococci using MICs, BICs and MBECs. Asian Journal of Pharmaceutics, volume 12, Issue 4, October-December 2018, Pages S1375-S1383. https://doi.org/10.22377/ajp.v12i04.2938.

Line 576: The Conclusion should be objective, with further perspective, or should add at least a few sentences about future study/future perspective of it

**********

6. PLOS authors have the option to publish the peer review history of their article (what does this mean?). If published, this will include your full peer review and any attached files.

If you choose “no”, your identity will remain anonymous but your review may still be made public.

**Do you want your identity to be public for this peer review?**  For information about this choice, including consent withdrawal, please see our Privacy Policy.

Reviewer #1: **Yes:** Muhammad Usman Qamar

Reviewer #2: **Yes:** Mushtak, T.S.Al-Ouqaili

**********

[NOTE: If reviewer comments were submitted as an attachment file, they will be attached to this email and accessible via the submission site. Please log into your account, locate the manuscript record, and check for the action link "View Attachments". If this link does not appear, there are no attachment files.]

While revising your submission, please upload your figure files to the Preflight Analysis and Conversion Engine (PACE) digital diagnostic tool, https://pacev2.apexcovantage.com/. PACE helps ensure that figures meet PLOS requirements. To use PACE, you must first register as a user. Registration is free. Then, login and navigate to the UPLOAD tab, where you will find detailed instructions on how to use the tool. If you encounter any issues or have any questions when using PACE, please email PLOS at figures@plos.org. Please note that Supporting Information files do not need this step.

Attachment Submitted filename: Reviewer comments- PONE-D-25-32847_reviewer.pdf

# Author comment: Author response to Decision Letter 1

**DOI:** 10.1371/journal.pone.0347214.r002

25 Nov 2025

We thank the Editor and Reviewers for their constructive comments. We have revised the manuscript accordingly

Attachment Submitted filename: Response to Reviewers.docx

# Peer review: Decision Letter 1

**DOI:** 10.1371/journal.pone.0347214.r003

**Authors:** Leonard Ighodalo Uzairue

5 Mar 2026

PONE-D-25-32847R1

Schisandrins as Novel Efflux Pumps Inhibitors and Non-Antibiotic Compounds Against Multi- and Extensively-Drug Resistant Clinical strains of Salmonella typhi:  An In-Vitro Study

PLOS One

Dear Dr. Rana,

Thank you for submitting your manuscript to PLOS ONE. After careful consideration, we feel that it has merit but does not fully meet PLOS ONE’s publication criteria as it currently stands. Therefore, we invite you to submit a revised version of the manuscript that addresses the points raised during the review process.

Please submit your revised manuscript by Apr 19 2026 11:59PM. If you will need more time than this to complete your revisions, please reply to this message or contact the journal office at plosone@plos.org. When you're ready to submit your revision, log on to https://www.editorialmanager.com/pone/ and select the 'Submissions Needing Revision' folder to locate your manuscript file.

Please include the following items when submitting your revised manuscript:

- A letter that responds to each point raised by the academic editor and reviewer(s). You should upload this letter as a separate file labeled 'Response to Reviewers'.
- A marked-up copy of your manuscript that highlights changes made to the original version. You should upload this as a separate file labeled 'Revised Manuscript with Track Changes'.
- An unmarked version of your revised paper without tracked changes. You should upload this as a separate file labeled 'Manuscript'.

-->If you would like to make changes to your financial disclosure, please include your updated statement in your cover letter. Guidelines for resubmitting your figure files are available below the reviewer comments at the end of this letter.

If applicable, we recommend that you deposit your laboratory protocols in protocols.io to enhance the reproducibility of your results. Protocols.io assigns your protocol its own identifier (DOI) so that it can be cited independently in the future. For instructions see: https://journals.plos.org/plosone/s/submission-guidelines#loc-laboratory-protocols. Additionally, PLOS ONE offers an option for publishing peer-reviewed Lab Protocol articles, which describe protocols hosted on protocols.io. Read more information on sharing protocols at https://plos.org/protocols?utm_medium=editorial-email&utm_source=authorletters&utm_campaign=protocols.

We look forward to receiving your revised manuscript.

Kind regards,

Leonard Ighodalo Uzairue, PhD

Academic Editor

PLOS One

Journal Requirements:

If the reviewer comments include a recommendation to cite specific previously published works, please review and evaluate these publications to determine whether they are relevant and should be cited. There is no requirement to cite these works unless the editor has indicated otherwise.

Please review your reference list to ensure that it is complete and correct. If you have cited papers that have been retracted, please include the rationale for doing so in the manuscript text, or remove these references and replace them with relevant current references. Any changes to the reference list should be mentioned in the rebuttal letter that accompanies your revised manuscript. If you need to cite a retracted article, indicate the article’s retracted status in the References list and also include a citation and full reference for the retraction notice.

Additional Editor Comments:

Thank you for the great responses to the reviewers' comments. Can you please respond to the few comments raised on data availability and also the appropriate use of units? If there is any reason why the data cannot be made available, you need to make that available to us.

[Note: HTML markup is below. Please do not edit.]

Reviewers' comments:

Reviewer's Responses to Questions

**Comments to the Author**

1. If the authors have adequately addressed your comments raised in a previous round of review and you feel that this manuscript is now acceptable for publication, you may indicate that here to bypass the “Comments to the Author” section, enter your conflict of interest statement in the “Confidential to Editor” section, and submit your "Accept" recommendation.

Reviewer #3: All comments have been addressed

Reviewer #4: All comments have been addressed

**********

2. Is the manuscript technically sound, and do the data support the conclusions?

The manuscript must describe a technically sound piece of scientific research with data that supports the conclusions. Experiments must have been conducted rigorously, with appropriate controls, replication, and sample sizes. The conclusions must be drawn appropriately based on the data presented.

Reviewer #3: Yes

Reviewer #4: Yes

**********

3. Has the statistical analysis been performed appropriately and rigorously?

Reviewer #3: Yes

Reviewer #4: Yes

**********

4. Have the authors made all data underlying the findings in their manuscript fully available?

The PLOS Data policy requires authors to make all data underlying the findings described in their manuscript fully available without restriction, with rare exception (please refer to the Data Availability Statement in the manuscript PDF file). The data should be provided as part of the manuscript or its supporting information, or deposited to a public repository. For example, in addition to summary statistics, the data points behind means, medians and variance measures should be available. If there are restrictions on publicly sharing data—e.g. participant privacy or use of data from a third party—those must be specified.

Reviewer #3: Yes

Reviewer #4: No

**********

5. Is the manuscript presented in an intelligible fashion and written in standard English?

PLOS ONE does not copyedit accepted manuscripts, so the language in submitted articles must be clear, correct, and unambiguous. Any typographical or grammatical errors should be corrected at revision, so please note any specific errors here.

Reviewer #3: Yes

Reviewer #4: Yes

**********

6. Review Comments to the Author

Please use the space provided to explain your answers to the questions above. You may also include additional comments for the author, including concerns about dual publication, research ethics, or publication ethics. (Please upload your review as an attachment if it exceeds 20,000 characters)

Reviewer #3: The revised manuscript is almost ready for publication. I have only a few minor comments/suggestions:The most important one: please add units and proper labels to the Y-axes of Figures 6 and 9.

In Figure 7, why does growth recording begin at 2 h rather than at the initial time (t = 0)? It is important to demonstrate that all cultures start with approximately the same absorbance (OD) value.

In Figure 9, the antibiofilm activity appears very potent already at the lowest concentration tested (3 µg/mL) and shows little to no further increase at higher concentrations. It would be valuable to test 1–2 lower concentrations (e.g., 0.5–1 µg/mL) to better characterize the dose-response relationship.

An evaluation of whether the tested compounds are bactericidal or bacteriostatic could have been performed using bacterial live/dead staining.

“Gram” should be capitalized (e.g., Gram-positive, Gram-negative).

There are many missing spaces between words, especially after references (e.g., line 538: “recently(53)” should be “recently (53)”). Please also correct any remaining double spaces throughout the manuscript.

Reviewer #4: Thank you for the revised manuscript and the comprehensive point-by-point rebuttal. The revisions have significantly strengthened the paper. The investigation into Schisandrins as novel efflux pump inhibitors against MDR and XDR strains of Salmonella typhi addresses a critical and growing challenge in the global fight against antimicrobial resistance. The updated discussion, which now thoughtfully incorporates regional and global AMR surveillance context, adds valuable clinical and public health perspective to these in-vitro findings.

The methodological approaches, including the time-kill, EtBr-agar cartwheel, and antibiofilm assays, are technically sound. The statistical analyses have been performed appropriately and rigorously. Furthermore, I appreciate the transparency in the revised discussion regarding the study's limitations; explicitly acknowledging the reliance on in-vitro assays and the necessity for future molecular characterization and in-vivo validations prevents overstatement of the findings and provides a clear direction for future research. The language and writing quality are clear, intelligible, and meet the required standards.

There is, however, one remaining issue regarding journal policy that must be resolved prior to final acceptance:

1. Data Availability Requirement: In the current manuscript, the data availability statement indicates that data is available "upon reasonable request." Please note that the PLOS Data policy strictly requires all data underlying the findings to be fully available without restriction. Stating that data is available upon request is not sufficient. You must upload the minimal raw dataset (e.g., the underlying spreadsheet data behind your graphs, raw CFU counts, and uncropped images) as part of the manuscript's Supporting Information files or deposit it into a recognized public repository (providing the DOI/accession number).

Once this data availability requirement is fully compliant with journal guidelines, the manuscript will be acceptable for publication. The scientific revisions in this round were handled excellently.

**********

7. PLOS authors have the option to publish the peer review history of their article (what does this mean?). If published, this will include your full peer review and any attached files.

If you choose “no”, your identity will remain anonymous but your review may still be made public.

**Do you want your identity to be public for this peer review?**  For information about this choice, including consent withdrawal, please see our Privacy Policy.

Reviewer #3: **Yes:** Rodolfo Garcia Contreras

Reviewer #4: No

**********

[NOTE: If reviewer comments were submitted as an attachment file, they will be attached to this email and accessible via the submission site. Please log into your account, locate the manuscript record, and check for the action link "View Attachments". If this link does not appear, there are no attachment files.]

To ensure your figures meet our technical requirements, please review our figure guidelines: https://journals.plos.org/plosone/s/figures

You may also use PLOS’s free figure tool, NAAS, to help you prepare publication quality figures: https://journals.plos.org/plosone/s/figures#loc-tools-for-figure-preparation.

NAAS will assess whether your figures meet our technical requirements by comparing each figure against our figure specifications.

# Author comment: Author response to Decision Letter 2

**DOI:** 10.1371/journal.pone.0347214.r004

24 Mar 2026

We sincerely thank the reviewers for their encouraging remarks for recognizing our effort. Thank you for your positive and encouraging feedback. We sincerely appreciate the reviewer’s thoughtful evaluation of our manuscript and the recognition of the revisions made.Your valuable comments and suggestions have helped us further strengthen the quality and clarity of our work

Attachment Submitted filename: Response_to_Reviewers_auresp_2.docx

# Editor report: Decision Letter 2

**DOI:** 10.1371/journal.pone.0347214.r005

**Authors:** Leonard Ighodalo Uzairue

30 Mar 2026

Schisandrins as Novel Efflux Pumps Inhibitors and Non-Antibiotic Compounds Against Multi- and Extensively-Drug Resistant Clinical strains of Salmonella typhi:  An In-Vitro Study

PONE-D-25-32847R2

Dear Dr. Rana,

We’re pleased to inform you that your manuscript has been judged scientifically suitable for publication and will be formally accepted for publication once it meets all outstanding technical requirements.

Within one week, you’ll receive an e-mail detailing the required amendments. When these have been addressed, you’ll receive a formal acceptance letter and your manuscript will be scheduled for publication.

An invoice will be generated when your article is formally accepted. Please note, if your institution has a publishing partnership with PLOS and your article meets the relevant criteria, all or part of your publication costs will be covered. Please make sure your user information is up-to-date by logging into Editorial Manager at Editorial Manager® and clicking the ‘Update My Information' link at the top of the page. For questions related to billing, please contact billing support.

If your institution or institutions have a press office, please notify them about your upcoming paper to help maximize its impact. If they’ll be preparing press materials, please inform our press team as soon as possible -- no later than 48 hours after receiving the formal acceptance. Your manuscript will remain under strict press embargo until 2 pm Eastern Time on the date of publication. For more information, please contact onepress@plos.org.

Kind regards,

Leonard Ighodalo Uzairue, PhD

Academic Editor

PLOS One

Additional Editor Comments (optional):

Reviewers' comments:

# Editor report: Acceptance letter

**DOI:** 10.1371/journal.pone.0347214.r006

**Authors:** Leonard Ighodalo Uzairue

PONE-D-25-32847R2

PLOS One

Dear Dr. Rana,

I'm pleased to inform you that your manuscript has been deemed suitable for publication in PLOS One. Congratulations! Your manuscript is now being handed over to our production team.

At this stage, our production department will prepare your paper for publication. This includes ensuring the following:

* All references, tables, and figures are properly cited

* All relevant supporting information is included in the manuscript submission,

* There are no issues that prevent the paper from being properly typeset

You will receive further instructions from the production team, including instructions on how to review your proof when it is ready. Please keep in mind that we are working through a large volume of accepted articles, so please give us a few days to review your paper and let you know the next and final steps.

Lastly, if your institution or institutions have a press office, please let them know about your upcoming paper now to help maximize its impact. If they'll be preparing press materials, please inform our press team within the next 48 hours. Your manuscript will remain under strict press embargo until 2 pm Eastern Time on the date of publication. For more information, please contact onepress@plos.org.

You will receive an invoice from PLOS for your publication fee after your manuscript has reached the completed accept phase. If you receive an email requesting payment before acceptance or for any other service, this may be a phishing scheme. Learn how to identify phishing emails and protect your accounts at https://explore.plos.org/phishing.

If we can help with anything else, please email us at customercare@plos.org.

Thank you for submitting your work to PLOS ONE and supporting open access.

Kind regards,

PLOS ONE Editorial Office Staff

on behalf of

Dr. Leonard Ighodalo Uzairue

Academic Editor

PLOS One
